# Fine-Tuning of Molecular Structures to Generate Carbohydrate Based Super Gelators and Their Applications for Drug Delivery and Dye Absorption

**DOI:** 10.3390/gels7030134

**Published:** 2021-09-07

**Authors:** Jonathan Bietsch, Mary Olson, Guijun Wang

**Affiliations:** Department of Chemistry and Biochemistry, Old Dominion University, Norfolk, VA 23529, USA; jbietsch@odu.edu (J.B.); maryaolson@vt.edu (M.O.)

**Keywords:** supramolecular gels, low molecular weight gelator (LMWGs), hydrogelators, organogelators, hydrogels, organogels, carbohydrates, glucosamine

## Abstract

Carbohydrate-based low molecular weight gelators (LMWGs) exhibit many desirable properties making them useful in various fields including applications as drug delivery carriers. In order to further understand the structural connection to gelation properties, especially the influence of halide substitutions, we have designed and synthesized a series of *para*-chlorobenzylidene acetal protected D-glucosamine amide derivatives. Fifteen different amides were synthesized, and their self-assembling properties were assessed in multiple organic solvents, as well as mixtures of organic solvents with water. All derivatives were found to be gelators for at least one solvent and majority formed gels in multiple solvents at concentrations lower than 2 wt%. A few derivatives rendered remarkably stable gels in aqueous solutions at concentrations below 0.1 wt%. The benzamide **13** formed gels in water and in EtOH/H_2_O (*v*/*v* 1:2) at 0.36 mg/mL. The gels were characterized using optical microscopy and atomic force microscopy, and the self-assembly mechanism was probed using variable temperature ^1^H-NMR spectroscopy. Gel extrusion studies using H_2_O/DMSO gels successfully printed lines of gels on glass slides, which retained viscoelasticity based on rheology. Gels formed by the benzamide **13** were used for encapsulation and the controlled release of chloramphenicol and naproxen, as well as for dye removal for toluidine blue aqueous solutions.

## 1. Introduction

Carbohydrate-based low molecular weight gelators (LMWGs) are small molecules with the ability to self-assemble into three dimensional structures that entrap surrounding solvent, creating solid-like materials known as supramolecular gels. The self-assembly process occurs through a balanced combination of non-covalent interactions such as hydrogen bonding, π-π interactions, van der Waals forces, halogen bonding, and hydrophobic interactions [1,2,3]. LMWG systems typically form one dimensional self-assembled fibers, which interact with each other to create intricate networks. The surface tension of the solvent causes it to become immobilized within the intricate network of fibers, creating the supramolecular gel [3,4]. In recent years, the design and study of sugar based gelators have attracted much attention. They have been explored for applications in several research fields ranging from drug delivery [5,6,7,8], to environmental applications [9,10,11,12,13,14], enzyme immobilization, ion sensing [15,16,17], and supramolecular gel catalysts [18,19,20].

Carbohydrates have become an attractive feedstock for the design and synthesis of LMWGs because they are an inexpensive, commercially available renewable resources, with high biocompatibility and low environmental impact [1,21,22,23]. Using glucose and glucosamine as the building blocks, several series of effective gelators have been designed and synthesized [24,25,26,27]. These small molecule-based gelators show great potential for various applications, especially for sustained release of naproxen and other anti-inflammatory drugs. We have been interested in the discovery of LMWGs that are effective at low concentrations for a variety of solvents. In the structure-based design, the functionalization of different positions of glucosamine derivatives have been studied. Among the many current reported gelators, few of them have minimum gelation concentrations (MGCs) lower than 0.1 wt%. LMWGs that can form gels at concentrations below 0.1 wt% are defined as super gelators [12,28,29,30]. Gels formed from super gelators may have enhanced biocompatibility due to the reduction in the amount of compound required for gel formation. These super gelators may be useful for several applications including drug delivery, oil spill clean-up [31], materials for 3D printing and tissue engineering, and as matrices for catalyst and enzyme immobilizations. 

In the rational design of LMWG, various functional groups capable of different non-covalent interactions are utilized in specific spatial arrangements to increase the gelation properties of a molecule. While hydrogen bonding groups, aromatic groups and hydrophobic aliphatic groups are frequently used in the rational design of gelators, there are several examples in literature of the addition of an aromatic halogen group leading to enhanced gelation properties [32,33,34,35,36]. The halogen moiety has the ability to act as an electron acceptor, which leads to strong non-covalent interactions with both neutral and negatively charged species capable of donating electrons [37]. Because these strong non-covalent interactions have a similar strength to hydrogen bonding, they are termed “halogen bonding” [37]. Halogen bonding can be a powerful tool in driving the self-assembly of supramolecular systems, as well as stabilizing the supramolecular microstructures [37]. Recently, several iodo-substituted triazole derivatives and vicinal dibromo-substituted benzene derivatives have demonstrated the importance of halogen bonding in organogel formation [38,39].

**Figure 1:**
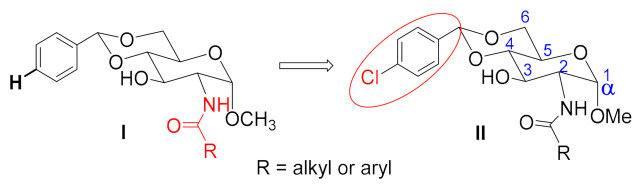
Design rationale of effective sugar-based LMWGs.

We have been working on the structure-based design of LMWGs using sugar templates and exploring the applications of these gelators in different research fields. Using structure-based design methods, many different glucosamine derivatives have been reported as effective gelators over the recent years [24,26,27,40,41,42,43]. Previously, we observed that certain 4,6-benzylidene acetal protected d-glucosamine derivatives based on structure I (Figure 1) were effective organogelators and hydrogelators [26]. In this study, we introduced a chloro-substituent to the 4,6-benzylidene-protected glucosamine system and analyzed the effect this substitution had towards molecular assembly and gelation properties. The general structure II, which contains a chloro substituent in the *para* position of the 4,6-benzylidene protective moiety, was envisioned to have enhanced intermolecular interactions which may lead to effective LMWGs. 

## 2. Results and Discussion

**Scheme 1:**
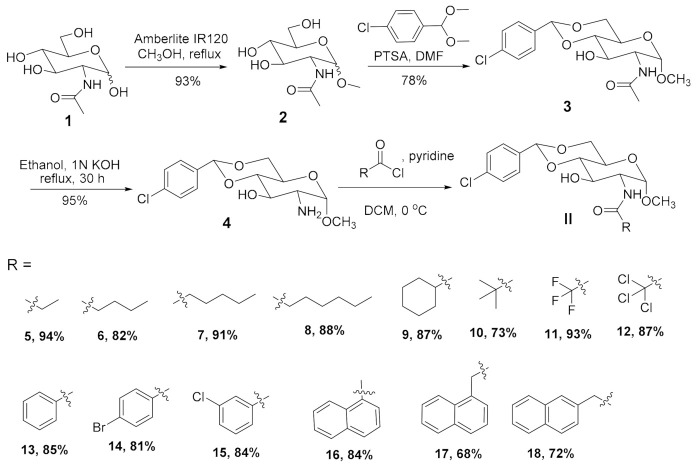
Synthesis of 4-chlorobenzylidene acetal protected glucosamides.

**Figure 2:**
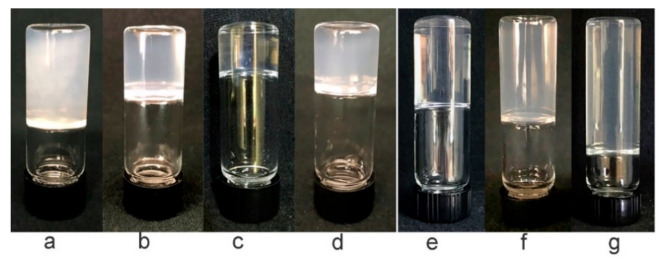
Representative gels formed by compounds **6**, **7**, **11**, **12** in 1-dram vials (**a**–**d**) and **13** in 2-dram vials (**e**–**g**). (**a**) shows an opaque gel, (**b**) and (**d**) show translucent gels, the rest are transparent gels. (**a**) **6** in EtOH/H_2_O (1:1) at 0.74 mg/mL; (**b**) **7** in DMSO/H_2_O (1:1) at 0.91 mg/mL; (**c**) **11** in EtOH/H_2_O (1:1) at 2.2 mg/mL; (**d**) **12** in EtOH/H_2_O (1:2) at 0.91 mg/mL; (**e**–**g**) compound **13**, (**e**) EtOH/H_2_O (1:1) at 0.54 mg/mL; (**f**) H_2_O at 0.40 mg/mL; (**g**) EtOH/H_2_O (1:2) at 0.36 mg/mL.

The synthesis of the 4,6-*O*-(*p*-chlorobenzylidene) acetal-functionalized d-glucosamine amide derivatives is shown in Scheme 1. The starting material *N*-acetylglucosamine **1** was converted to the O-methylated compound **2** through the acid catalyzed glycosylation reaction. Compound **2** then underwent a 4,6-protection with chlorobenzylidene dimethyl acetal, yielding compound **3**. Deacetylation of compound **3** produced the headgroup, compound **4**. Acylation of the headgroup **4** with different acid chlorides afforded a series of fourteen different amide derivatives, including eight aliphatic derivatives **5**–**12** and six containing aromatic groups **13**–**18**. The aliphatic amides include three branched derivatives, the tertiary trifluoro-, trichloro-, and trimethyl derivatives **10**–**12**, four primary derivatives **6**–**8**, and a secondary cyclohexyl carboxamide **9**. The aromatic derivatives include three mono-benzene derivatives and three naphthyl derivatives. These diverse functional groups can allow us to probe the effect of the different acyl chains towards molecular self-assembling. These derivatives were tested in multiple solvents to determine their effectiveness as LMWGs and the results are shown in Table 1. Several representative gel photos are shown in Figure 2. 

The solvents tested include organic solvents such as hexane, toluene, six different alcohols, water, ethanol and water mixtures, and DMSO and water mixtures. As shown in Table 1, to our delight, all derivatives showed effective gelation for at least one of the tested solvents. The best performing compound was cyclohexyl derivative **9**, which formed gels in eleven out of the twelve tested solvents! We found that most derivatives were insoluble in hexanes except the *t*-butyl amide derivative **10**, which formed gels in hexane with a MGC of 5.0 mg/mL. These compounds were all also found to be insoluble in water at 20 mg/mL, except the benzoate compound **13**, a highly efficient hydrogelator which formed a translucent hydrogel at 0.36 mg/mL (0.04 wt%). The short chain alkyl derivatives containing one to six carbons, compounds **3** and **5**–**8**, formed gels in toluene, and several alcohols. They also formed gels in DMSO/H_2_O (*v*/*v*, 1:1 and 1:2) at very low MGCs. The methyl amide **3** is surprisingly a very versatile gelator, much like the cyclohexyl derivative **9**, forming gels in eleven of the tested solvents. Increasing the aliphatic chain by one carbon resulted in reduction in the gelation tendency, with the ethyl derivative **5** being a much less versatile gelator, which only formed gels in four different alcohols at higher concentrations. Increasing the aliphatic chain further restored the gelation properties, with the amide **6** being a very efficient gelator, which formed gels in nine of the tested solvents. It was highly efficient in solidifying alcohols and aqueous mixtures, with a MGC of 0.74 mg/mL in ethanol water (1:1) and 1.0 mg/mL in both *n*-butanol and DMSO/H_2_O (1:2). The pentyl and hexyl amides **7** and **8** formed gels in toluene, several alcohols, and exhibited very low MGCs in DMSO water mixtures. The cyclohexyl derivative **9**, with increased steric hindrance of the aliphatic group, resulted in a much more effective gelator than the straight chained six carbon analog. It formed gels in eleven solvents listed in the table and at concentrations typically less than 0.7 wt%. 

Further increasing the steric hindrance to the *t*-butyl amide **10** resulted in full solubility in most of the alcohols and only formed gels in ethylene glycol, glycerol, and 1:1 mixtures of water with DMSO or ethanol at 20 mg/mL. Interestingly, this compound formed opaque gels in hexane at concentrations of 5.0 mg/mL and was the only gelator in these analogs that was capable of immobilizing hexanes. The trichloromethyl derivative **12** showed more effective gelation properties for ethanol water mixtures, and DMSO water mixtures at low MGCs in comparison to the t-butyl amide **10**. Like the t-butyl derivative, compound **12** is also soluble in toluene and most alcohols. The two molecules are similar in size since the Cl atom has a similar van der Walls radius to the CH_3_ group, therefore it is reasonable to predict that the two compounds have similar solubility and gelation patterns. Similarly, we can expect that the trifluoro derivative **11** should be comparable to compound **3**, since the CF_3_ and CH_3_ have a similar size (C–F 1.325 Å, C-H 1.113 Å, radius F 42 pm, H 53 pm). Similar to compound **3**, the trifluoro derivative **11** is also a very versatile gelator. The CF_3_ analog formed gels in eight solvents, but at much lower MGCs in the aqueous mixtures. The tendency observed here, using the molecules’ 3-dimensional size to predict gelation behavior, may be a useful strategy in the designing and development of future effective supramolecular gelators. 

Among the aromatic derivatives **13**–**18**, the most versatile gelators are the chlorophenyl amide **15** and the benzamide **13**, which formed gels in ten and nine of the tested solvents. Compound **15** performed most efficiently in the mixed solvents with MGCs up to 0.7 mg/mL. Compound **13** is a versatile gelator as well, forming gels in ethanol water mixtures and DMSO water mixtures with the lowest MGCs, below 0.5 mg/mL. Benzamide **13** did not form gels in isopropanol, n-propanol, and n-butanol. In contrast, 3-chlorobenzamide **15** formed gels in these three alcohols, as well as seven other solvents, including aqueous mixtures with DMSO or ethanol. The performance of chlorobenzamide **15** compared to compound **13** indicated that the additional chloro substituent did not affect gelation properties. On the other hand, the introduction of the bulkier halogen such as a bromo group in compound **14**, apparently dismissed the gelation tendencies, with the compound only forming gels in four solvents at relatively high MGCs. The naphthyl derivative **16** is a quite versatile gelator, which formed gels in 8 different solvents, including ethanol and DMSO water (1:1 and 1:2) mixtures. 

The results from the bulkier functional groups, such as the bromo compound **14** and the naphthyl derivatives **16** and **17**, indicated that steric hindrance and/or very strong intermolecular forces may be a detrimental factor towards gelation. The results from the branched derivatives (**11**, **12**, **10**) and the methyl amide **3** indicated that the overall molecular size is an important parameter towards predicting the gelation tendencies of an unknown compound within a similar template. The CF_3_ and CH_3_ derivatives are both more versatile gelators than the larger CCl_3_ and C(CH_3_)_3_ derivatives. In addition, for aqueous mixtures, the CF_3_ and CCl_3_ amides are more effective than the alkyl derivatives, forming gels at much lower concentrations. These again showed that halogen substituents enhanced intermolecular interactions and their employment can result in effective gelators. 

**Figure 3:**
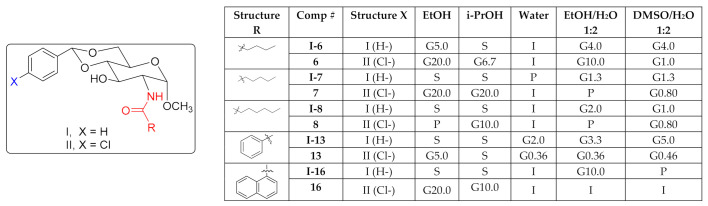
Comparison of gelation properties for several gelators with different functional groups.

Figure 3 shows the gelation properties of several 4,6-benzylidene acetal derivatives (I) reported previously [26] for comparison with the chloro-derivatives (II). The compounds with 5–7 straight chain amides (compounds **6**–**8**) are more effective gelators than the corresponding derivatives **I-6**, **I-7**, and **I-8**. The benzamide **13** and naphthyl amide **16** are also more efficient gelators than compounds **I-13** and **I-16**, respectively. Compound **13** formed gels in water, ethanol water mixtures, and DMSO water mixtures at much lower concentrations than compound **I-13**. This trend was also observed for naphthyl derivative **16**, with it forming gels in isopropanol, ethanol, and DMSO water (1:2), while compound **I-16** did not form gels in any of these three solvents.

**Figure 4:**
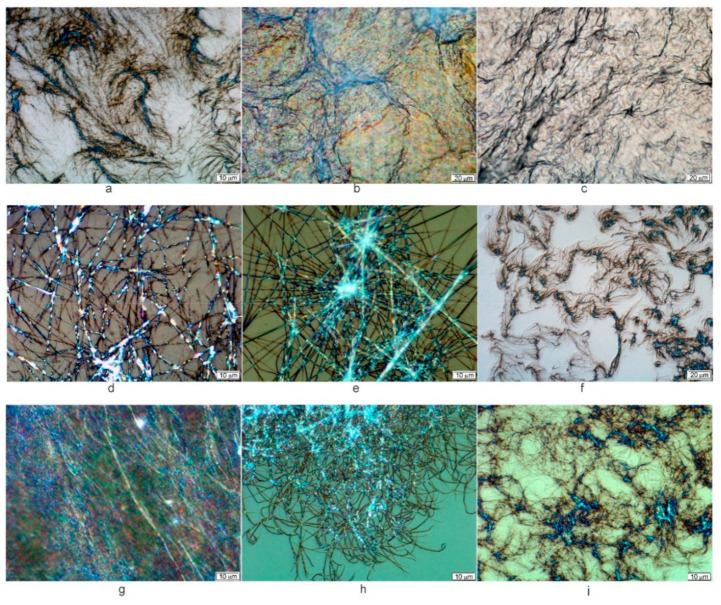
Optical micrographs of the gels formed by several gelators. (**a**,**b**) compound **6** in EtOH/H_2_O (*v*/*v* 1:1) at 0.74 mg/mL; (**c**,**d**) compound **7** in DMSO/H_2_O (*v*/*v* 1:1) at 0.91 mg/mL; (**e**) amide **11** in DMSO/H_2_O (*v*/*v* 1:1) at 2.9 mg/mL; (**f**) amide **12** in EtOH/H_2_O (*v*/*v* 1:1) at 2.0 mg/mL; (**g**–**i**) are the gels formed by compound **13** in: (**g**) water at 0.40 mg/mL; (**h**) DMSO/H_2_O (*v*/*v* 1:1) at 1.0 mg/mL, (**i**) EtOH/H_2_O (*v*/*v* 1:1) at 0.74 mg/mL.

Several selected gels were characterized using optical microscopy and atomic force microscopy. As shown in Figure 4, the optical micrographs (OMs) of the gels show typical fibrous networks exhibited by LMWGs [26]; however, many of the aggregates appeared as curved fibers rather than straight fibers. The gel formed by compound **6** in EtOH/H_2_O (1:1) at 0.74 mg/mL showed very thin hair like curved fibrous assemblies in the outer edge region of the gel (Figure 4a) and more smooth film type morphology with larger fibers on the surface towards the denser interior region of the gel (Figure 4b). The gel of **7** in DMSO/H_2_O (1:1) showed similar film like morphologies, with fibers drying on top of the bulk gel (Figure 4c), but at the edge of the gel where the fibers were much less dense, distinctive individual fibers can be observed (Figure 4d). These fibers have a slightly larger diameter compared to the fibers observed from the gel in Figure 4a. Amide **11** in DMSO/H_2_O (1:1) at concentrations above MGC show long straight fibers which seem to overlap at a central core, creating star shaped morphologies (Figure 4e), which could be due to a certain nucleation process. The gel formed by amide **12** in EtOH/H_2_O (1:1) at 2.0 mg/mL (Figure 4f) showed very thin hair like fiber assemblies, similar to other ethanol water gels. The most efficient gelator in the series, compound **13**, formed long fibrous assemblies as well. The hydrogel showed long and narrow fibrous features (Figure 4g) and the gels formed in DMSO:H_2_O (1:1) showed intertwined fibers (Figure 4h), along with helices formations. Helical structures are not surprising, since these gelators are based on a chiral glucosamine headgroup and helices formations like these have been previously reported in literature [42]. Lastly, the ethanol water gel formed by **13** showed very thin and curved fibrous assemblies (Figure 4i). 

**Figure 5:**
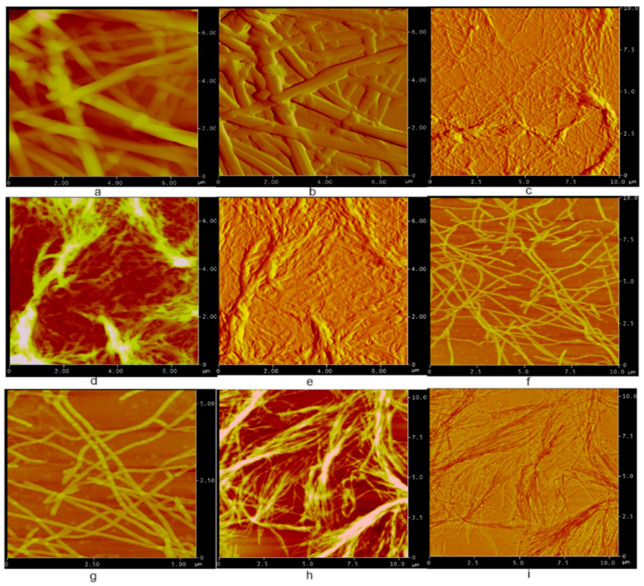
AFM images of the gels formed by gelators **11**–**13**, (**a**,**d**,**f**,**g**,**h**) are height images, and the rest are phase images. (**a**,**b**) **11** in DMSO/H_2_O (*v*/*v* 1:2) at 2.0 mg/mL; (**c**–**e**) **12** in EtOH/H_2_O (*v*/*v* 1:1) at 2.0 mg/mL; (**f**,**g**) **13** in DMSO/H_2_O (*v*/*v* 1:1) at 1.0 mg/mL; (**h**,**i**) **13** in EtOH/H_2_O (*v*/*v* 1:1) at 1.0 mg/mL.

Atomic force microscopy (AFM) was also used to characterize the morphologies of the gels formed by several gelators (Figure 5). The AFM images of gels formed by compound **11** in DMSO/H_2_O (*v*/*v* 1:2) showed fibrous or tubular networks containing cylindrical tubules around 0.5 µm in diameter (Figure 5a,b). This morphology is consistent with the morphology observed through optical imaging. Much thinner fibrillar networks with fibers that are typically more intertwined and curved were observed for the gel of compound **12** in EtOH/H_2_O (1:1) at 2.0 mg/mL, as shown in Figure 5c–e. The AFM images of the gels formed by compound **13** in DMSO/H_2_O (1:1) at 1.0 mg/mL (Figure 5f,g), show the gelator formed a fibrous network which is composed of more round curved fibers and helices. These fibers have a smaller diameter, typically less than 0.2 µm. The morphology of the gel by gelator **13** in EtOH/H_2_O (1:1) at 1.0 mg/mL appeared as fibrous assemblies, which are typically bundled together to form fan shaped organizations (Figure 5h,i).

**Figure 6:**
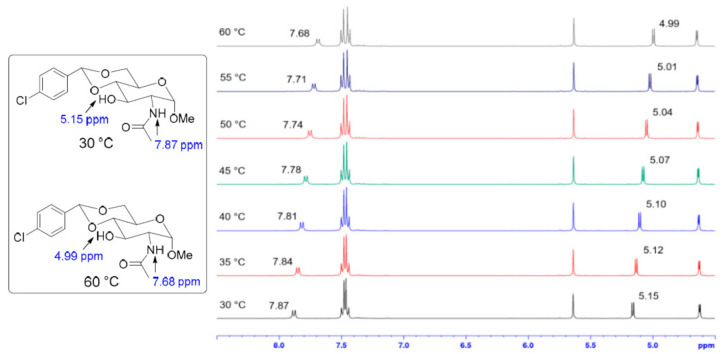
Stacked ^1^H-NMR (400 MHz) spectra of compound **3** at temperature 30–60 °C. The sample was prepared at 12.5 mg/mL in DMSO-d_6_.

**Figure 7:**
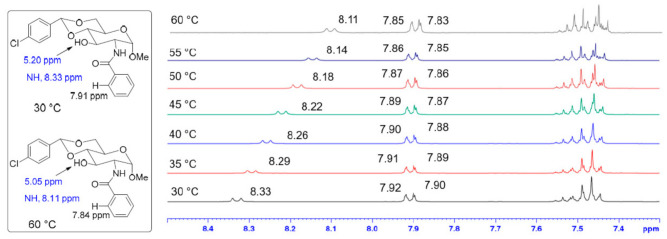
Stacked ^1^H-NMR spectra of compound **13** at temperature 30–60 °C from 7.4 to 8.5 ppm. Sample was prepared at 10.0 mg/mL in DMSO-d_6_.

From the gelation properties discussion above, it is clear that the introduction of a chloro substituent to the phenyl ring has contributed favorably towards molecular self-assembly and gelation. In order to further elucidate the gelation mechanism, we studied the ^1^H-NMR spectra of compounds **3**, **11** and **13** at different temperatures in DMSO-d_6_. Various regions are examined in Figure 6 and Figure 7, and the full range of each spectrum is available in ESI Figures S1–S3. The chemical shifts of the aliphatic C–H protons in compound **3** did not change when the temperature was increased from 30 to 60 °C. A significant upfield shift was observed for both the amide NH signal (0.19 ppm) and the OH signal (0.16 ppm). These indicated that hydrogen bonding played an important role in the molecule assembly process. The upfield shift at higher temperatures reflect the effect of reducing the concentration of intermolecular hydrogen bonds. Interestingly, the aromatic ring showed chemical shift changes, as shown in Figure S1b. With increasing temperatures, the ortho and meta proton signals move in opposing directions from a central point. This change in coupling patterns and chemical shifts of the aromatic protons indicate that the aromatic ring is partaking in intermolecular interactions and plays an important role in the formation of molecular assembly. 

The ^1^H-NMR spectra of compound **11** at different temperatures (Figure S2a–c) show similar patterns to those of compound **3**, but with larger chemical shift changes. For instance, a larger change in chemical shift of 0.23 ppm was observed for the amide proton at higher temperatures. From 30–60 °C, the NH peak shifted from 9.56 ppm to 9.33 ppm, and the OH peak had a similar upfield shift of 0.15 ppm. The influence of the CF_3_ group as a strong electron withdrawing group affected the amide signals more significantly. This electron withdrawing property also allows for more enhanced hydrogen bonding from the amide group, which further resulted in more effective gelation in polar organic solvents. 

The ^1^H-NMR spectra of the benzamide **13** were also analyzed at different temperatures, as shown in Figure 7. Like the aliphatic amide, up field shifts for NH (0.22 ppm) and OH (0.15 ppm) were observed for the benzamide **13** from 30–60 °C (Figure S3b). In addition, the benzoyl signals also shifted up field by 0.07 ppm, which indicated that the benzoyl group was important in forming the molecular assemblies and participated in the interactions which resulted in gelation. In addition to the change in chemical shift of the aromatic signals, a small down field shift of 0.03 ppm was observed for the anomeric proton signal at elevated temperatures. 

**Figure 8:**
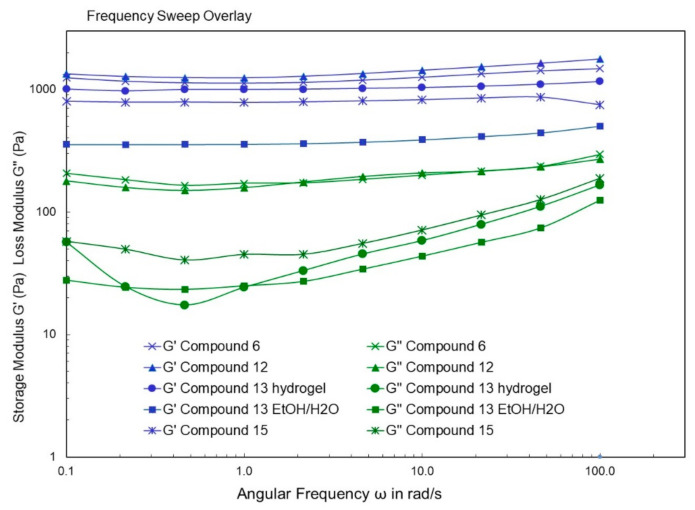
Rheological properties of the gels formed by several gelators at their MGCs: compound **6** in EtOH/H_2_O (1:1) at 0.74 mg/mL; compound **12** in EtOH/H_2_O (1:1) at 0.67 mg/mL, compound **13** in water at 0.36 mg/mL and in EtOH/H_2_O (1:2) at 0.36 mg/mL and compound **15** in DMSO/H_2_O (1:1) at 0.71 mg/mL. All frequency sweeps were using 0.1% strain.

The mechanical stability of several gels with MGCs below 1.0 mg/mL were evaluated at their MGCs using a rheometer. These include gels formed by compounds **6**, **7**, **8**, **12**, **13**, and **15**. Figure 8 displays the stacked frequency sweeps for five selected gels. Individual frequency sweeps, their accompanying amplitude sweeps and rheological data tables are included in Figure S4-1 to S4-8 and Table S1a–i. The storage moduli G′s for all gels are larger than the loss moduli G″s at their minimum gelation concentrations. Although some gels exhibited a similar storage modulus at low concentrations, different loss modulus were observed. The G′/G″ values are greater than 1 for all gels, indicating the mechanical stability and viscoelasticity for these gels. 

The formation of supramolecular gels at very low concentrations is a useful feature for exploring various applications for these gelators. In this study we selected to explore the following three different applications for the gelators, (1) 3-D printing materials, (2) sustained release drug delivery, (3) dye absorption and removal.

**Figure 9:**
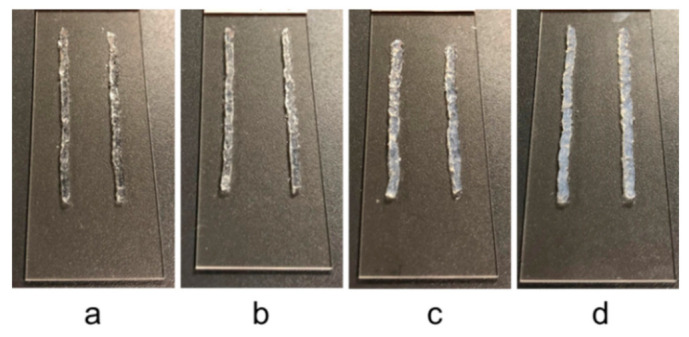
Extruded gels formed by compounds **11** (**a**,**b**) and **13** (**c**,**d**) in DMSO/H_2_O (1:1). The concentrations of the gels are 5.0 mg/mL for (**a**,**c**); 10.0 mg/mL for (**b**,**d**).

**Figure 10:**
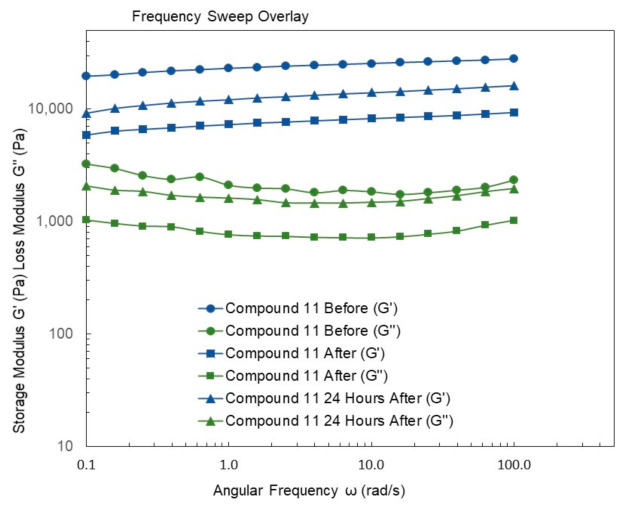
Rheology properties (frequency sweep, 0.5% strain) of a gel of compound **11** in DMSO/H_2_O 1:1 at 5 mg/mL before and after extrusion, and 24 h after extrusion.

**Figure 11:**
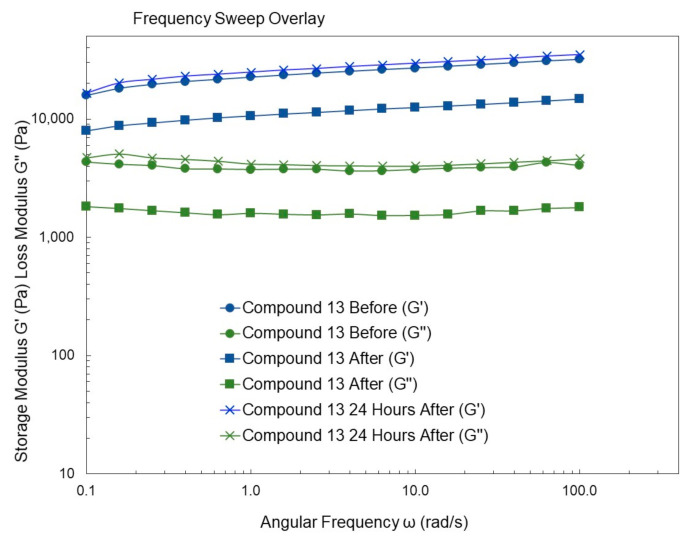
Rheology properties (frequency sweep, 0.5% strain) of a gel of compound **13** in DMSO/H_2_O 1:1 at 5 mg/mL before and after extrusion, and 24 h after extrusion.

Shaping or printing supramolecular gels is typically difficult due to the fragility of their supramolecular scaffolds. Most often, gelation is triggered within a mold, limiting the shaping of supramolecular gels [1]. We set out to analyze shaping our gels through extrusion-based methods, by forming gels in a syringe and using a syringe pump to control the rate of extrusion (Figure S5-1). To form the gels, hot gelator solutions were drawn into 1 mL syringes equipped with a blunt tipped needle. While most gels formed too quickly to be pulled up into a syringe, DMSO/H_2_O gels formed by compounds **11** and **13** were successfully formed within the syringe. Microscope slides were used as print beds. The gel concentrations were found to be important for successful extrusion. At higher concentrations (above 10 mg/mL), solid like clumps were extruded. The rate of the syringe pump was limited to 3.0 mL/min due to the 1 mL syringe size and the most successful extrusions occurred at this maximum extrusion rate. Gels formed by compounds **11** and **13** in DMSO/H_2_O 1:1 at 5 mg/mL and 10 mg/mL and DMSO/H_2_O 1:2 at 5 mg/mL were successfully extruded, producing lines of gels on microscope slides. Several examples are shown in Figure 9 and Figure S5-2, additional optical images of the gels before and after extrusions are included in Figure S5-3. Rheological measurements were carried out on these gel samples before, immediately after, and 24 h after the extrusion experiments to assess the effects that extrusion had on the strength and elasticity of gels. Rheology data for gels formed in DMSO/H_2_O 1:1 at 5 mg/mL are shown in Figure 10 and Figure 11. The amplitude sweeps and rheological data are included in Figures S6 and S7 and Tables S1 and S2. In all studies, the extruded gels were found to be stable as the storage modulus (G′) remained higher than the loss modulus (G″) at a 0.5% strain during the frequency sweeps. When comparing the studies before and after extrusion, a trend was observed that both the storage modulus (G′) and loss modulus (G″) decreased immediately after extrusion, followed by an increase in storage modulus (G′) and loss modulus (G″) 24 h later. While the results of these studies are promising, much more extensive studies are required to analyze the ability of these gels to be used as soft materials for 3-D printing.

**Figure 12:**
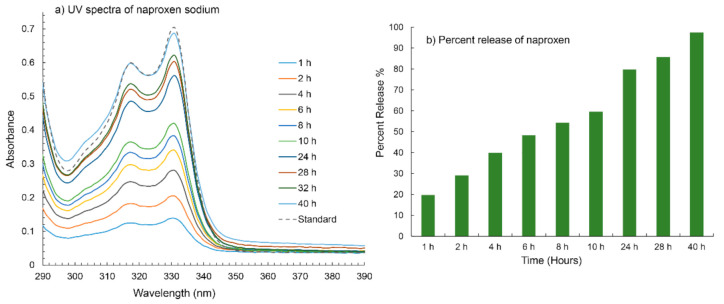
The UV-Vis spectra (**a**) and percent release profile (**b**) of naproxen sodium over time from a co-gel formed by compound **13** (1.0 mg/mL gel) in DMSO:H_2_O (5:95). The gel was prepared using 2 mg of compound **13** and 0.5 mg of naproxen sodium in 2.0 mL of DMSO:H_2_O (5:95) solution. Percent release was calculated using absorption values at 331 nm for each time point.

The benzamide gelator **13** formed a hydrogel at a very low MGC of 0.36 mg/mL, making it a potential candidate for biomedical applications. To analyze potential applications in sustained drug release, drug entrapment and release studies were carried out using model drugs chloramphenicol and naproxen sodium. Gels formed by compound **13** in DMSO/H_2_O (*v*/*v* 5:95) were successfully utilized for the extended release of both model drugs. The 5% DMSO was used for easy sample preparation. Figure 12 shows the UV-vis spectra of the naproxen release to an aqueous phase from a 1 mg/mL gel and the percent release over 40 h. 

About 97% of the naproxen was found to have diffused into the aqueous phase by 40 h. An additional study using a 0.5 mg/mL gel yielded similar results, shown in Figures S10 and S11a, with about 95% of the naproxen sodium being released at 40 h. Figure S11b shows the comparison of the estimated naproxen release from the two gels at different times. The release studies utilizing chloramphenicol as a model drug also demonstrated the gels’ capabilities of sustained drug release, Figure S8A,B. At the end of these studies, the gels were found to be stable enough to be inverted, shown in Figures S9a and S11a. The chloramphenicol release using the two different gels are shown in Figure S9b, which shows that the concentrations of the gels did not affect the rate of diffusion. The UV spectra of gelator **13** are shown in Figure S12. There is some overlap with chloramphenicol but not for naproxen, however, the amount of gelator **13** that diffused into the aqueous phase would be negligible due to its low solubility in water and because the gels remained intact at the end of the experiment. The results of these studies indicate that these gels have potential applications in sustained release drug delivery. 

**Figure 13:**
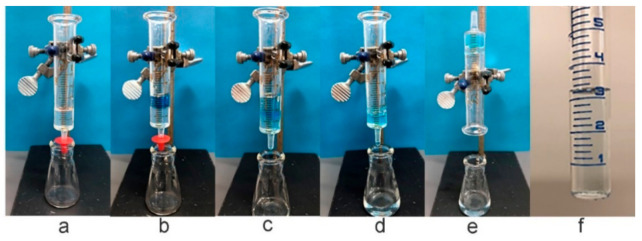
Gel column for the dye absorption experiment. (**a**) Gel column before the experiment; (**b**) Gel column after loading with 1 mL of 32.7 µM TBO stock solution; (**c**) Washing the column with 1 mL of DI water; (**d**) Gel column at the end of the experiment; (**e**) Inverted gel column after the completion of the experiment; (**f**) Very light blue aqueous solution collected from the gel column.

**Figure 14:**
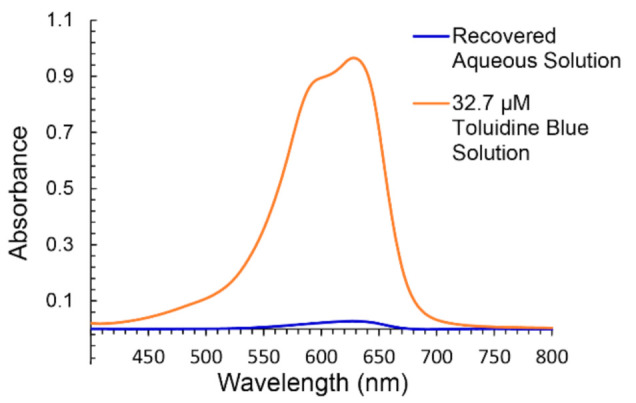
The UV-Vis spectra of the 32.7 µM TBO solution loaded to the top of the gel in Figure 13b and the liquid collected from the gel column (Figure 13f). The TBO was almost fully absorbed by the gel.

The gels formed by compound **13** in DMSO/H_2_O 1:1 were evaluated for the absorption of toluidine blue (TBO) dye. These results are shown in Figure 13 and Figure 14. Gel columns were utilized to remove TBO from a solution that eluted through it. The collected aqueous phase is shown in Figure 13f and the now blue gel was stable enough to be inverted at the end of the experiment (Figure 13e). The UV-Vis spectra of the TBO solution and the collected aqueous solution are shown in Figure 14. The UV-vis of the aqueous solution shows low absorbance at the λ_max_ (630 nm) of TBO, indicating very low presence of TBO. Using a calibration curve of the TBO dye solution, shown in the Figures S13 and S14, and accounting for dilution, we estimate that the gel column removed about 89% of the TBO dye from the initial stock solution, leaving less than 11% of the TBO dye in the collected aqueous solution. Additional images of the dye absorption experiment are shown in Figure S15. 

This result is significant since it indicates that the gel, even at a very low concentration, is effective in removing toxic dyes from water. With the success of the first gel column removing 89% of TBO from an aqueous solution, an additional experiment was carried out using a 4 mL gel formed by compound **13** in EtOH/H_2_O (1:2) using a similar procedure. This gel was also found to remove a majority of the dye from the TBO solution. 

The results for this study can be found in Figures S16 and S17. Further studies exploring the uses of these gelators for environmental cleanup are ongoing and will be reported in due course. 

## 3. Conclusions

A series of fifteen amide derivatives of 4,6-*O*-(*p*-chlorobenzylidene) acetal-protected d-glucosamine were designed and synthesized. All derivatives showed remarkable gelation properties in the selected series of solvents, with several compounds forming gels at very low MGCs. The introduction of a chloro substituent to the phenyl ring of the 4,6-benzylidene acetal has resulted in very effective gelators, with the benzamide **13** forming gels in water at 0.04 wt% and several other gelators forming gels at less than 0.1 wt%. Optical imaging and AFM studies showed that the gelators formed self-assembled fibrous networks. Analysis on the molecular shapes and 3-D volumes of different compounds revealed structure to gelation properties correlations, which can possibly be used to predict the performance of other analogs. Replacing the methyl groups in the t-butyl group with chlorine or fluorine atoms results in much more effective LMWGs. The effect produced from these small structural modifications on these glucosamine derivatives indicates that halogen bonding can be an important contributing factor towards supramolecular gelation. The ^1^H-NMR spectroscopy data acquired at different temperatures indicated that the amide and 3-hydroxyl hydrogen bonds are important for molecular self-assemblies. The aromatic π-π interaction, as well as CH-π interaction and halogen bonding are also important in the design of effective LMWGs. Rheological properties of several super gelators at their MGCs demonstrated their viscoelastic properties and mechanical stability. In addition, the gels formed by compounds **11** and **13** showed strong stability and can be extruded onto glass slides for pattern formation. The gels formed by gelator **13** were able to entrap naproxen and chloramphenicol and showed sustained release properties over time. Gelator **13** was very effective at removing toluidine blue dye from aqueous solution, with almost 89% of the dye being adsorbed on to a gel column. We anticipate broad utilities of the gelators discovered in this study over various research fields. This structure-based design to fine-tune supramolecular gel properties can be applied in the rational design of other supramolecular gelators. 

## 4. Materials and Methods

### 4.1. General Methods and Materials

All solvents and reagents were acquired from chemical suppliers. All purifications were carried out through column chromatography using 230–400 mesh silica gel with gradient solvent systems or recrystallizations in ethanol. NMR analysis was carried our utilizing an AVANCE III 400 MHz NMR spectrometer (Bruker, Billerica, MA, and processed using TopSpin 4.0.7. Melting points were acquired using a Fisher-Johns melting point apparatus (Thermo Scientific, Waltham, MA, USA). UV-vis spectra were obtained using an Evolution 210 UV-Visible spectrophotometer (Thermo Scientific, Waltham, MA, USA). Mass spectroscopy was carried out using LC-MS on an Agilent 6120B single quad mass spectrometer and a LC1260 liquid chromatography system (Agilent Technologies, Inc., Santa Clara, CA, USA). 

### 4.2. Optical Microscopy

Images of the gels’ morphology were captured using an BX60M optical microscope (Olympus, Tokyo, Japan) using an Olympus DP73-1-51 (Olympus, Tokyo, Japan) high-performance 17MP digital camera with pixel shifting and Peltier cooling. CellSens Dimension 1.11 was used to acquire and store the images. Typically, gel samples were transferred to a clean microscope slide using a pipette or spatula and allowed to dry for one day before imaging. An alternative method was used for image in Figure 4h, an aliquot of the gel was placed on cellulose frits and vacuum filtration was utilized to remove excess solvent, leaving the gel’s microstructures on the frit. These were then transferred to a glass slide using razor blade and observed immediately. 

### 4.3. Atomic Force Microscopy

Images of the gels’ morphology were captured using a Dimension 3100 atomic force microscope (Veeco, Plainview, NY, USA). Tap300-G silicon AFM probes (Nanosensors, Neuchatel, Switzerland) with a resonant frequency of 300 KHz and a force constant of 40 N/m were utilized for imaging. All images were obtained in tapping mode. The sample preparation for AFM imaging was the same as used for optical microscopy imaging. 

### 4.4. Gelation Tests 

Two mg of compound was weighed out in a one dram vial and 0.1 mL of the desired solvent was added, giving a starting concentration of 20.0 mg/mL. The mixture was then heated until the gelator dissolved. Upon cooling to rt (about 30 min), the sample was examined. For samples that formed a gel, the gel vials were inverted and gently tapped. If no solvent flowed when the vial was inverted, the sample was recorded as a stable gel. If solvent movement was observed, the sample was recorded as an unstable gel. If the sample formed a gel, another 0.1 mL of the solvent was added, and the method was repeated. This method was repeated until an unstable gel was formed. Minimum gelation concentrations (MGCs) were recorded as the concentration one addition before the formation of an unstable gel. Several gelators would not dissolve fully at lower concentrations and required the addition of up to 0.6 mL of the solvent to dissolve when heated. Several compounds also required a 2-dram vial for the gelation test. The appearances of the gels are defined as follows: clear, the gel is transparent; opaque, the gel appeared like a white solid and doesn’t allow any light to pass through; translucent, the gel is semi-transparent and can allow some light to pass through. 

### 4.5. Naproxen Trapping and Release Studies

A stock solution of naproxen sodium was created by dissolving 25 mg of naproxen sodium in 100 mL of a 5% DMSO/DI water solution. This solution was then used to prepare 2 mL gels and the gels were left at room temperature for ~12 h. 2 mL of water (pH 7) was placed on top of the gel. At specific time intervals, the water was carefully removed and transferred to a cuvette to record UV absorbance. The aqueous layer was returned to the gel vial after analysis. Two diffusion studies were carried out using compound **13** to analyze the effect of the gelator concentration on the diffusion rate. Two 2 mL gels were created by dissolving 2 mg and 1 mg of the gelator in 2 mL of the naproxen stock solution. The gelator concentrations were 1.0 mg/mL and 0.5 mg/mL. The initial naproxen sodium concentration was 0.25 mg/mL. 

### 4.6. Chloramphenicol Trapping and Release Studies

A similar method for naproxen release described was used. A stock solution of chloramphenicol was created by dissolving 20 mg of chloramphenicol in 100 mL of a 5% DMSO/DI water solution. This solution was then used to prepare 2 mL gels. After 12 h at rt, 2 mL of water at pH 7 was placed on top of the gel. At specific time intervals, the water was carefully removed and transferred to a cuvette to record UV absorbance. The aqueous layer was returned to the gel vial after analysis. Two experiments were carried out using gelator **13** at 1.0 mg/mL and 0.5 mg/mL concentrations. The initial chloramphenicol concentration was 0.20 mg/mL for both experiments. 

### 4.7. Dye Absorption Studies

The gel column was prepared using 2 mL of a gel formed by compound **13** (0.5 mg/mL in DMSO/H_2_O 1:1). The sample was prepared by using 1.0 mg (2.4 µmol) compound **13** and 2 mL of DMSO/H_2_O 1:1 solution in a 2 dram vial. The mixture was then heated until the gelator fully dissolved. While still hot, the solution was poured into a plugged syringe. After cooling, a clear gel formed. Then 1 mL of a 32.7 µM toluidine blue solution was added to the top of the gel and the plug was removed to allow the solution to elute. After elution was complete, the column was then flushed with 1 mL of DI water, twice. A total of 3.2 mL of faint blue solution was collected from the column. UV-vis spectroscopy was carried out on the collected aqueous phase and a calibration curve was utilized to calculate TBO concentration. The concentration of TBO in the collected solution was 1.119 µM, and the total amount of TBO in 3.2 mL of solution was 0.001095 mg. The gel column was calculated to have removed 89.05% of the toluidine blue from solution. Another gel of compound **13** in EtOH/H_2_O (1:2) was also studied using a similar method. The gel column was prepared using 4 mL of a 0.5 mg/mL gel formed by compound **13** in EtOH/H_2_O (1:2). The same dye solution was added to the gel column. After elution was complete the column was then flushed with 3 mL of DI water. A total of 5.2 mL of solution was collected from the column. The TBO concentration of the solution was 1.344 µM and the total amount of TBO was 0.00214 mg. Overall, the gel column efficiently removed 78.6% of the dye from the original solution. 

### 4.8. Extrusion Studies

Gels were prepared in a 1 dram vial using DMSO/H_2_O solution. The vial was then sealed and gently heated until the gelator dissolved. While still hot, the solution was drawn up into a 1 mL syringe. Upon cooling, the gel formed inside the syringe. The syringe was set upright for 24 h for the gel to settle. Extrusion studies were carried out using a Fusion 100 Touch syringe pump (Chemyx, New Castle, DE, USA). The syringes containing the gelator sample were equipped with a 14-gauge blunt tipped needle and placed in the syringe pump vertically. The extrusion rate was 3 mL/min. Using a guide, a microscope slide was placed underneath the syringe and slid under the pump during extrusion. The clearance between the tip of the blunt tipped needle and the microscope slide is ≈2.2 mm, measured via a micrometer. For each trial, 200 µL of gel were extruded over a distance of 4 cm.

### 4.9. Rheological Studies

The gel extrusion studies were carried out on a Discovery HR2 Hybrid Rheometer from TA Instruments (TA Instruments, New Castle, DE, USA) equipped with the TRIOS software. The cone geometry is 25-mm Peltier plate and with a gap of 100 µm. The samples were analyzed before and after the extrusion of the gels and after 24 h after extrusion. The linear viscosity range was obtained via amplitude sweeps, which were carried out using an angular frequency (ω) of 10.0 radians per second. Frequency sweeps were then carried out using a strain of 0.5%. One of the 200 µL lines was immediately scraped off the glass and centered in a pile under cone plate. The second line was placed in an airtight box to prevent solvent evaporation, until the second rheological experiment was carried out 24 h later. In each study, 200 µL of the hot gelator solution was left in the original vial and set aside to form a gel to be used as the non-extruded sample. This entire gel sample was carefully removed for the “before extrusion” studies. 

For the gels formed by the super gelators, the rheology was done using a MCR 302 rheometer (Anton Parr, Graz, Austria) equipped with RheoCompass software. The same cone geometry was used and the sample preparation and methods used are similar to what was described above. The strain used for the frequency sweep was 0.1%. 

### 4.10. Synthesis and Characterization Data for Compounds ***3**–**18***

#### 4.10.1. Synthesis of Compound **3**

4-Chlorobenzaldehyde (5.60 g, 25.6 mmol, 1.2 equiv), trimethyl orthoformate (5.2 mL, 47.9 mmol, 2.25 equiv) and PTSA (0.418 g, 2.2 mmol, 1.0 equiv) were added to a 100 mL nitrogen flushed round bottom flask containing anhydrous MeOH (5 mL). The reaction mixture was refluxed for 3 h. The solvent was then removed under reduced pressure and the crude product was dissolved in anhydrous DMF (10 mL). Compound **2** (5.00 g, 21.3 mmol, 1 equiv) was then added and the reaction mixture was heated to 70 °C. The reaction was stirred at 70 °C for 6 h. Upon cooling the reaction mixture, a white solid precipitated. The reaction mixture was diluted with DCM (30 mL) and the precipitate was filtered. The filtrate was quenched with NaHCO_3_, followed by an aqueous workup using DI water (×3) and DCM (×2). Organic layers were dried with Na_2_SO_4_ and the solvent was removed under reduced pressure leaving an off white solid. The precipitate and crude product were combined and recrystallized in ethanol. The mother liquor was then purified via column chromatography (SiO_2_) using 0–5% MeOH/DCM affording a white solid (5.974 g, 78%). R_f_ = 0.36 in 3% MeOH/DCM, mp 273.2–275.2 °C. ^1^H-NMR (400 MHz, d_6_-DMSO) *δ* 7.88 (d, *J* = 8.3 Hz, 1H); 7.51–7.42 (m, 4H); 5.63 (s, 1H); 5.15 (d, *J* = 5.7 Hz, 1H); 4.62 (d, *J* = 3.4 Hz, 1H); 4.22–4.14 (m, 1H); 3.89–3.80 (m, 1H); 3.74 (t, *J* = 10.1, 1H,); 3.70–3.55 (m, 2H), 3.53–3.45 (m, 1H), 3.29 (s, 3H); 1.85 (s, 3H,). ^13^C-NMR (100 MHz, d_6_-DMSO) *δ* 169.5; 136.6, 133.4, 128.2, 128.1; 99.9; 98.7; 82.0; 68.0; 67.3; 62.3; 54.7; 54.1; 22.5. MS *m*/*z* calculated for C_16 H20_ClNO_6_Na [M+Na]^+^ 380.1 found 380.1.

#### 4.10.2. Synthesis of Compound **4**

Compound **3** (5.974 g, 16.7 mmol, 1 equiv.) was added to a 250 mL round bottomed flask containing a 1N NaOH ethanol solution. The reaction mixture was heated to refluxing temperature for 48 h. The reaction was monitored via ^1^H-NMR and thin layer chromatography (TLC). Ethanol was removed under reduced pressure leaving an off white solid. An aqueous workup was carried out using EtOAc (×3) and DI water (×3). Organic layers were dried with Na_2_SO_4_ and the solvent was removed under reduced pressure. The crude product was purified via column chromatography (SiO_2_) using 0–15% MeOH/DCM to afford a white solid (5.0126 g, 95%). R_f_ = 0.18 in 3% MeOH/DCM, mp 207.9–209.1 °C. ^1^H-NMR (400 MHz, CDCl_3_) *δ* 7.46–7.40 (m, 2H); 7.36–7.31 (m, 2H); 5.50 (s, 1H); 4.67 (d, *J* = 3.6 Hz, 1H); 4.29–4.22 (m, 1H); 3.82–3.66 (m, 3H); 3.45 (t, *J* = 9.1 Hz, 1H); 3.40 (s, 3H); 2.81–2.74 (m, 1H). ^13^C-NMR (100 MHz, CDCl_3_) *δ* 135.8; 135.0; 128.5; 127.8; 101.2; 101.0; 82.0; 71.5; 69.1; 62.5; 56.7; 55.5. LC-MS *m*/*z* calculated for C_14 H19_ClNO_5_ [M+H]^+^ 316.1 found 316.1.

#### 4.10.3. General Procedure for the Synthesis of Amides **5**–**18**

The amides were synthesized using the corresponding acid chlorides (for compounds **6**–**10** and **13**–**18**) or anhydrides (for compounds **11**–**12**). In general, the headgroup amine **4** (1 equiv.) was added to a round bottom flask with a drying tube under nitrogen atmosphere, followed by anhydrous DCM and either pyridine (5 equiv) or triethylamine (3 equiv), the flask was cooled to 0 °C, and the acid chloride or anhydride (1.1 equiv) diluted in anhydrous DCM, was added dropwise. Reaction mixture was typically stirred at 0 °C for 1–2 h. Reactions were monitored via ^1^H-NMR spectroscopy and TLC. After completion, the reaction mixture was then quenched with 5% NaHCO_3_ and stirred for an additional 30 min. Reaction mixture then underwent an aqueous workup with saturated NaHCO_3_, saturated NH_4_Cl, DI water, and DCM (×3). The combined organic phase was dried with Na_2_SO_4_ and solvent was removed under reduced pressure. The crude product was purified via flash column chromatography on silica gel using a gradient solvent system. The detailed preparation for compound **5** is provided below and only amount used and characterization data are provided for all other compounds. All compounds were synthesized using 75 mg (0.24 mmol) of compound **4** and 0.1 mL of either pyridine or trimethylamine unless otherwise mentioned. 

#### 4.10.4. General Procedure for the Synthesis of Acid Chlorides 

Corresponding acids (1 equiv) were dissolved in anhydrous DCM with 1 drop of DMF in nitrogen flushed 50 mL round bottomed flasks. The temperature was reduced to 0 °C via an ice bath and oxalyl chloride (1.2 equiv) was added to the reaction mixture. The reaction mixture was stirred at 0 °C for 10 min, at which point the ice bath was removed. The reaction mixtures continued to stir for an additional 3 h at rt. Conversion to the acid chloride was monitored via ^1^H-NMR The crude product was used directly into the amide synthesis.

#### 4.10.5. Synthesis of Compound **5**

Compound **4** (0.075 g, 0.24 mmol, 1 equiv) dissolved in 3 mL of anhydrous DCM and anhydrous pyridine (0.1 mL, 1.2 mmol) was added to a dried 50 mL round bottomed flask. The reaction mixture was cooled in an ice bath. The propionyl chloride, prepared from propionic acid (37 µL, 0.27 mmol, 1.1 equiv) in situ, was dissolved in 2 mL anhydrous DCM and added dropwise to the flask over 10 min. The reaction mixture was stirred at 0 °C for 1 h. Reaction conversion was monitored via ^1^H-NMR and TLC. The crude product was purified via column chromatography using DCM to 5% MeOH/DCM to afford a white solid (84 mg, 94%) as the desired product. R_f_ = 0.11 in 1% MeOH/DCM, mp 258.9–261.3 °C. ^1^H-NMR (400 MHz, CDCl_3_) *δ* 7.47–7.41 (m, 2H); 7.36–7.30 (m, 2H); 5.85 (d, *J* = 8.6, 1H); 5.54 (s, 1H); 4.71 (d, *J* = 3.8 Hz, 1H); 4.33–4.17 (m, 2H); 3.89 (t, *J* = 9.6 Hz, 1H); 3.81–3.72 (m, 2H); 3.58 (t, *J* = 8.8, 1H); 3.41 (s, 3H); 2.30 (q, *J* = 7.5, 2H); 1.18 (t, *J* =7.6, 3H). ^13^C-NMR (100 MHz, CDCl_3_) *δ* 175.5; 135.7; 135.0; 128.4; 127.8; 101.1; 98.8; 82.1; 71.1; 68.8; 62.2; 55.3; 54.1; 29.6; 9.6. LC-MS *m*/*z* calculated for C_17 H23_ClNO_6_ [M+H]^+^ 372.1 found 372.2.

#### 4.10.6. Synthesis of Compound **6**

Valeryl chloride (0.27 mmol, 1.1 equiv) was synthesized from valeric acid (30 µL, 0.27 mmol) and oxalyl chloride (25 µL, 0.29 mmol). The crude acid chloride, compound **4**, pyridine, and DCM were used. The reaction mixture was stirred at 0 °C for 2 h. Crude product was purified via trituration using 50% EtOAc/hexanes, which afforded a white solid (89 mg, 82%). R_f_ = 0.17 in 1% MeOH/DCM, mp 238.7–240.0 °C. ^1^H-NMR (400 MHz, CDCl_3_) *δ* 7.47–7.40 (m, 2H); 7.36–7.30 (m, 2H); 5.87 (d, *J* = 8.4); 5.53 (s, 1H); 4.71 (d, *J* = 3.9 Hz, 1H); 4.34–4.15 (m, 2H); 3.87 (t, *J* = 9.6 Hz, 1H); 3.81–3.71 (m, 2H); 3.62–3.54 (m, 1H); 3.40 (s, 3H); 2.26 (t, *J* = 7.6, 2H); 1.69–1.58 (m, 2H); 1.43–1.30 (m, 2H); 0.92 (t, *J* = 7.3, 3H). ^13^C- NMR (100 MHz, CDCl_3_) *δ* 174.9; 135.7; 135.0; 128.4; 127.8; 101.1; 98.8; 82.1; 71.0; 68.8; 62.2; 55.3; 54.1; 36.4; 27.6; 22.3; 13.7. LC-MS *m*/*z* calculated for C_19_H_26_ClNO_6_Na [M+Na]^+^ 422.1 found 422.1.

#### 4.10.7. Synthesis of Compound **7**

Hexanoyl chloride (38 μL, 0.27 mmol, 1.1 equiv), compound **4**, pyridine, and DCM were used. The reaction mixture was stirred at 0 °C for 2 h. The crude product was purified via chromatography using 0–5% MeOH/DCM, affording a white solid (90 mg, 91%). R_f_ = 0.17 in 1% MeOH/DCM, mp 228.3–229.8 °C. ^1^H-NMR (400 MHz, CDCl_3_) *δ* 7.47–7.40 (m, 2H); 7.37–7.30 (m, 2H); 5.85 (d, *J* = 8.1, 1H); 5.54 (s, 1H); 4.71 (d, *J* = 3.7 Hz, 1H); 4.36–4.16 (m, 2H); 3.89 (t, *J* = 9.5 Hz, 1H); 3.82–3.71 (m, 2H); 3.63–3.54 (m, 1H); 3.41 (s, 3H); 2.25 (t, *J* = 7.5, 2H); 1.71–1.59 (m, 2H); 1.41–1.24 (m, 4H,); 0.90 (t, *J* = 6.9, 3H). ^13^C-NMR (100 MHz, CDCl_3_) *δ* 174.9; 135.7; 135.0; 128.4; 127.8; 101.1; 98.8; 82.1; 71.1; 68.8; 62.2; 55.3; 54.1; 36.1; 31.3; 25.3; 22.3; 13.9.LC-MS *m*/*z* calculated for C_20_H_29_ClNO_6_ [M+H]^+^ 414.2 found 414.3 and [M+Na]^+^ 436.2 found 436.3.

#### 4.10.8. Synthesis of Compound **8**

Heptanoyl chloride (42 μL, 0.27 mmol, 1.1 equiv) was dissolved in anhydrous DCM and added dropwise to a flask containing compound **4**, pyridine, and DCM. The reaction mixture was stirred at 0 °C for 2 h. The crude product was purified via column chromatography using 0–5% MeOH/DCM, affording a slightly yellow solid (91 mg, 88%). R_f_ = 0.29 in 1% MeOH/DCM, mp 223.9–225.8 °C, ^1^H-NMR (400 MHz, CDCl_3_) *δ* 7.48–7.40 (m, 2H); 7.37–7.30 (m, 2H); 5.85 (d, *J* = 8.2, 1H); 5.54 (s, 1H); 4.71 (d, *J* = 3.8 Hz, 1H); 4.35–4.15 (m, 2H); 3.88 (t, *J* = 9.6 Hz, 1H); 3.82–3.70 (m, 2H); 3.58 (t, *J* = 8.7, 1H); 3.40 (s, 3H); 2.25 (t, *J* = 7.5, 2H); 1.73–1.56 (m, 2H); 1.42–1.15 (m, 4H); 0.98–0.79 (m, 3H). ^13^C-NMR (100 MHz, CDCl_3_) *δ* 174.9; 135.7; 135.0; 128.4; 127.8; 101.1; 98.8; 82.1; 71.1; 68.8; 62.2; 55.3; 54.1; 36.6; 31.5; 29.7; 28.8; 25.5; 22.5; 14.0. LC-MS *m*/*z* calculated for C_21 H31_ClNO_6_ [M+H]^+^ 428.2 found 428.2.

#### 4.10.9. Synthesis of Compound **9**

Cyclohexanecarbonyl chloride (0.27 mmol, 1.1 equiv) was synthesized using cyclohexanecarboxylic acid (34 mg, 0.27 mmol, 1.1 equiv) and oxalyl chloride (25 µL, 0.29 mmol, 1.2 equiv). The crude acid chloride, compound **4**, pyridine, and DCM were used. The reaction mixture was stirred at 0 °C for 1 h. The crude product was purified via column chromatography using 0–5% MeOH/DCM, affording an off white solid (88 mg, 87%). R_f_ = 0.23 in 1% MeOH/DCM, mp 250.3–253.2 °C. ^1^H-NMR (400 MHz, CDCl_3_) *δ* 7.47–7.40 (m, 2H); 7.36–7.30 (m, 2H); 5.89 (d, *J* = 8.4, 1H); 5.54 (s, 1H); 4.70 (d, *J* = 3.9 Hz, 1H); 4.32–4.16 (m, 2H); 3.88 (t, *J* = 9.6 Hz, 1H); 3.81–3.72 (m, 2H); 3.58 (t, *J* = 9.1, 1H); 3.41 (s, 3H); *δ* 2.16 (tt, *J* = 11.6, *J* = 3.5, 1H); 1.98–1.73 (m, 4H); 1.72–1.61 (m, 1H,); 1.53–1.39 (m, 2H); 1.35–1.16 (m, 3H). ^13^C-NMR 100 MHz, CDCl_3_) *δ* 178.0; 135.7; 135.0; 128.4; 127.8; 101.0; 98.8; 82.1; 71.3; 68.8; 62.2; 55.3; 54.1; 45.3; 29.6; 29.5; 25.65; 25.62; 25.60. LC-MS *m/z* calculated for C_21 H29_ClNO_6_ [M+H]^+^ 426.1 found 426.1.

#### 4.10.10. Synthesis of Compound **10**

Pivaloyl chloride (33 μL, 0.27 mmol, 1.1 equiv), compound **4**, pyridine, and DCM were used. The reaction mixture was stirred at 0 °C for 2 h. The crude product was purified via column chromatography using 0–3% MeOH/DCM, affording a white solid (70 mg, 73%). R_f_ = 0.23 in 1% MeOH/DCM, mp 162.4–164.3 °C. ^1^H-NMR (400 MHz, CDCl_3_) *δ* 7.47–7.41 (m, 2H); 7.36–7.30 (m, 2H); 6.08 (d, *J* = 8.1, 1H); 5.54 (s, 1H); 4.70 (d, *J* = 3.9 Hz, 1H); 4.35–4.14 (m, 2H); 3.89 (t, *J* = 9.5 Hz, 1H); 3.83–3.70 (m, 2H); 3.62–3.55 (m, 1H); 3.41 (s, 3H,); 1.23 (s, 9H). ^13^C-NMR (100 MHz, CDCl_3_) *δ* 180.7; 135.7; 134.9; 128.4; 127.8; 101.0; 98.8; 82.1; 71.5; 68.8; 62.2; 55.4; 54.2; 38.8; 27.5. LC-MS *m/z* calculated for C_19_H_27_ClNO_6_ [M+H]^+^ 400.1 found 400.1 and [M+Na]^+^ 422.1 found 422.1.

#### 4.10.11. Synthesis of Compound **11**

Trifluoroacetic anhydride (100 μL, 0.71 mmol, 1.1 equiv) was dissolved in anhydrous DCM and added dropwise to a flask containing compound **4** (200 mg, 0.64 mmol, 1 equiv), and trimethylamine (0.450 mL, 3.2 mmol). The reaction mixture was stirred at 0 °C for 1.5 h. The crude product was purified via column chromatography using 0–3% MeOH/DCM, affording a white solid (244 mg, 93%). R_f_ = 0.31 in 1% MeOH/DCM, mp 291.3–292.7 °C, sample turned brown at 285.2 °C ^1^H-NMR (400 MHz, d_6_-DMSO) *δ* 9.56 (d, *J* = 7.1 Hz, 1H); 7.51–7.40 (m, 4H); 5.65 (s, 1H); 5.37 (d, *J* = 5.5 Hz, 1H; 4.74 (d, *J* = 3.1 Hz, 1H); 4.24–4.16 (m, 1H); 3.96–3.83 (m, 2H); 3.76 (t, *J* = 10.1, 1H); 3.68–3.59 (m, 1H), 3.57–3.49 (m, 1H), 3.32 (s, 3H). ^13^C-NMR (100 MHz, d_6_-DMSO) *δ* 157.2; 156.8; 156.5; 156.1; 136.6; 134.5; 128.2; 128.1; 120.1; 117.3; 114.4; 111.7; 99.9; 97.7; 81.7; 67.8; 66.4; 62.3; 55.1; *δ* 54.9. LC-MS *m*/*z* calculated for C_16 H17_ClF_3_NO_6_Na [M+Na]^+^ 434.1 found 434.1.

#### 4.10.12. Synthesis of Compound **12**

Trichloroacetic anhydride (59 μL, 0.32 mmol, 1.3 equiv) was dissolved anhydrous DCM and added to a flask containing compound **4**, trimethylamine (0.100 mL, 0.72 mmol) and DCM. The reaction mixture was stirred at 0 °C for 7.5 h. The crude product was purified via column chromatography using 0–3% MeOH/DCM, affording a white solid (86 mg, 87%). R_f_ = 0.40 in 1% MeOH/DCM. mp 181.7–182.8 °C. ^1^H-NMR (400 MHz, CDCl_3_) *δ* 7.46–7.40 (m, 2H); 7.38–7.32 (m, 2H); 6.91 (d, *J* = 8.7, 1H); 5.55 (s, 1H); 4.83 (d, *J* = 3.7 Hz, 1H); 4.34–4.26 (m, 1H); 4.22–4.14 (m, 1H); 4.02 (t, *J* = 9.6, 1H); 3.86–3.74 (m, 2H); 3.60 (t, *J* = 9.1, 1H); 3.45 (s, 3H). ^13^C-NMR (100 MHz, CDCl_3_) *δ* 162.5; 135.5; 135.2; 128.5; 127.7; 101.1; 98.3; 92.4, 81.6; 70.0; 68.7; 62.4; 55.6; 55.5. LC-MS *m*/*z* calculated for C_16 H17_Cl_4_NO_6_Na [M+Na]^+^ 482.1 and 482.1.

#### 4.10.13. Synthesis of Compound **13**

Benzoyl chloride (38 μL, 0.27 mmol, 1.1 equiv), compound **4**, pyridine, and DCM were used. The reaction mixture was stirred at 0 °C for 1 h. The crude product was purified via column chromatography using 0–3% MeOH/DCM, affording a white solid (86 mg, 85%). R_f_ = 0.31 in 1% MeOH/DCM. mp 202.3–203.5 °C ^1^H-NMR (400 MHz, CDCl_3_) *δ* 7.85–7.75 (m, 2H); 7.57–7.50 (m, 1H); 7.49–7.40 (m, 4H); 7.37–7.31 (m, 2H); 6.54 (d, *J* = 8.5, 1H); 5.56 (s, 1H); 4.83 (d, *J* = 3.8 Hz, 1H); 4.48–4.40 (m, 1H); 4.32–4.27 (m, 1H); 4.01 (t, *J* = 9.6 Hz, 1H); 3.87–3.75 (m, 2H); 3.65 (t, *J* = 9.1, 1H); 3.43 (s, 3H). ^13^C-NMR (100 MHz, CDCl_3_) *δ* 168.6; 135.6; 135.0; 133.6; 132.0; 128.7; 128.4; 127.8; 127.2; 101.1; 98.9; 82.1; 71.0; 68.8; 62.3; 55.4; 54.6.LC-MS *m*/*z* calculated for C_21 H23_ClNO_6_ [M+H]^+^ 420.1 found 420.2 and [M+Na]^+^ 442.1 found 442.1.

#### 4.10.14. Synthesis of Compound **14**

4-Bromobenzoyl chloride (59 mg, 0.27 mmol, 1.1 equiv), compound **4**, pyridine, and DCM were used. The reaction mixture was stirred at 0 °C for 1.5 h. The crude product was purified via trituration using 5% MeOH/DCM, affording a white solid (88 mg, 74%). R_f_ = 0.31 in 1% MeOH/DCM. mp 308.0–309.0 °C. ^1^H-NMR (400 MHz, d_6_-DMSO) *δ* 8.27 (d, *J* = 7.8, 1 H); 7.91–7.80 (m, 2H); 7.72–7.63 (m, 2H); 7.53–7.40 (m, 4 H); 5.66 (s, 1H); 5.06 (d, *J* = 5.6, 1H); 4.78 (d, *J* = 3.6 Hz, 1H); 4.25–4.18 (m, 1H); 4.12–4.03 (m, 1H); 3.98–3.89 (m, 1H); 3.83–3.74 (m, 1H); 3.72–3.64 (m, 1H); 3.61–3.54 (m, 1H); 3.16 (s, 3H). ^13^C-NMR (100 MHz, d_6_-DMSO) *δ* 166.5; 136.5; 133.2; 130.9; 129.4; 128.0; 127.8; 124.6; 99.8; 98.4; 81.7; 67.8; 66.9; 62.2; 55.0; 54.7. LC-MS *m*/*z* calculated for C_21 H22_BrClNO_6_ [M+H]^+^ 498.0 and 500.0 found 497.9 and 499.9.

#### 4.10.15. Synthesis of Compound **15**

3-Chlorobenzoyl chloride (0.27 mmol, 1.1 equiv) was synthesized using 3-chlorobenzoic acid (41 mg, 0.27 mmol, 1.1 equiv) and oxalyl chloride (25 µL, 0.29 mmol, 1.2 equiv). The crude acid chloride, compound **4**, pyridine, and DCM were used. The reaction mixture was stirred at 0 °C for 12 h. Crude product was purified via recrystallization in ethanol and the mother liquor was purified via column chromatography using 20–80% EtOAc/hexanes, affording a white solid (91 mg, 84%). R_f_ = 0.26 in 1% MeOH/DCM. mp 225.2–227.0 °C. ^1^H-NMR (400 MHz, CDCl_3_) *δ* 7.83 (t, *J* = 3.4 Hz, 1H); 7.72 (d, *J* = 7.8, 1H); 7.55–7.32 (m, 7H); 6.53 (d, *J* = 8.7 Hz, 1H); 5.59 (s, 1H); 4.87 (d, *J* = 3.8, 1H); 4.50–4.41 (m, 1H); 4.39–4.27 (m, 1H); 4.06 (t, *J* = 9.6 Hz, 1H); 3.91–3.76 (m, 2H); 3.67 (t, *J* = 9.1 Hz); 3.47 (s, 3H). ^13^C NMR (100 MHz, CDCl_3_) *δ* 167.1; 135.6; 135.5; 135.1; 134.9; 132.0; 130.0; 128.5; 127.8; 127.6; 125.3; 101.2; 98.8; 82.0; 70.6; 68.8; 62.4; 55.4; 54.6. LC-MS *m*/*z* calculated for C_21 H22_Cl_2_NO_6_ [M+H]^+^ 454.1 found 454.0.

#### 4.10.16. Synthesis of Compound **16**

1-Naphthoyl chloride (41 µL, 0.27 mmol, 1.1 equiv) was dissolved in anhydrous DCM and added to a flask containing compound **4**, pyridine and DCM. The reaction mixture was stirred at 0 °C for 3 h. Crude reaction mixture was purified via column chromatography using 0–3% MeOH/DCM and trituration with ethanol (94 mg, 84%). R_f_ = 0.26 in 1% MeOH/DCM. mp 227.7–229.0 °C. ^1^H-NMR (400 MHz, CDCl_3_) *δ* 8.38–8.30 (m, 1H); 7.94 (d, *J* = 8.3, 1H); 7.91–7.85 (m, 1H); 7.70–7.64 (m, 1H); 7.59–7.50 (m, 2H); 7.50–7.40 (m, 3H); 7.36–7.30 (m, 2H); 6.40 (d, *J* = 8.8, 1H); 5.54 (s, 1H); 4.92 (d, *J* = 3.8, 1H); 4.59–4.51 (m, 1H); 4.35–4.26 (m, 1H); 4.03 (t, *J* = 9.6 MHz, 1H); 3.86–3.75 (m, 2H); 3.64 (t, *J* = 9.1 Hz, 1H); 3.41 (s, 3H). ^13^C-NMR (100 MHz, CDCl_3_) *δ* 170.7; 135.7; 135.0; 133.73; 133.69; 131.0; 130.1; 128.44; 128.38; 127.8; 127.4; 126.6; 125.3; 124.7; 101.1; 98.9; 82.1; 70.9; 68.8; 62.4; 55.4; 54.6. LC-MS *m*/*z* calculated for C_25 H25_ClNO_6_ [M+H]^+^ 470.1 found 470.1.

#### 4.10.17. Synthesis of Compound **17**

1-Naphthylacetyl chloride (0.27 mmol, 1.1 equiv) was synthesized using 1-naphthaleneacetic acid (50 mg, 0.27 mmol, 1.1 equiv) and oxalyl chloride (25 µL, 0.29 mmol, 1.2 equiv). The crude acid chloride, compound **4**, pyridine, and DCM were used. The reaction mixture was stirred at 0 °C for 3 h. Crude reaction mixture was purified via trituration using 5% MeOH/DCM (79 mg, 71%). R_f_ = 0.14 in 1% MeOH/DCM. mp 258.4–260.9 °C. ^1^H-NMR (400 MHz, DMSO-d_6_) *δ* 8.33 (d, *J* = 8.2 Hz, 1H); 8.15–8.06 (m, 1 H), 7.95–7.87 (m, 1H); 7.85–7.76 (m, 1H); 7.57–7.39 (m, 8H); 5.64 (s, 1H); 5.27 (d, *J* = 5.9, 1H); 4.60 (d, *J* = 3.5, 1H); 4.22–4.14 (m, 1H); 4.08–3.81 (m, 3H); 3.80–3.70 (m, 2H); 3.63 (t, *J* = 9.8 Hz, *J* = 4.7 Hz, 1H); 3.55–3.47 (m, 1H); 3.30 (s, 3H). ^13^C-NMR (100 MHz, DMSO-d_6_) *δ* 170.4; 136.6; 133.4; 133.3; 132.9; 132.0; 128.2; 128.1; 127.7; 126.9; 125.8; 125.5; 125.4; 124.2; 99.9; 98.6; 81.9; 68.0; 67.3; 62.4; 54.9; 54.3; 39.6. LC-MS *m*/*z* calculated for C_26 H27_ClNO_6_ [M+H]^+^ 484.1 found 484.1.

#### 4.10.18. Synthesis of Compound **18**

2-Naphthylacetyl chloride (0.27 mmol, 1.1 equiv) was synthesized using 2-naphthaleneacetic acid (50 mg, 0.27 mmol, 1.1 equiv) and oxalyl chloride (25 µL, 0.29 mmol, 1.2 equiv). The crude acid chloride, compound **4**, pyridine, and DCM were used. The reaction mixture was stirred at 0 °C for 3 h. Crude reaction mixture was purified via trituration using 5% MeOH/DCM (79 mg, 71%). R_f_ = 0.14 in 1% MeOH/DCM. mp 262.2–264.2 °C. ^1^H-NMR (400 MHz, DMSO-d_6_) *δ* 8.29 (d, *J* = 8.2 Hz); 7.91–7.80 (m, 3H); 7.77 (s, 1H); 7.53–7.41 (m, 7H); 5.64 (s, 1H); 5.30 (d, *J* = 5.9, 1H); 4.65 (d, *J* = 3.6); 4.23–4.15 (m, 1H); 3.91–3.83 (m, 1H); 3.79–3.58 (m, 5H); 3.55–3.47 (m, 1H); 3.29 (s, 3H). ^13^C-NMR (100 MHz, DMSO-d_6_) *δ* 170.3; 136.6; 134.2; 133.4; 132.9; 131.7; 128.2, 128.1, 127.6; 127.4; 127.3; 127.1; 125.9; 125.4; 99.9; 98.6; 81.9; 68.0; 67.3; 62.4; 54.9; 54.3; 42.1. LC-MS *m*/*z* calculated for C_26 H27_ClNO_6_ [M+H]^+^ 484.1 found 484.1.

## Figures and Tables

**Table 1:** Gelation properties of the amides **3**–**18**.

Comp #	R =	Hex.	Tol.	n-BuOH	n-PrOH	i-PrOH	EtOH	TEG	EG	Gly	H_2_O	EtOH/H_2_O1:1	EtOH/H_2_O1:2	DMSO/H_2_O1:1	DMSO/H_2_O1:2
**3**	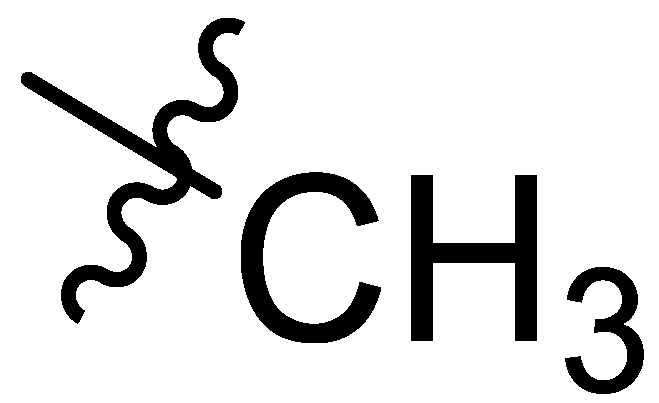	I	G2.9_O_	G10.0_T_	G3.3_O_	G20.0_O_	G20.0_O_	G10.0_T_	G3.3_C_	G4.0_C_	I	G6.7_O_	P	G20.0_O_	G5.0_O_
**5**	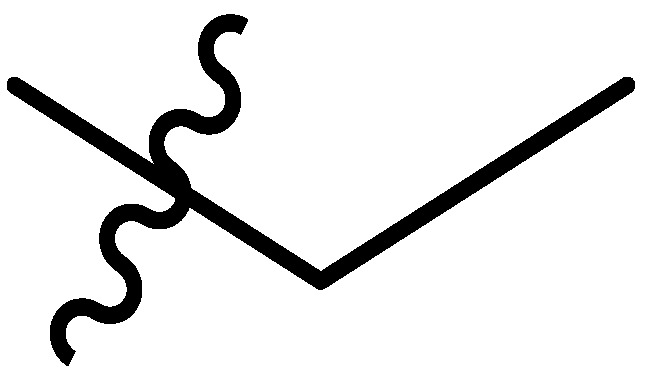	I	P	P	G20.0_O_	G20.0_O_	G10.0_O_	S	G6.7_c_	I	I	P	P	P	P
**6**	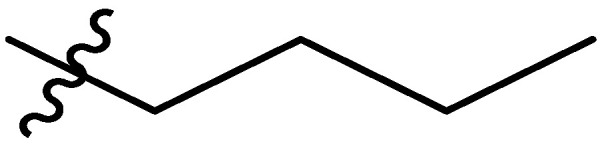	I	P	G1.0_O_	G20.0_O_	G6.7_T_	G20.0_T_	S	G6.7_T_	I	I	G0.74_T_	G10.0_O_	0.74_T_	G1.0_T_
**7**	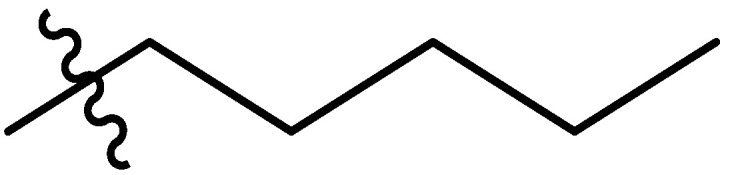	I	G10.0_O_	G20.0_T_	P	G20.0_T_	G20.0_T_	G20.0_O_	G20.0_T_	I	I	P	P	G0.91_T_	G6.7_T_
**8**	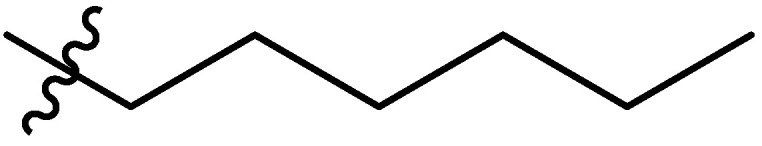	I	G10.0_T_	G10.0_O_	G20.0_O_	G10.0_O_	P	S	I	I	I	P	P	G1.3_T_	G0.80_T_
**9**	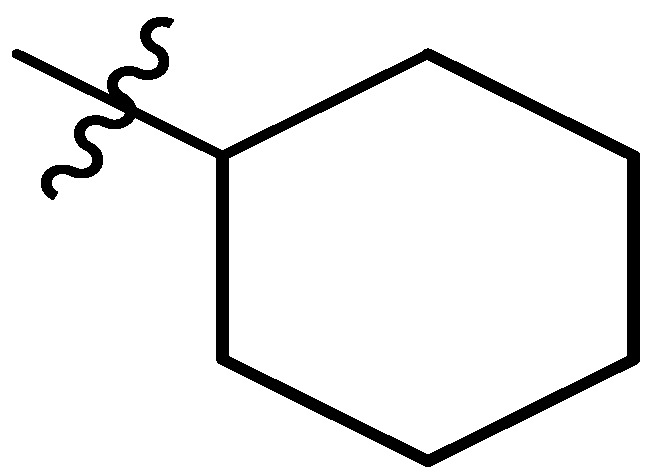	I	G20.0_C_	G6.7_O_	G6.7_O_	G3.3_C_	G6.7_T_	S	G6.7_T_	G3.3_C_	I	G1.3_T_	G2.5_T_	G1.5_T_	G2.2_T_
**10**	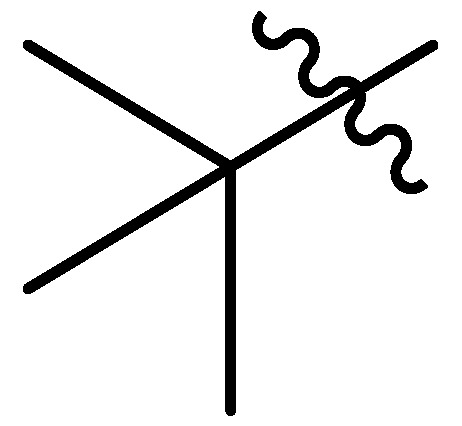	G5.0_O_	S	S	S	S	S	S	G20.0_O_	G20.0_O_	I	G20.0_O_	P	G20.0_O_	P
**11**	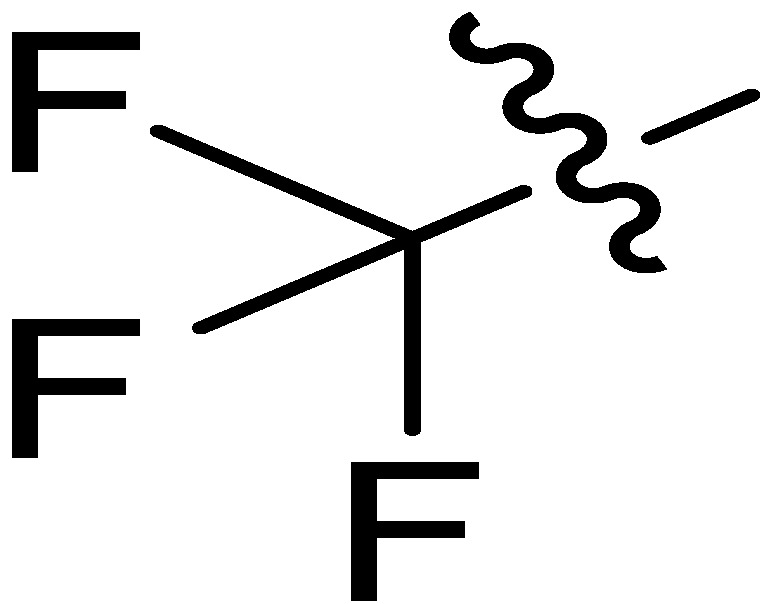	I	P	G20.0_O_	G10.0_O_	G20.0_O_	G10.0_C_	S	G10.0_C_	I	I	G2.0_C_	G1.4_T_	G2.2_C_	G1.5_C_
**12**	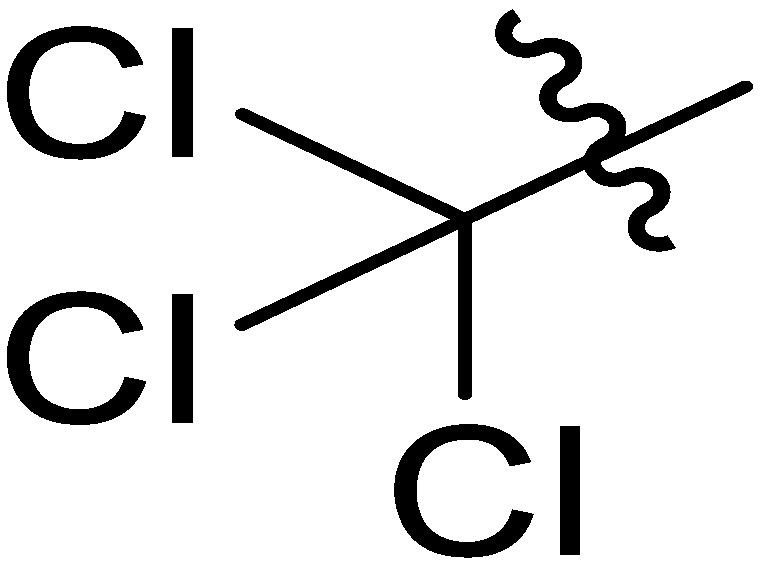	I	S	S	S	S	S	S	S	G10.0_O_	I	G0.67_C_	G0.91_T_	G2.0_T_	G3.3_T_
**13**	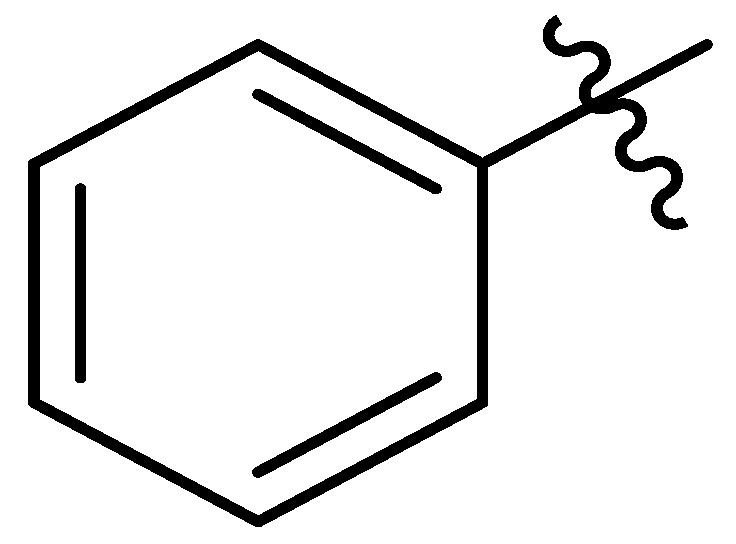	I	G10.0_T_	P	P	P	G5.0_O_	S	G10.0_O_	G5.0_O_	G0.36_T_	G0.54_C_	G0.36_T_	G0.54_C_	G0.46_C_
**14**	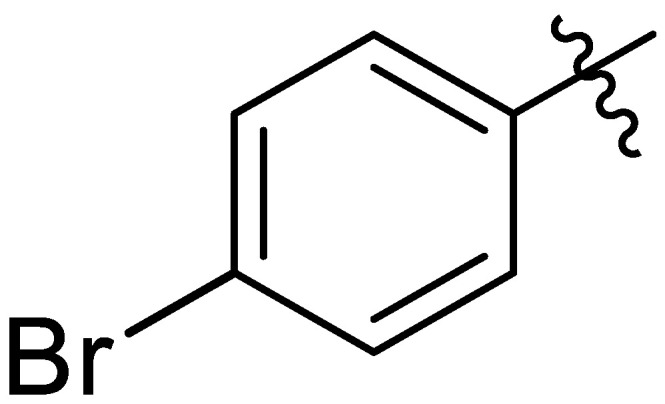	I	I	G20.0_O_	P	I	P	S	G10.0_O_	I	I	I	I	G20.0_O_	G20.0_O_
**15**	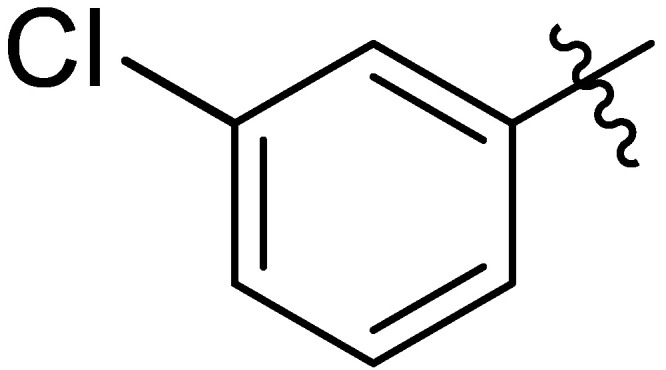	I	G10.0_O_	G10.0_O_	G10.0_O_	G6.7_O_	P	S	G10.0_O_	G6.7_O_	I	G1.3_O_	G10.0_O_	G0.71_O_	G1.3_O_
**16**	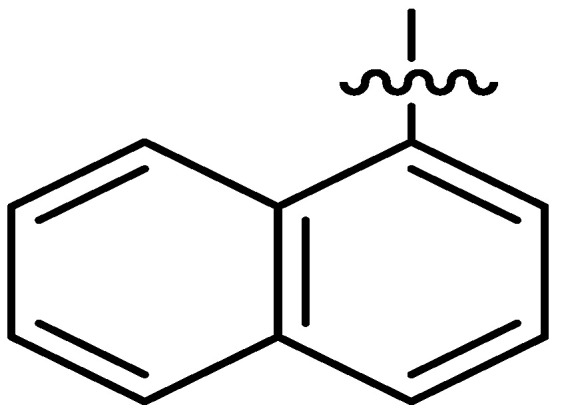	I	P	G10.0_O_	G10.0_C_	G10.0_O_	G20.0_C_	S	G10.0_O_	G6.7_T_	I	I	I	G5.0_O_	G2.9_O_
**17**	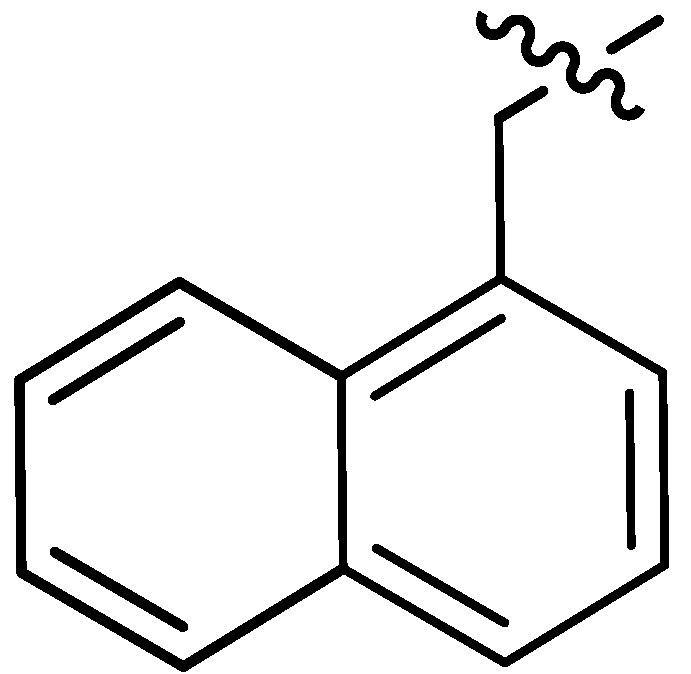	I	P	P	G10.0_T_	I	I	S	G5.0_T_	G6.7_T_	I	P	I	P	I
**18**	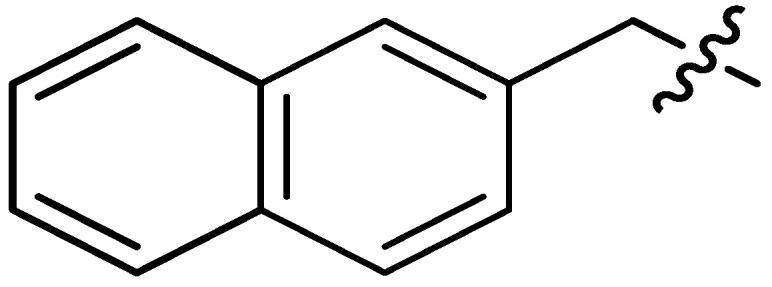	I	P	I	I	I	I	S	G10.0_T_	I	I	I	I	P	I

All compounds were tested starting from 20 mg/mL. G, stable gel at room temperature, the numbers close to G indicate the MGCs in mg/mL units; P, precipitation; S, soluble; I, insoluble; O, opaque; T, translucent; C, clear, all ratios in mixed solvents are in volume ratio.

## Data Availability

Not applicable.

## Supplementary Materials

The following are available online at https://www.mdpi.com/article/10.3390/gels7030134/s1. (I) ^1^ H- and ^13^C NMR spectra for compounds **3**–**18**, (II) 2D HSQC and COSY NMR spectra for selected compounds, (III) stacked NMR spectra for variable temperature studies for compounds **3**, **11** and **13** in Figures S1–S3, (IV) additional rheology properties for super gelators, including amplitude sweeps and frequency sweeps in Figures S4-1 to S4-8 and rheological data in Table S1a–i; (V) gel extrusion studies with rheology amplitude experiments in Figures S5–S7 and Tables S2 and S3, (VI) chloramphenicol release study in Figures S8 and S9 and naproxen release study in Figures S10 and S11, UV-vis spectra of compound **13** in Figure S12; (VII) UV-vis spectra of toluidine blue and calibration curve in Figures S13 and S14, additional dye absorption studies in Figures S15–S17; (VIII) LCMS traces for compounds **3**–**18**.
