# Supplementary material for: Fine-Tuning of Molecular Structures to Generate Carbohydrate Based Super Gelators and Their Applications for Drug Delivery and Dye Absorption
Source: Gels. 2021 Sep 7;7(3):134. doi: 10.3390/gels7030134 (PMC8482264; doi:10.3390/gels7030134)

Supplementary Information

Fine-tuning of molecular structures to generate carbohydrate based super gelators and their applications for drug delivery and dye absorption

Jonathan Bietsch, Mary Olson, Guijun Wang* Department of Chemistry and Biochemistry, Old Dominion University, Norfolk, VA 23529, g1wang@odu.edu

Table of contents

I. ^{1}H and ^{13}C NMR spectra of compounds **3-18** S2

II. 2D NMR spectra of selected compounds S18

III. ^{1}H NMR spectra at different temperatures S23

IV. Rheological properties of selected gels with MGCs under 1 mg/mL S30

V. Gel extrusion studies with rheology amplitude experiments S39

VI. 1. Chloramphenicol release from gels S47

2. Naproxen release from gels S50

3. UV-vis spectra of compound **13** S53

VII.1. Calibration curve for TBO S54

2. Dye absorption studies using the gels formed by compound **13**.......... S55

VIII. LCMS traces for compounds **3-18** S57

I. ^{1}H and ^{13}C NMR spectra of compounds **3-18**

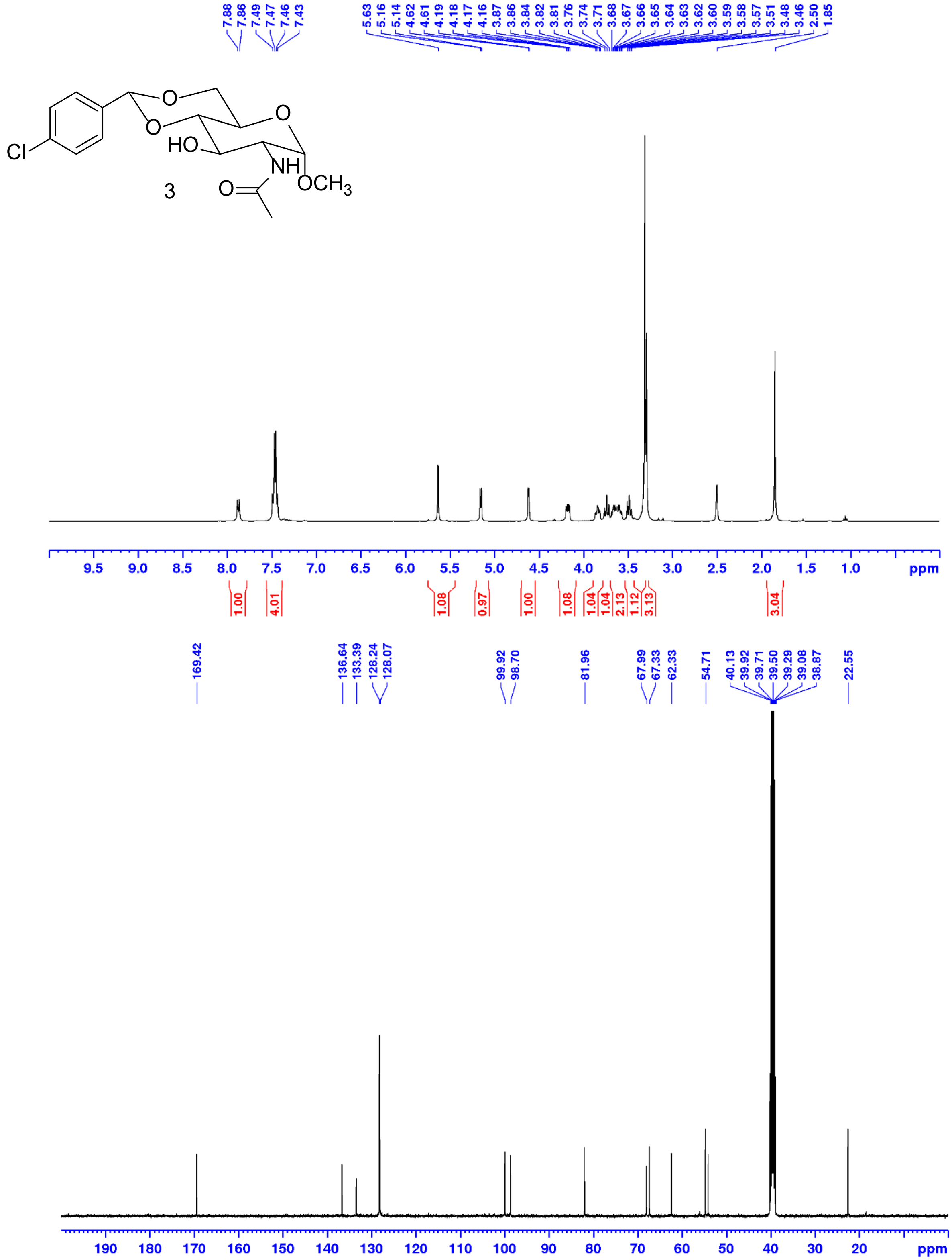

^{1}H and ^{13}C NMR spectra of compound **3** in d_6-DMSO

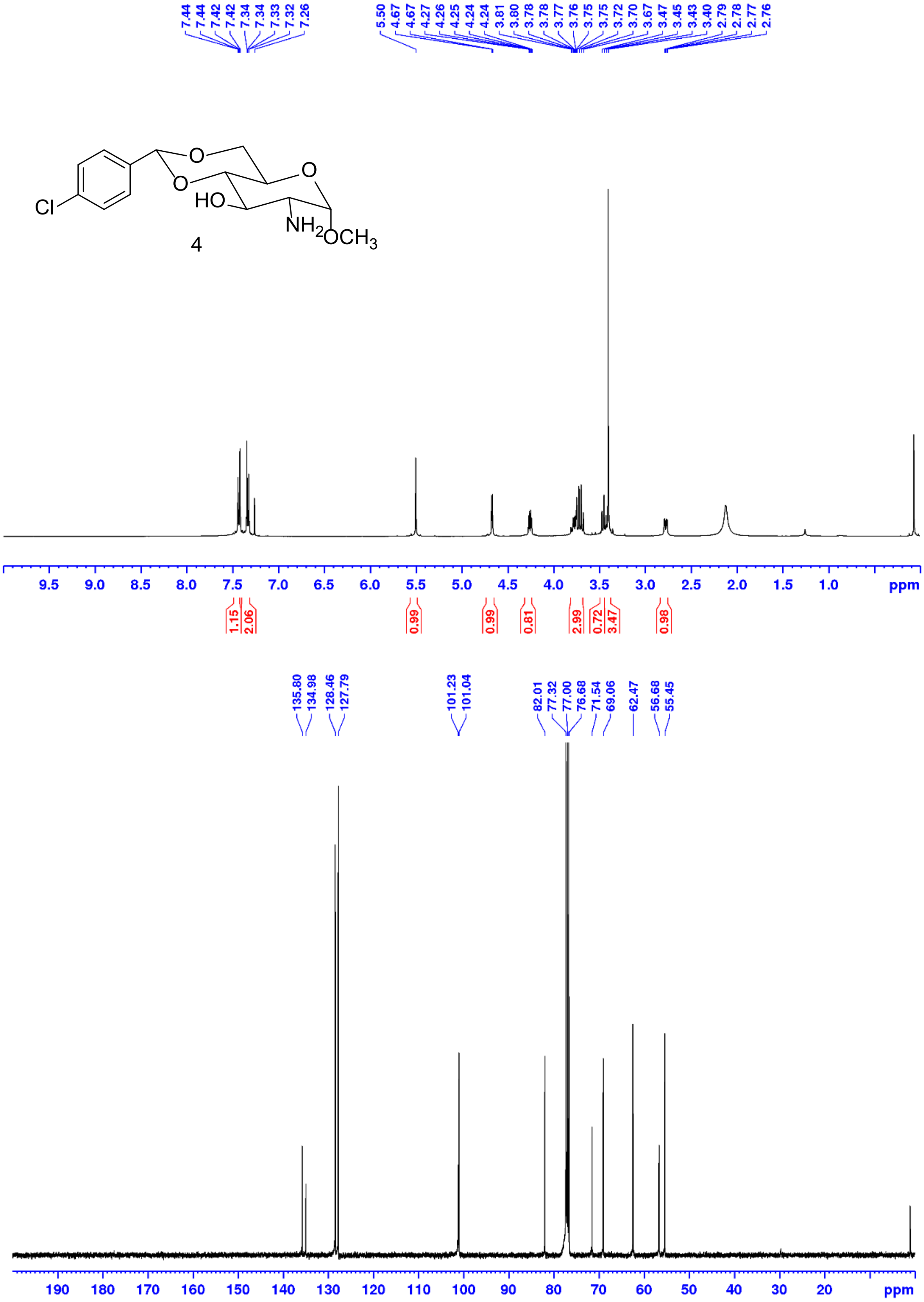

^{1}H and ^{13}C NMR spectra of compound **4** in $CDCl_3$

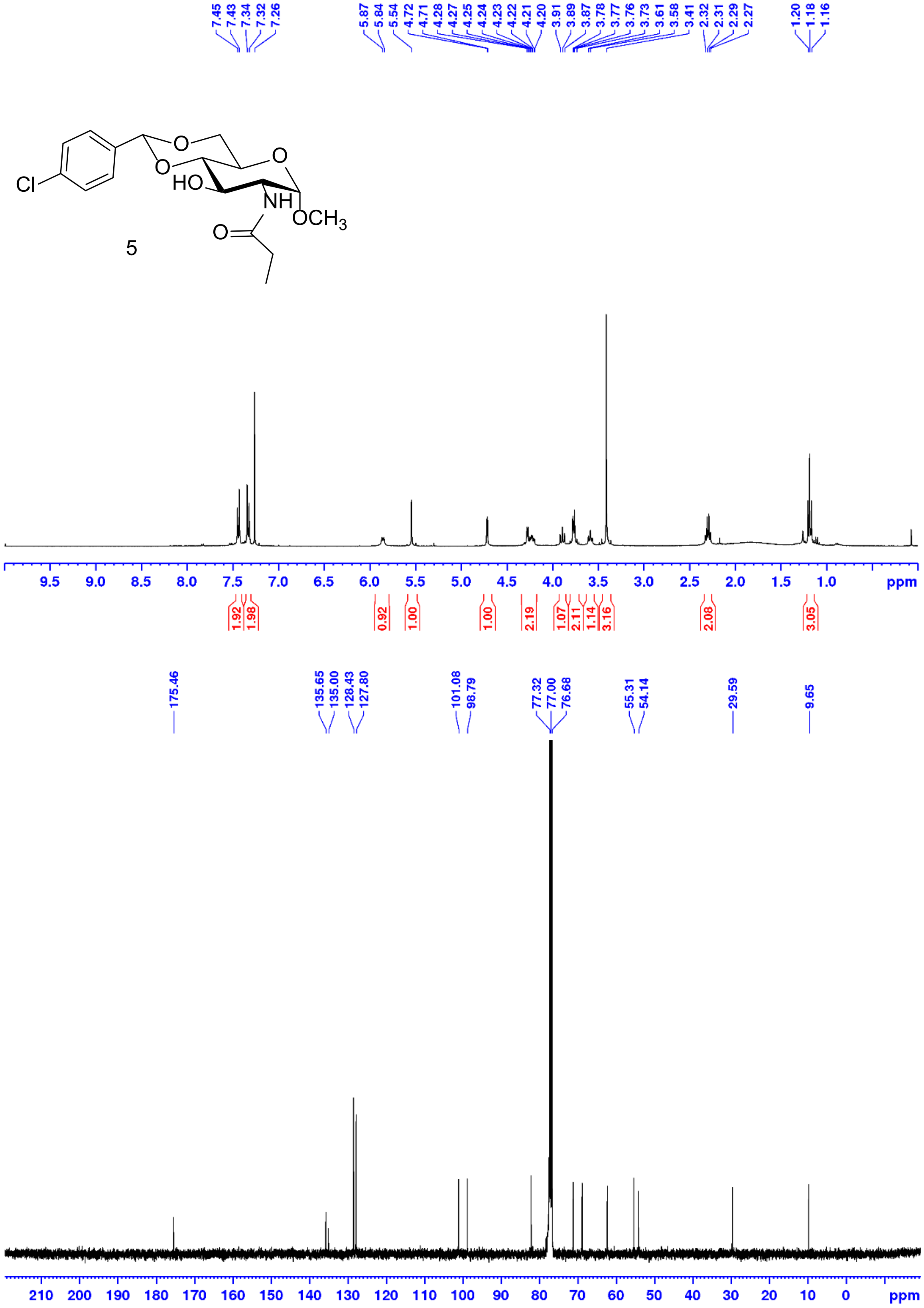

^{1}H and ^{13}C NMR spectra of compound **5** in $CDCl_3$

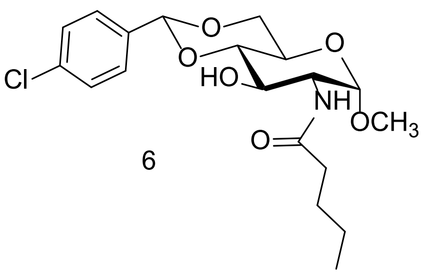

6

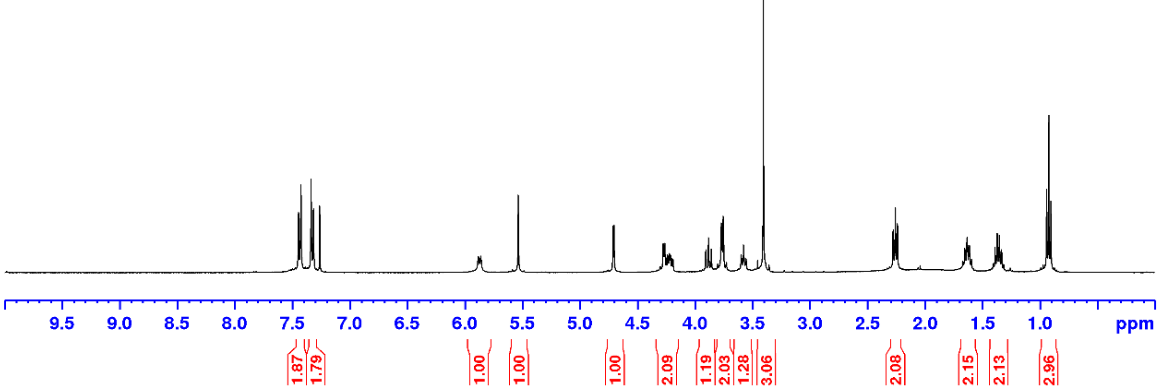

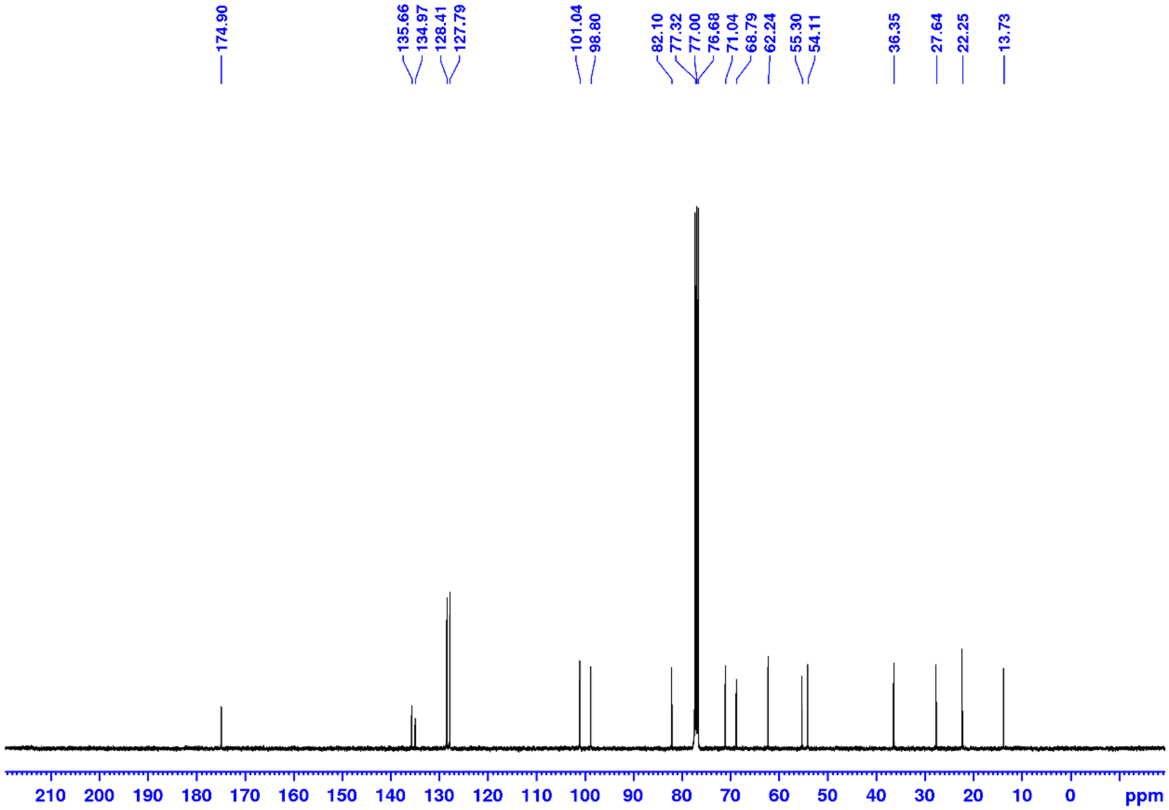

^{1}H and ^{13}C NMR spectra of compound **6** in $CDCl_3$

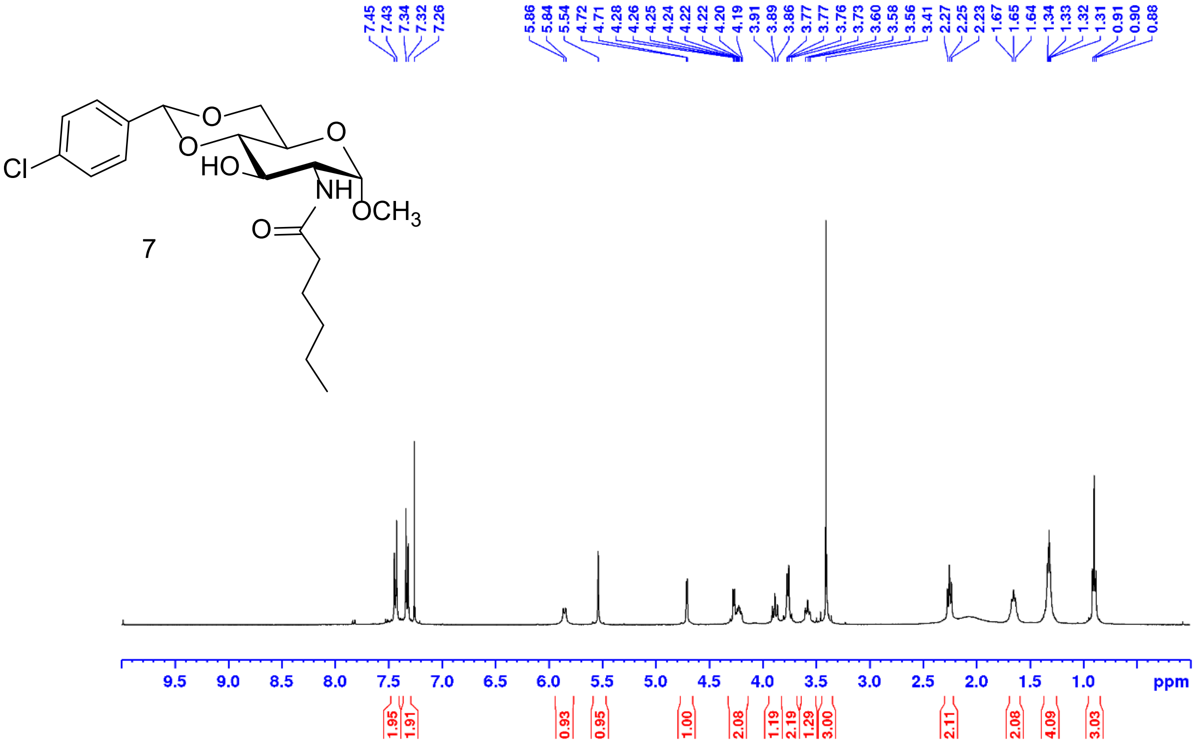

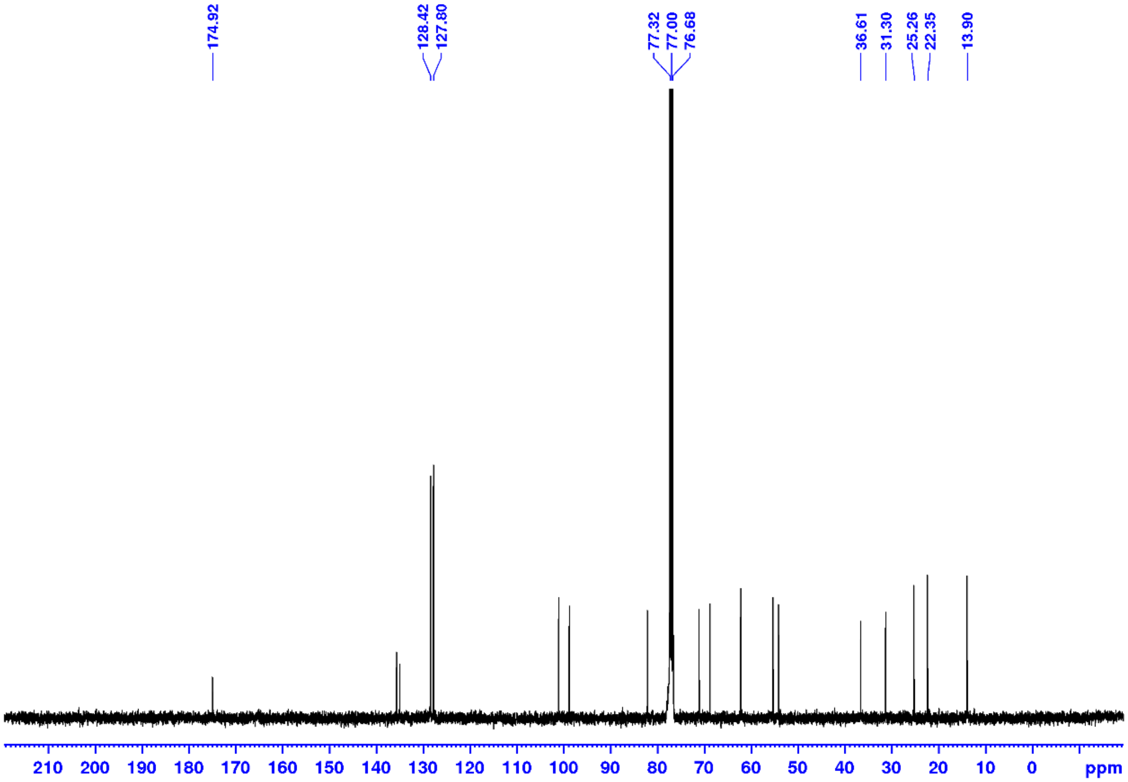

^{1}H and ^{13}C NMR spectra for compound **7** in $CDCl_3$

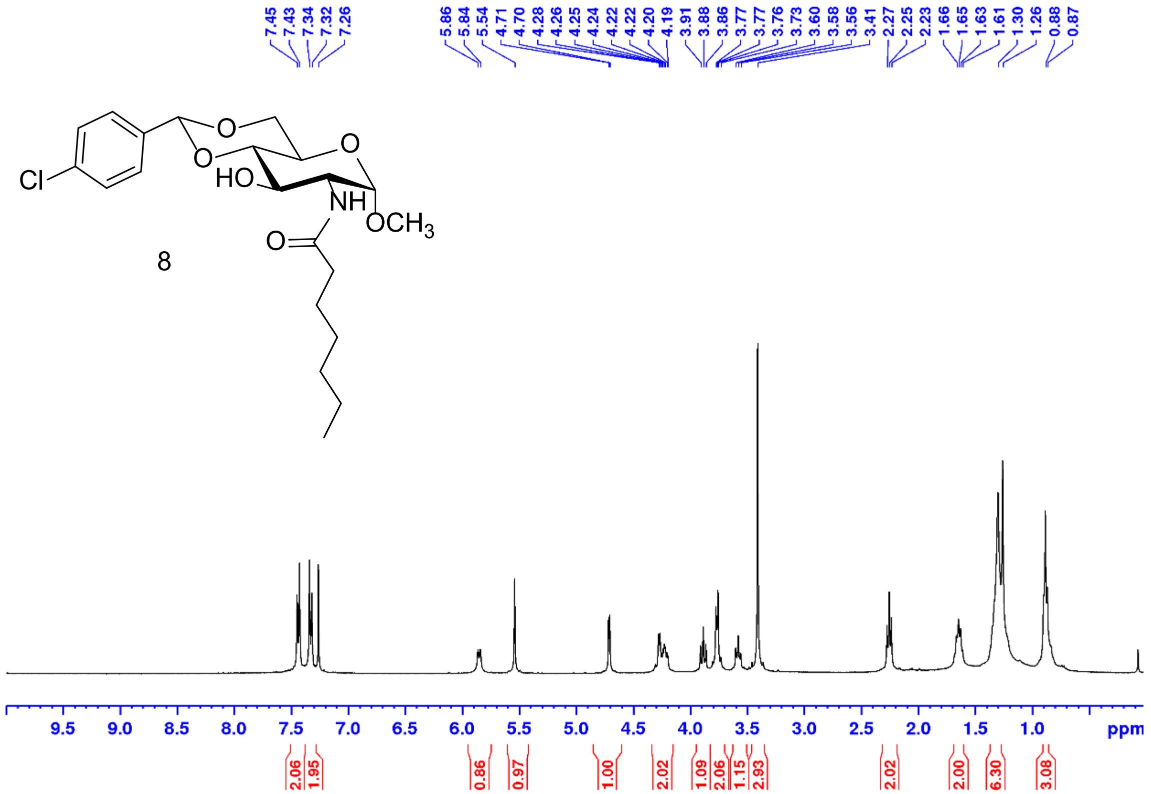

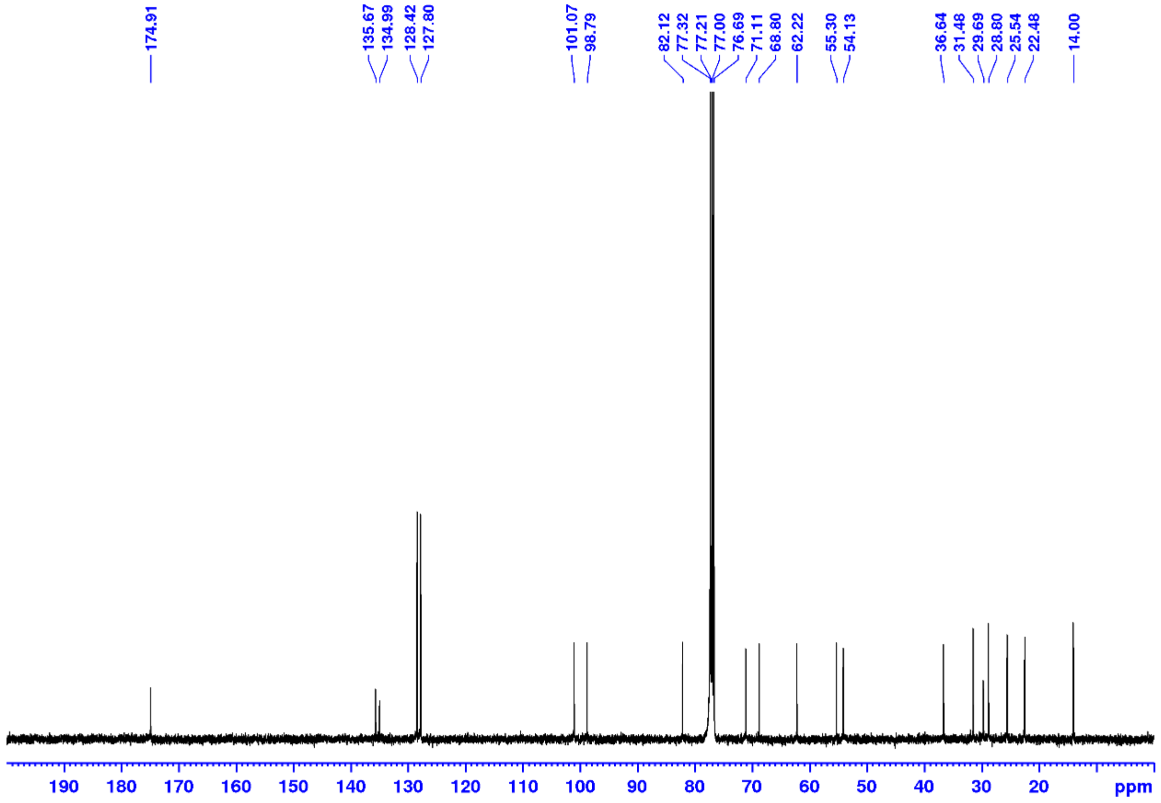

^{1}H and ^{13}C NMR spectra for compound **8** in $CDCl_3$

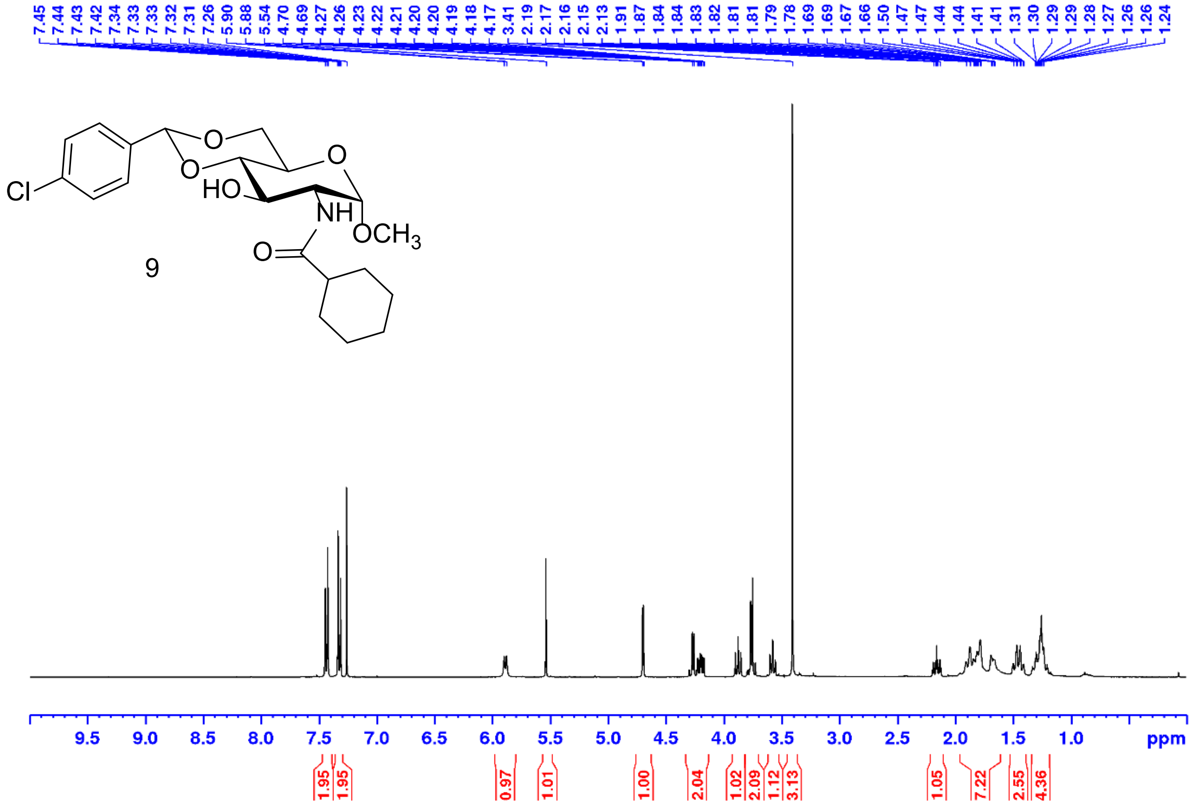

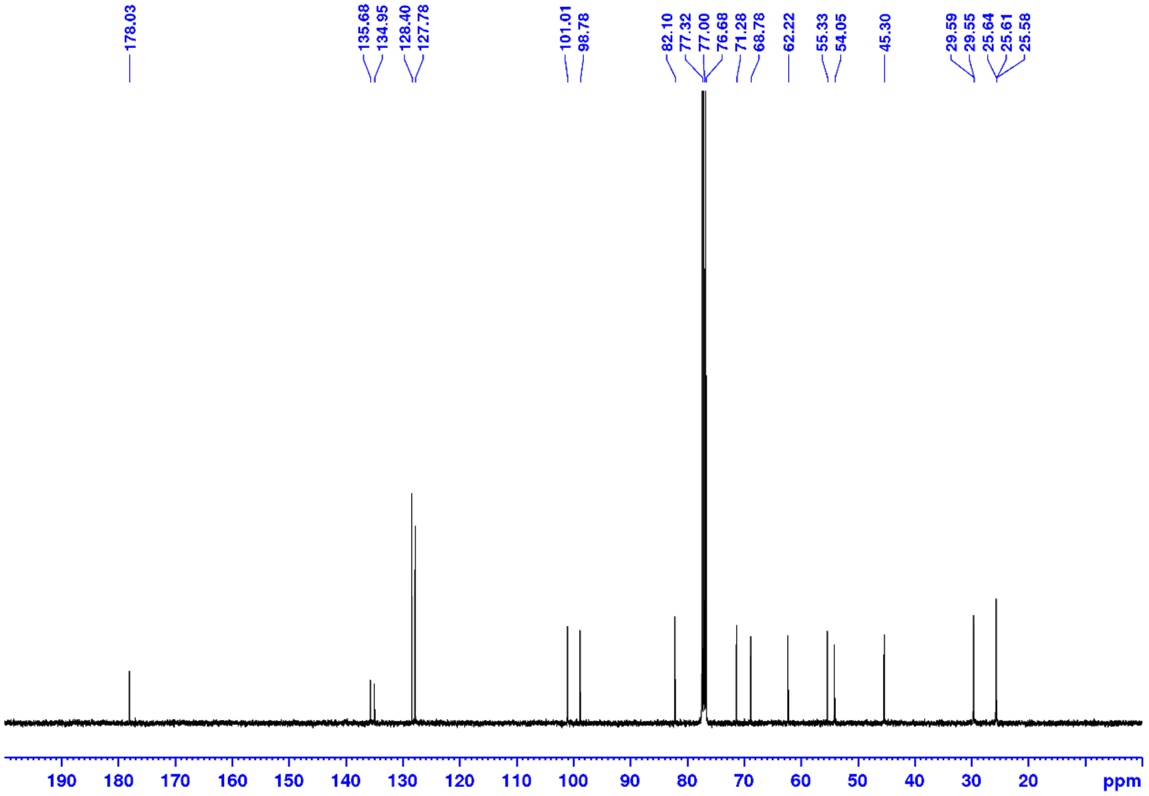

^{1}H and ^{13}C NMR spectra for compound **9** in $CDCl_3$

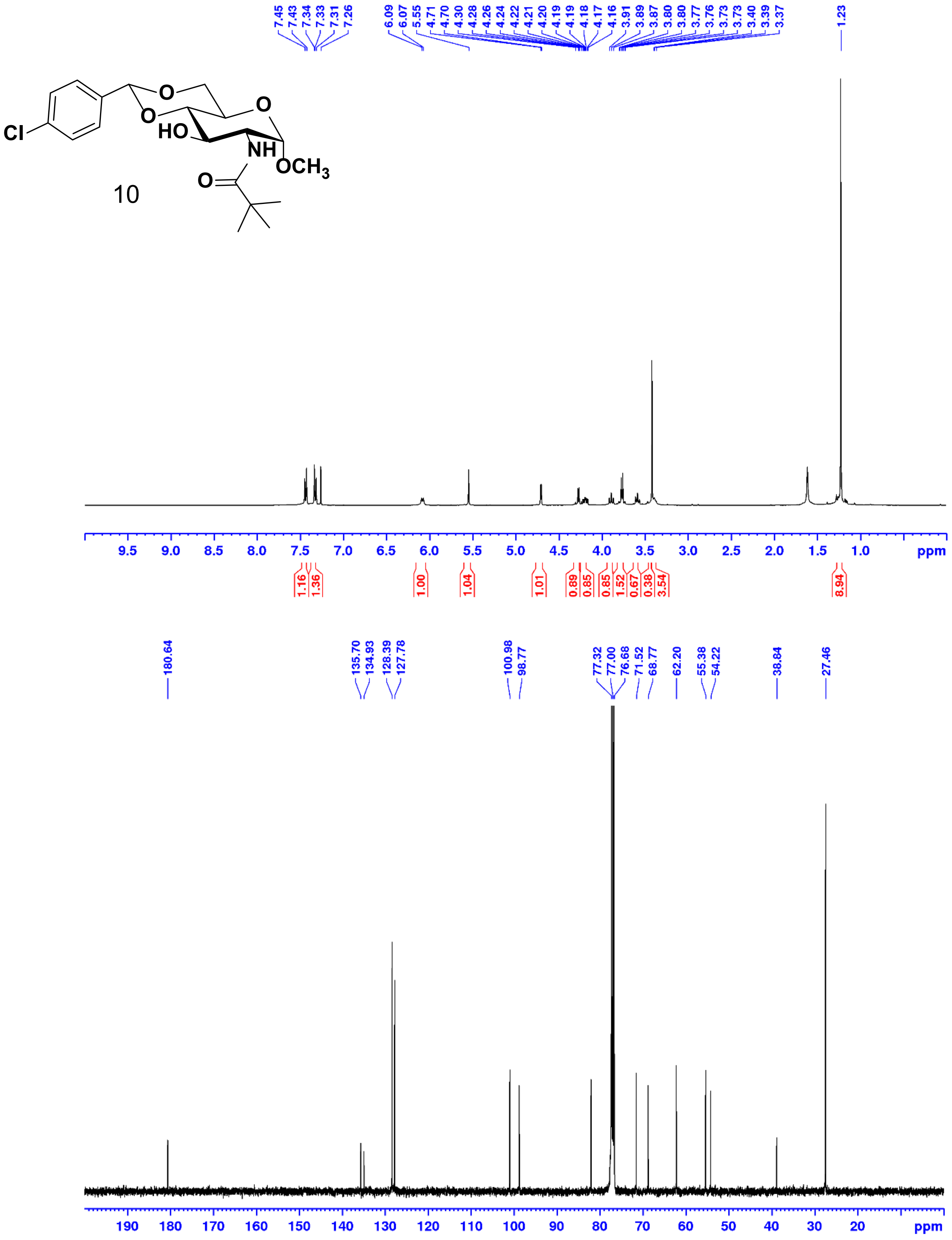

10

^1H and ^{13}C NMR spectra for compound **10** in $CDCl_3$

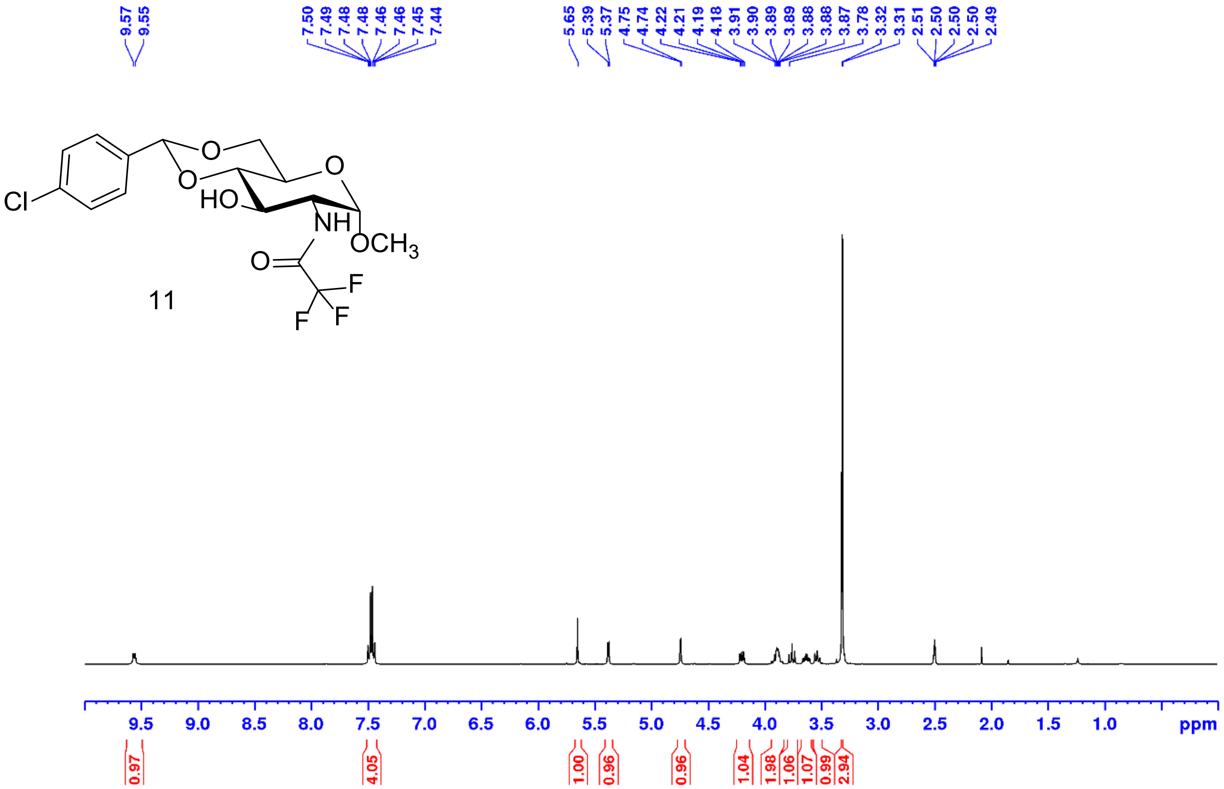

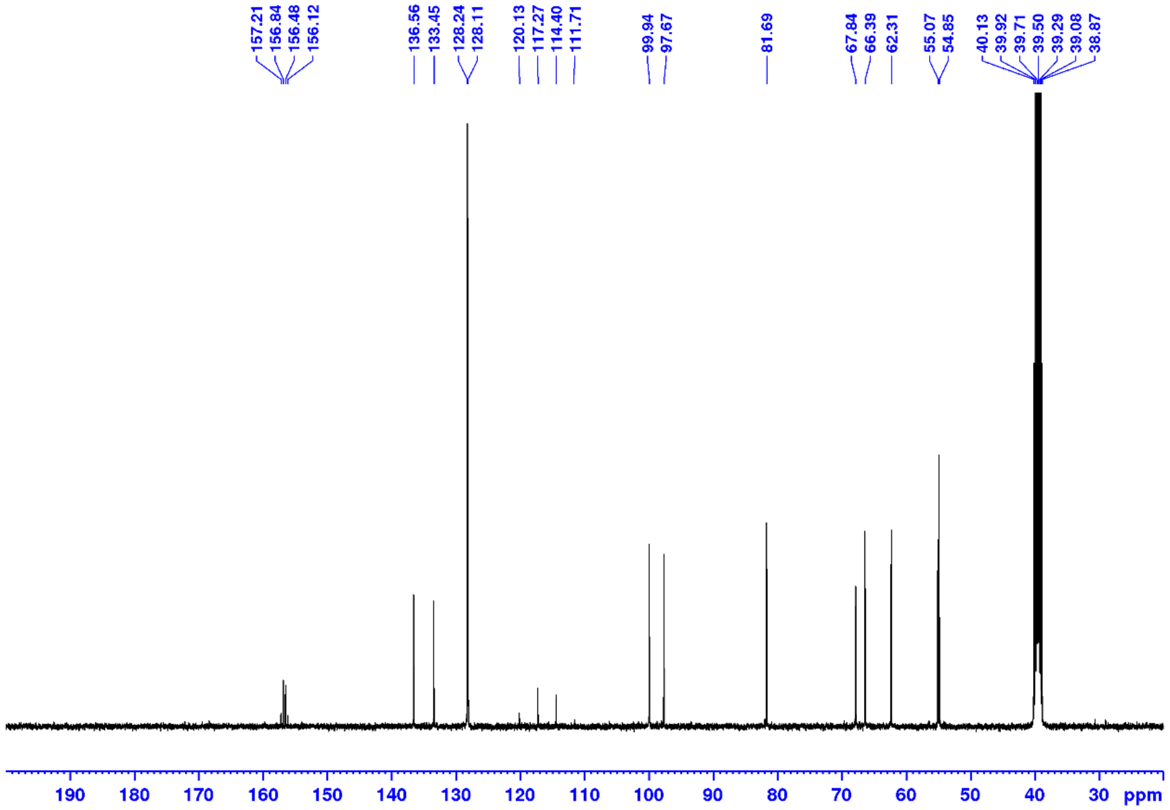

^{1}H and ^{13}C NMR spectra for compound **11** in d_6-DMSO

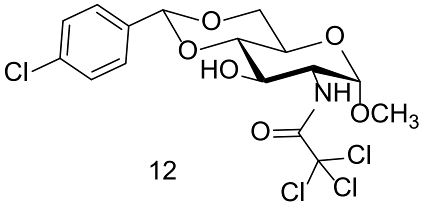

12

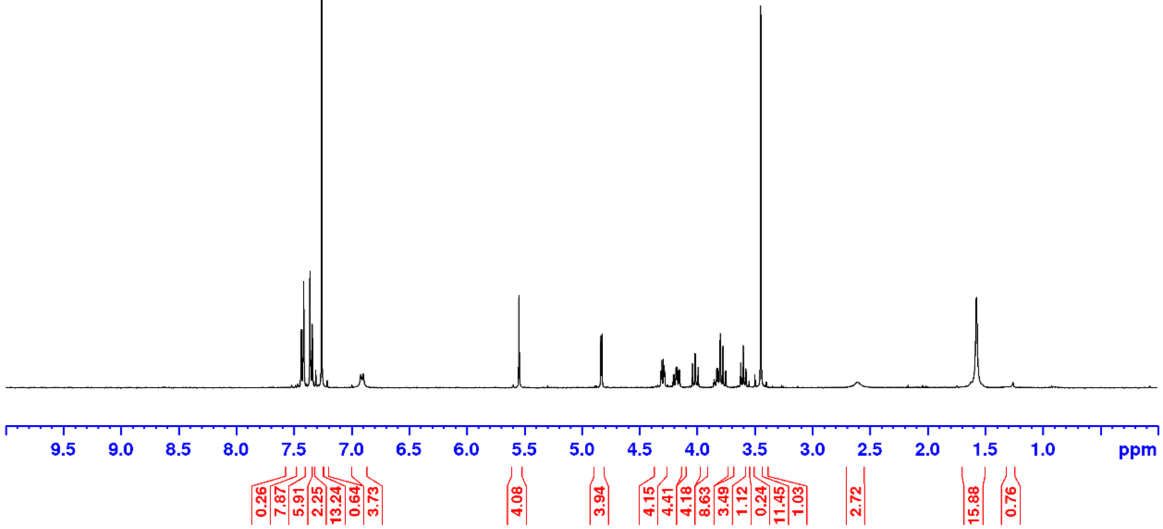

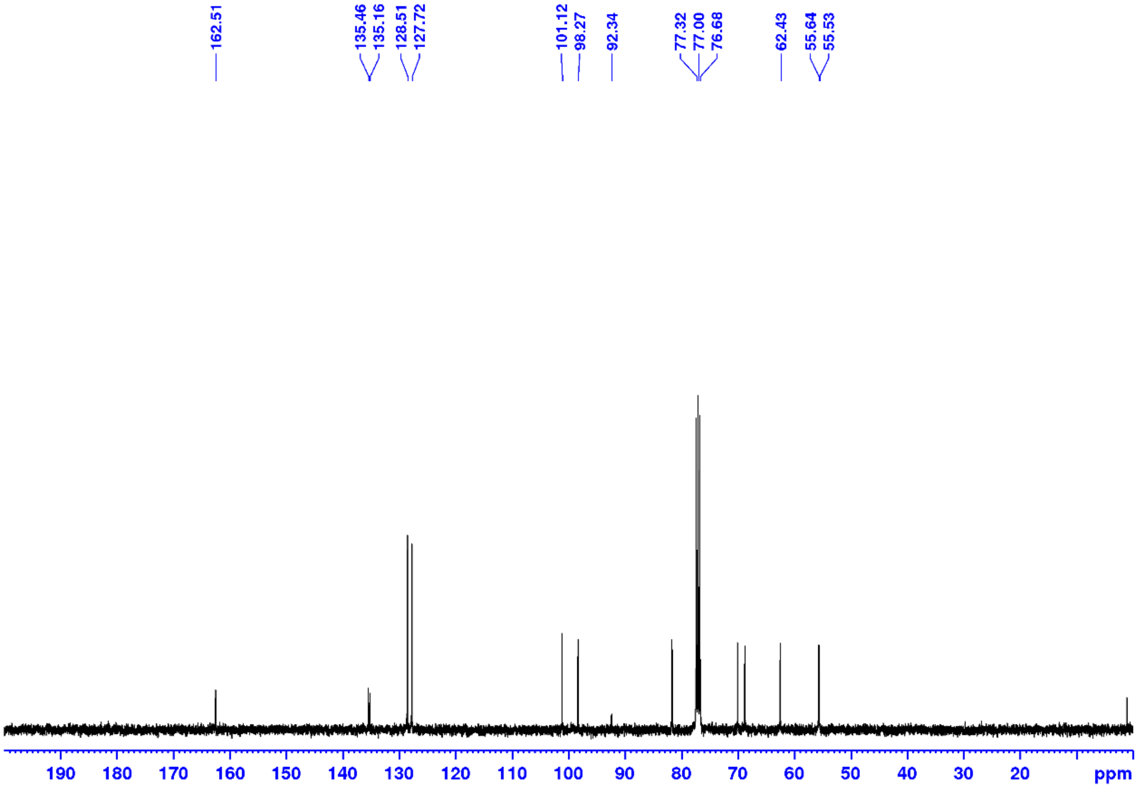

^{1}H and ^{13}C NMR spectra for compound **12** in $CDCl_3$

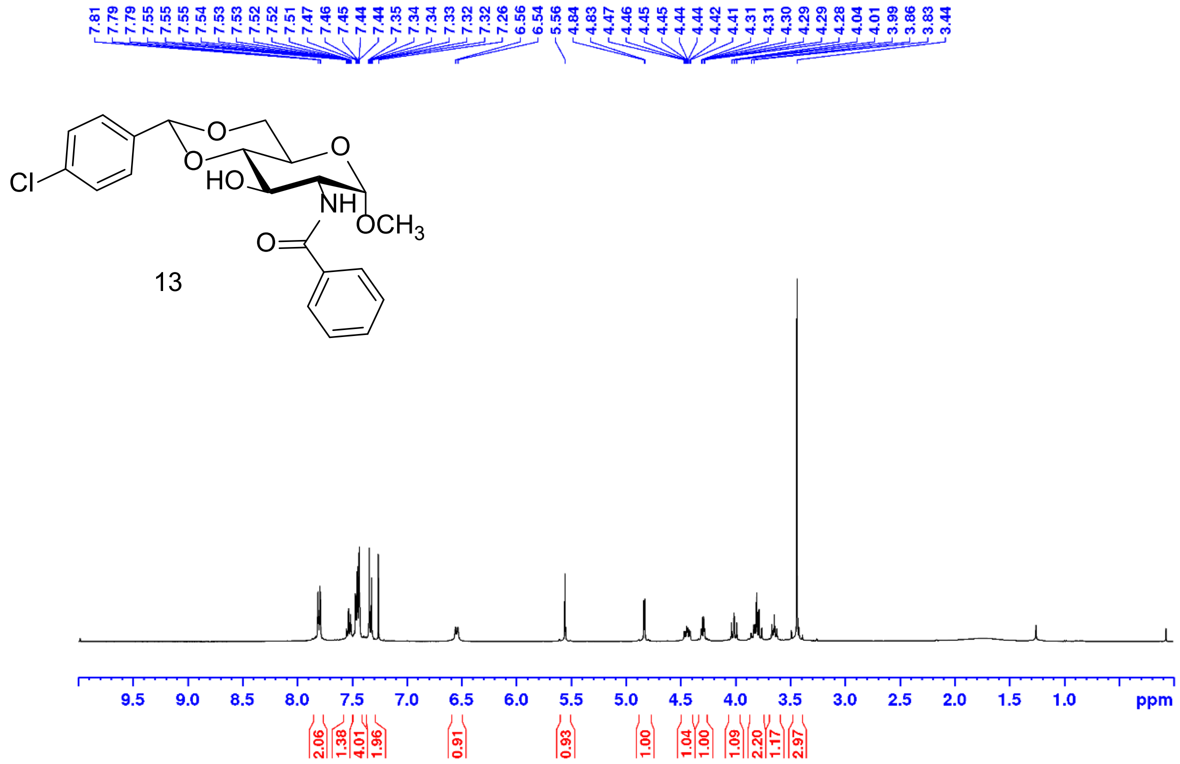

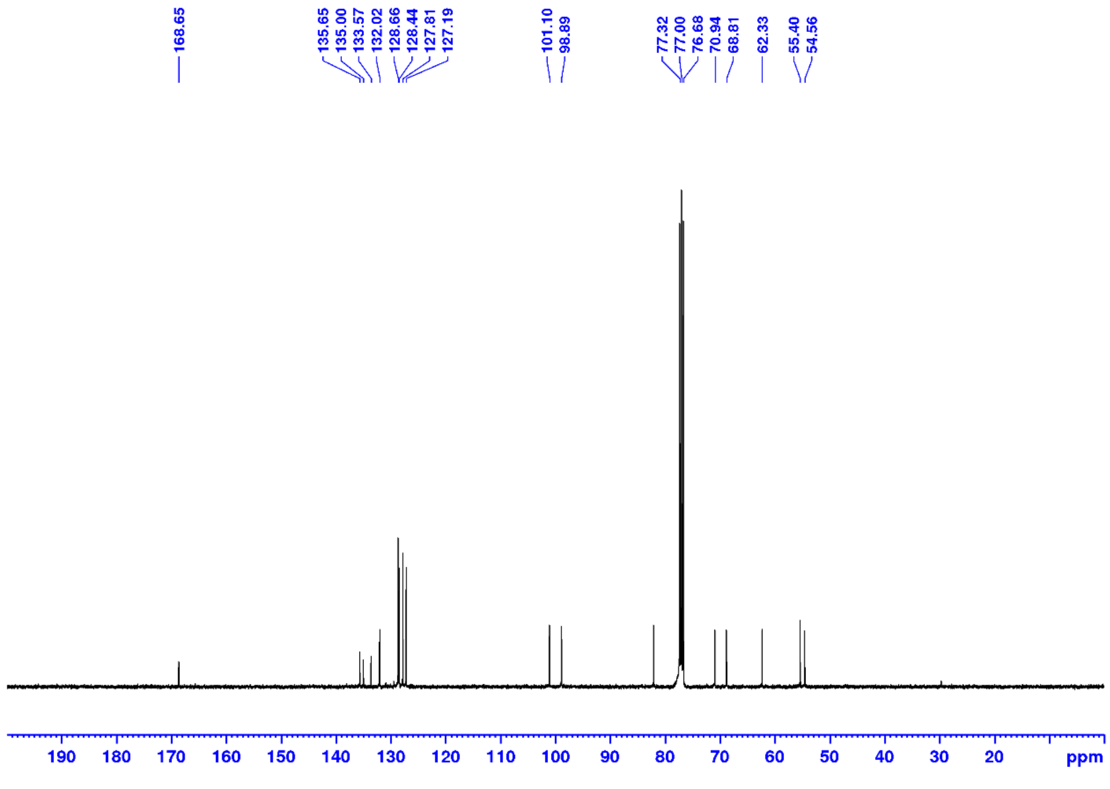

^{1}H and ^{13}C NMR spectra for compound **13** in $CDCl_3$

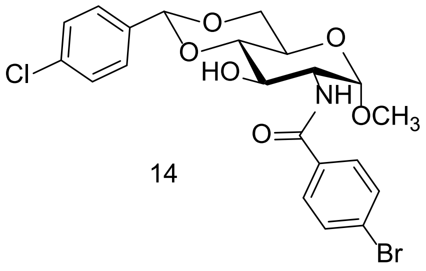

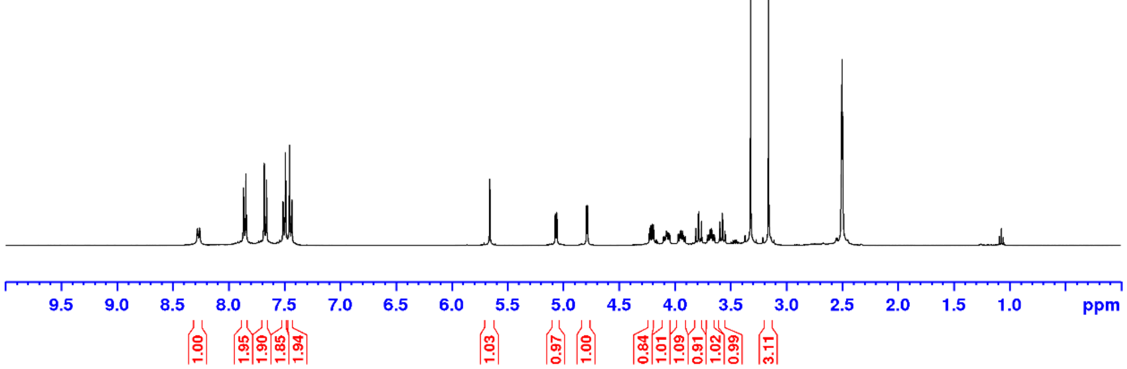

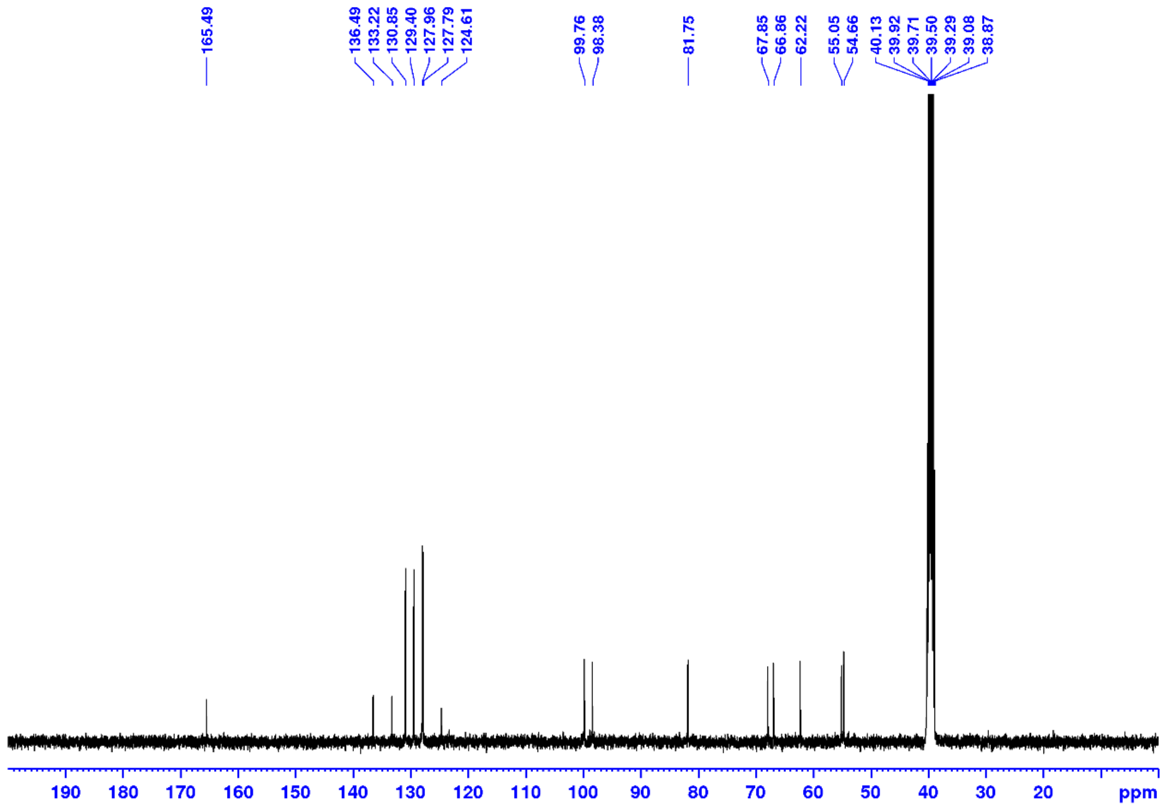

^{1}H and ^{13}C NMR spectra for compound **14** in d_6-DMSO

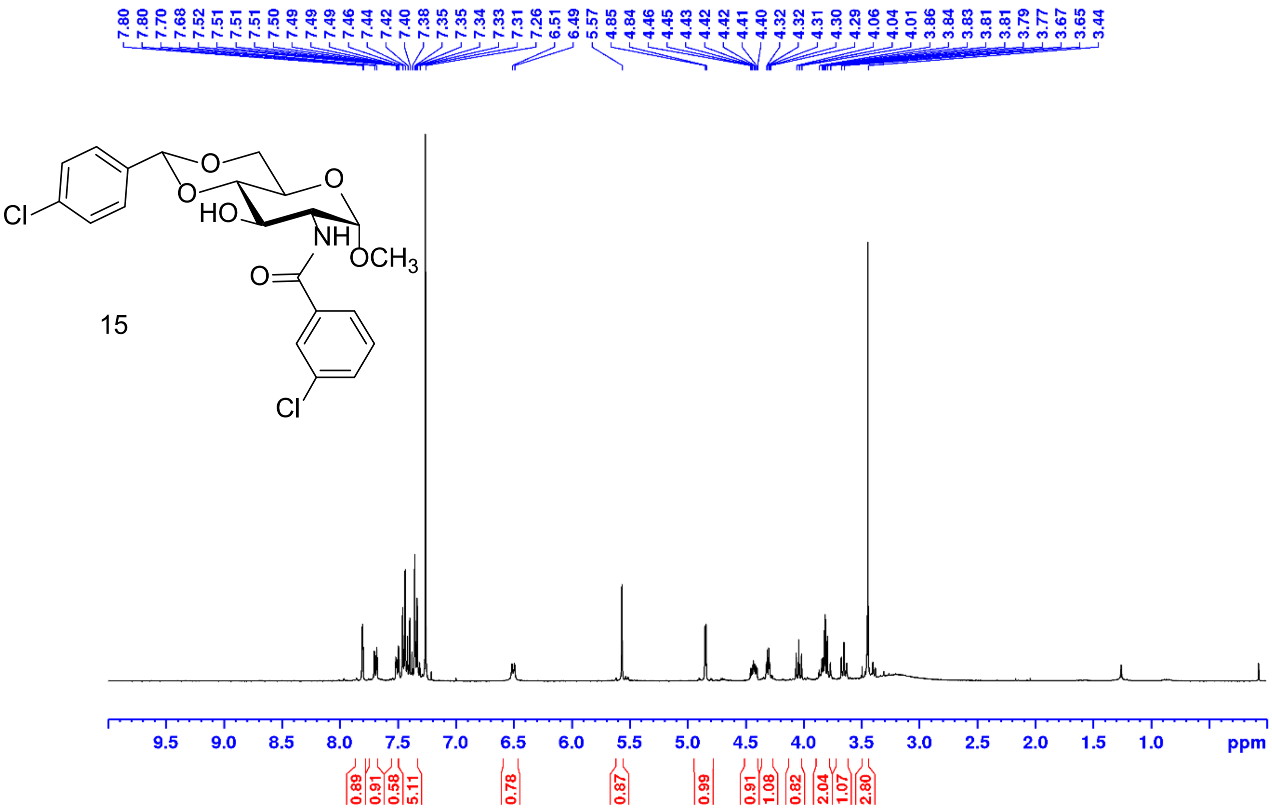

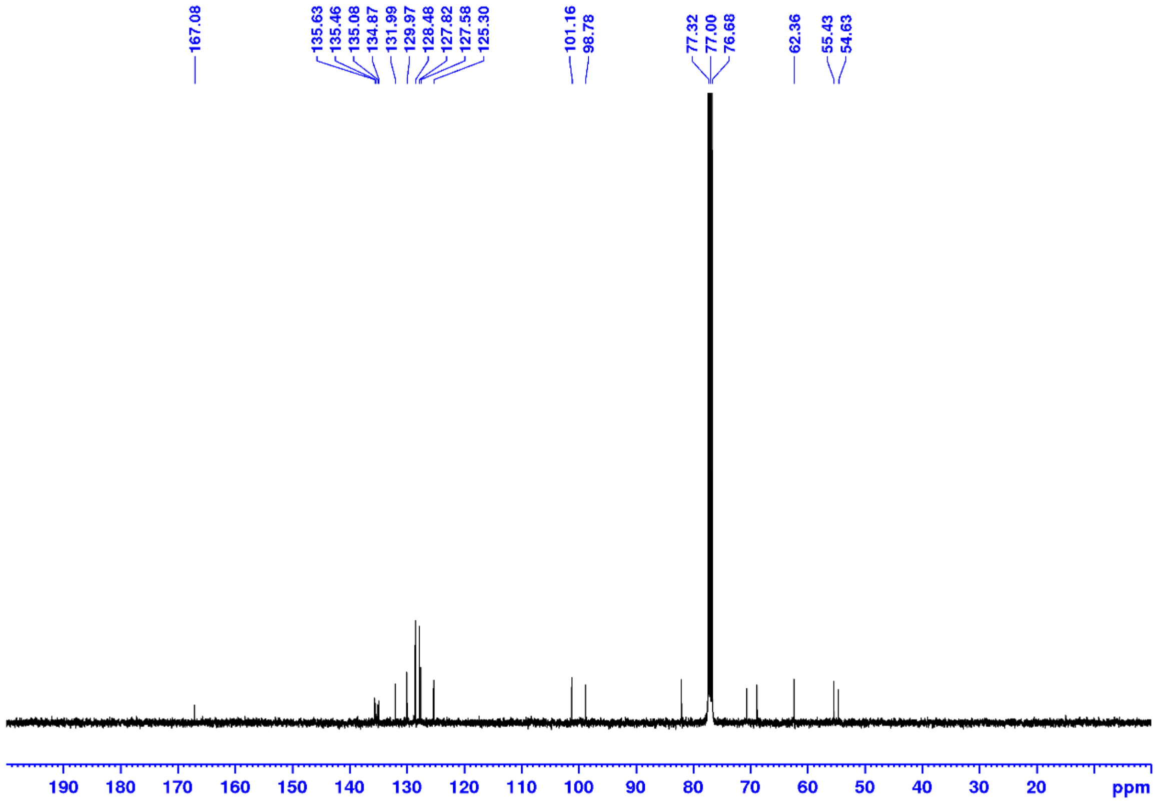

^{1}H and ^{13}C NMR spectra for compound **15** in $CDCl_3$

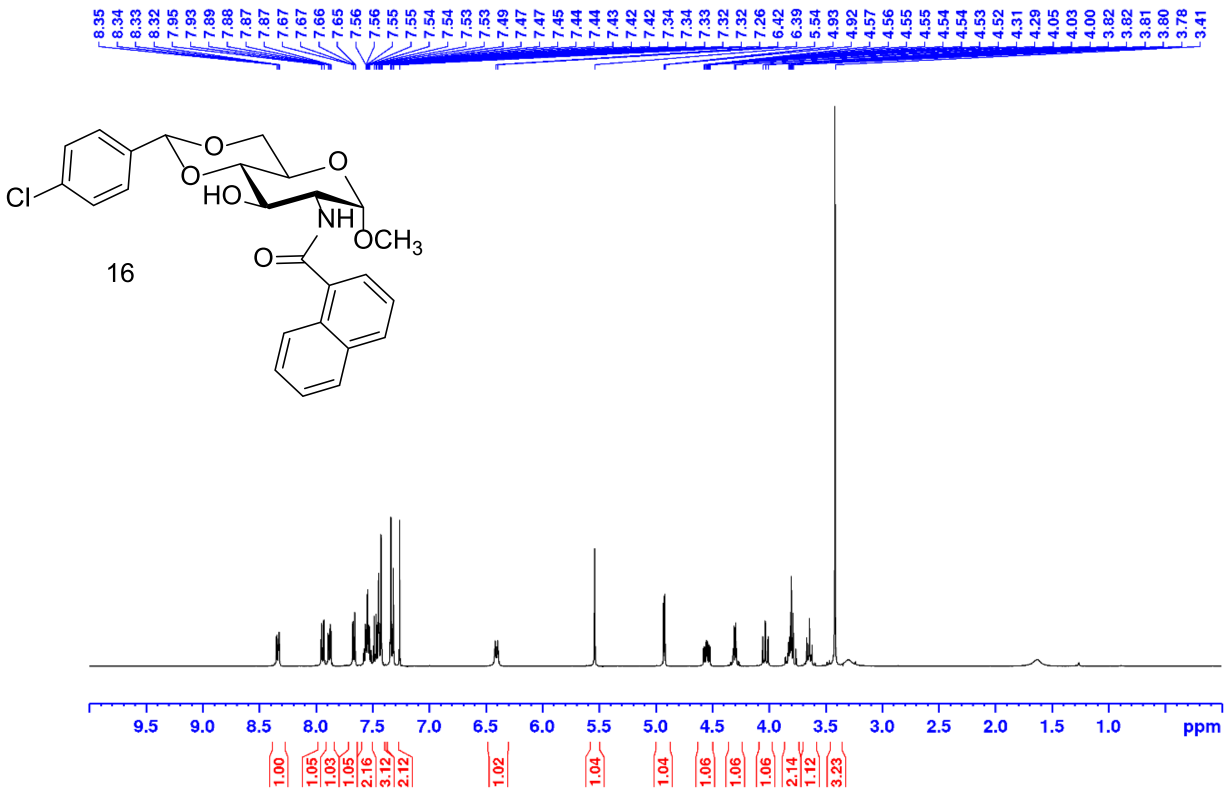

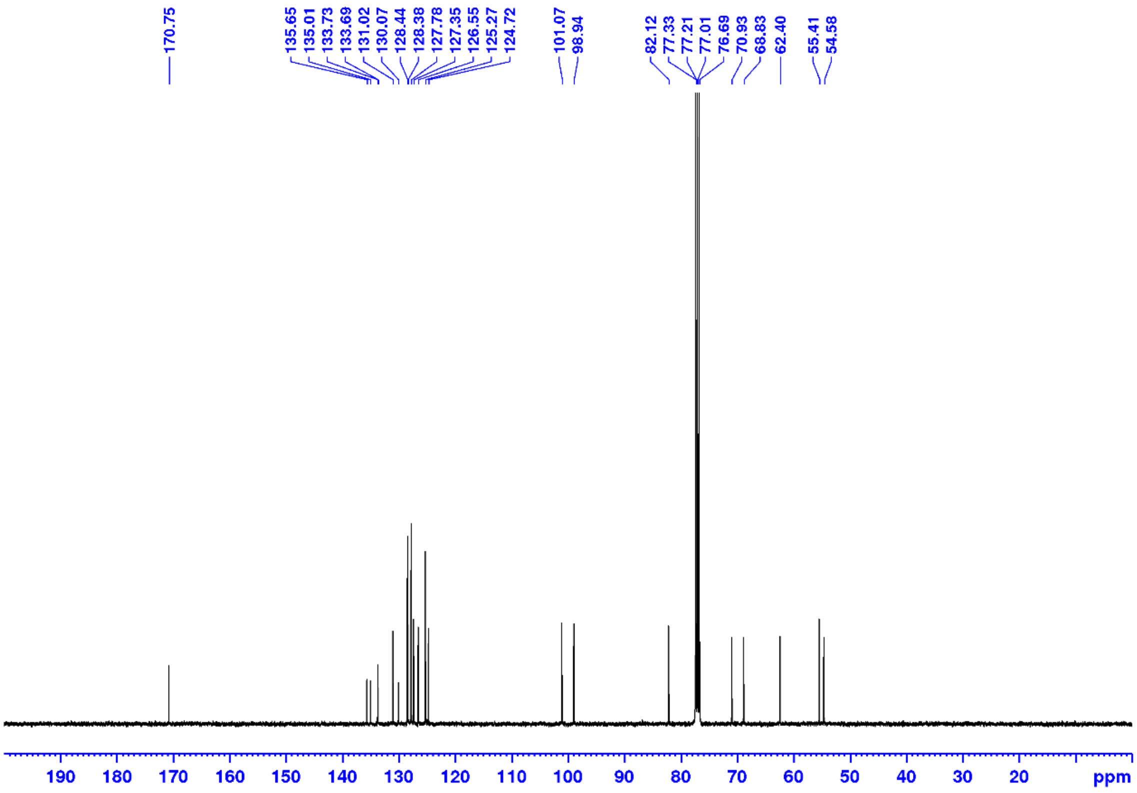

^{1}H and ^{13}C NMR spectra for compound **16** in $CDCl_3$

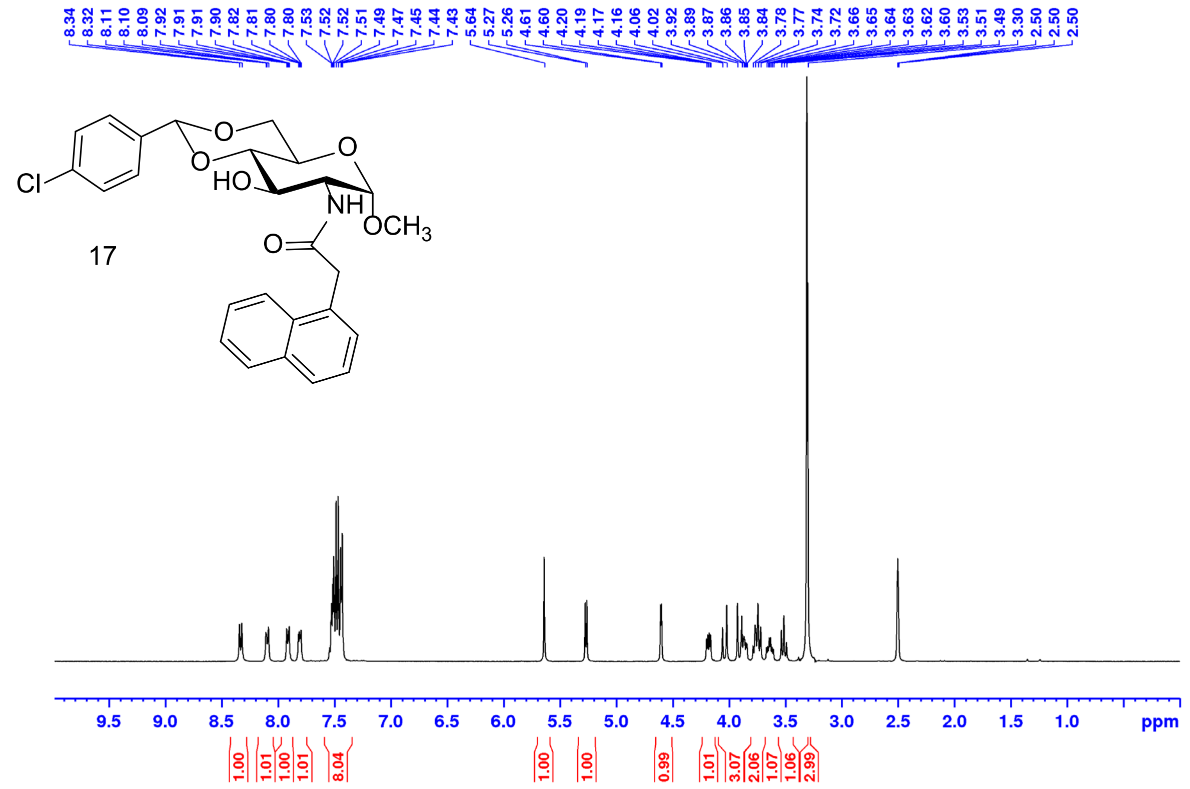

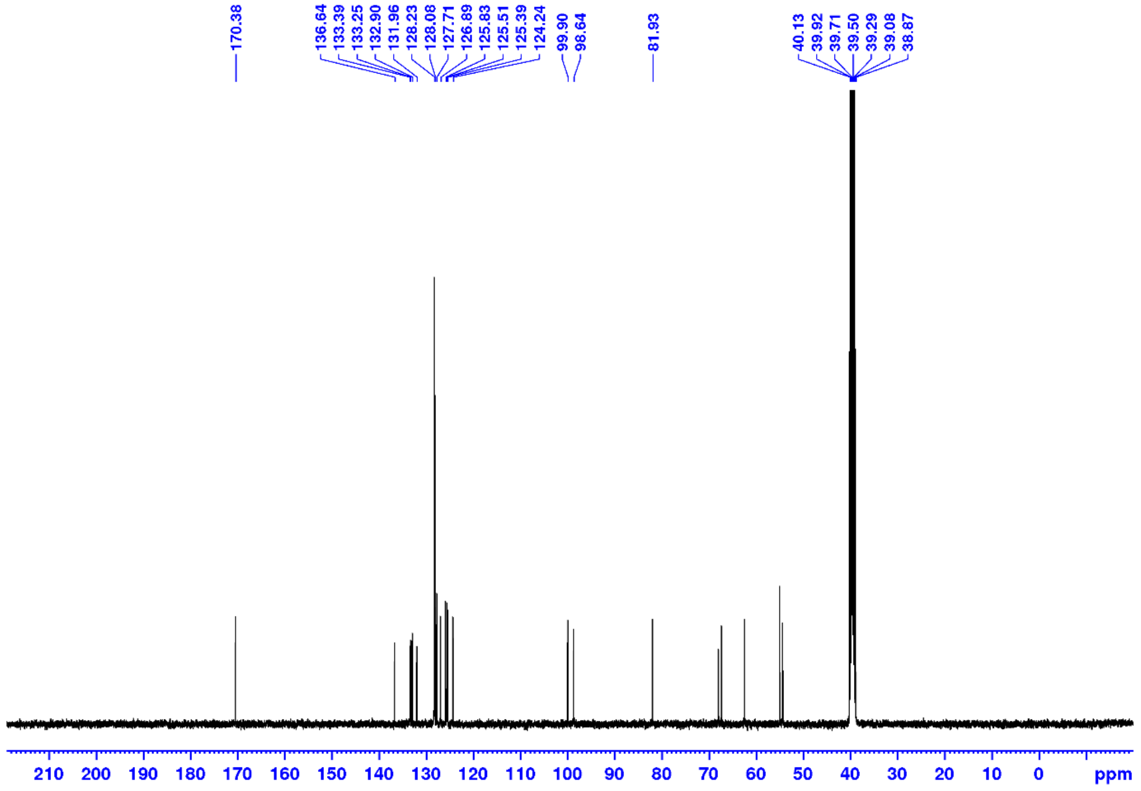

^{1}H and ^{13}C NMR spectra for compound **17** in d_6-DMSO

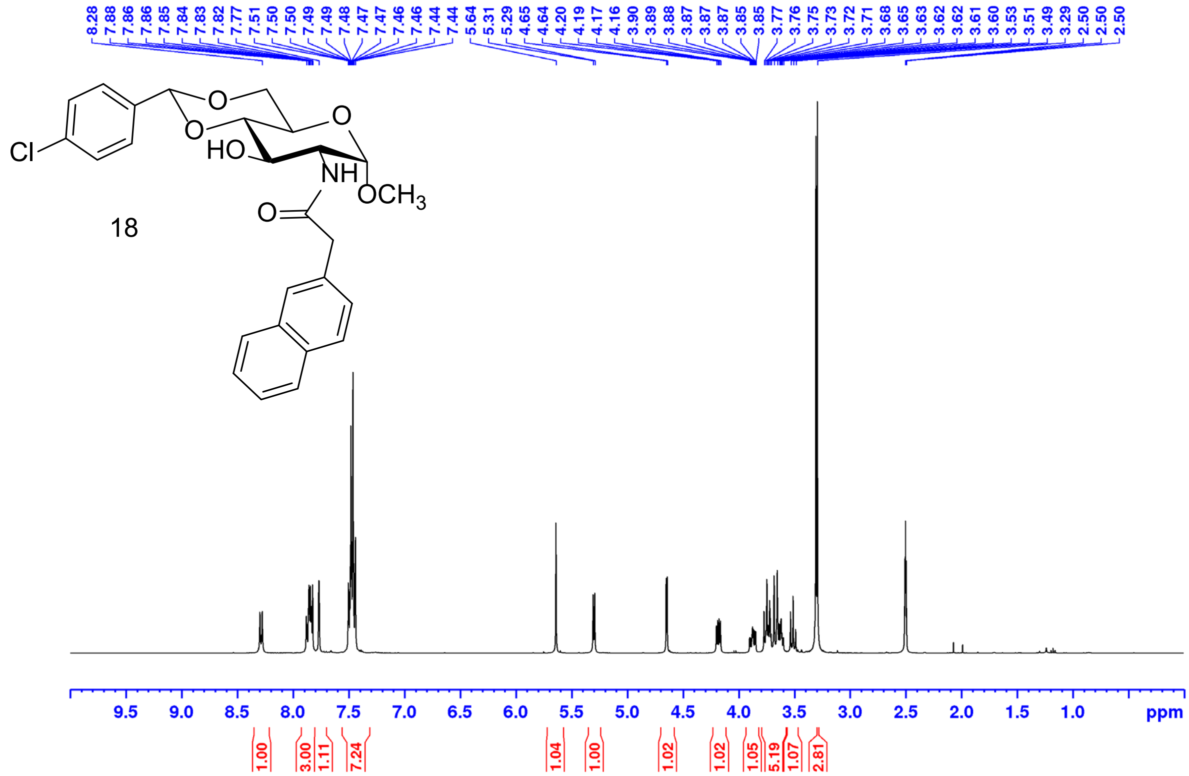

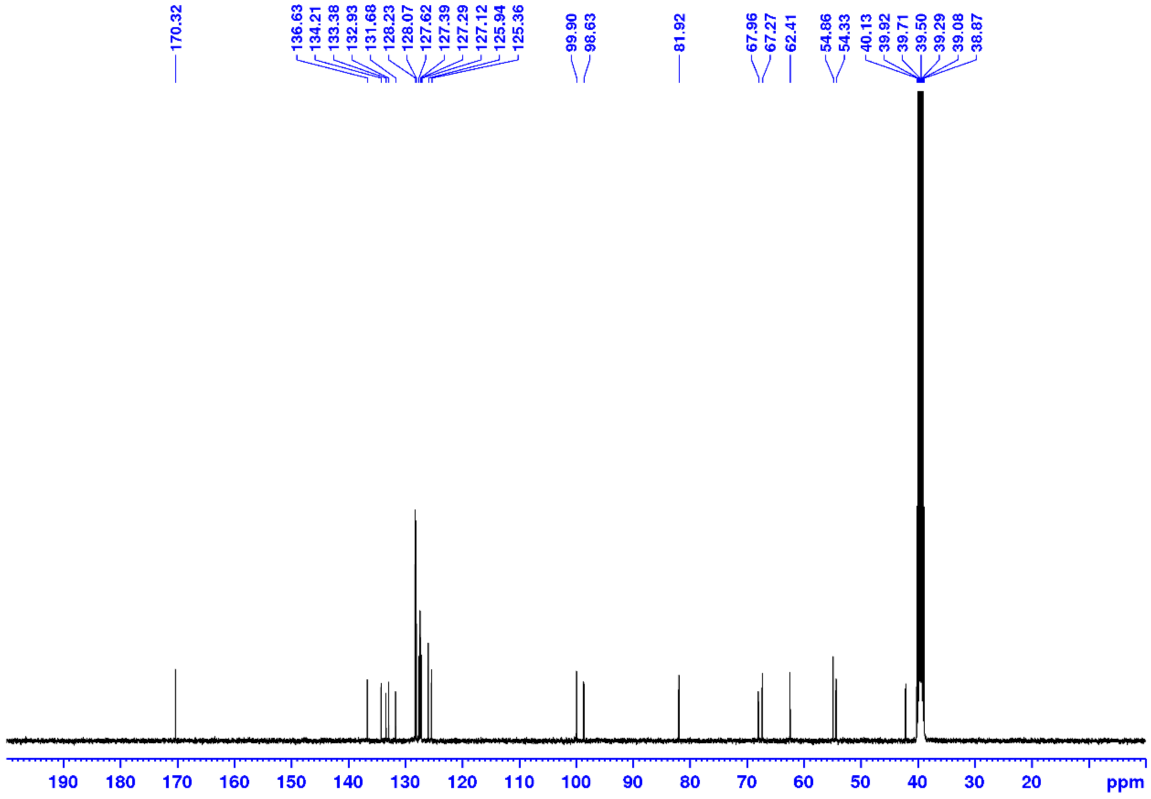

^{1}H and ^{13}C NMR spectra for compound **18** in d_6-DMSO

II. 2D NMR spectra of selected compounds

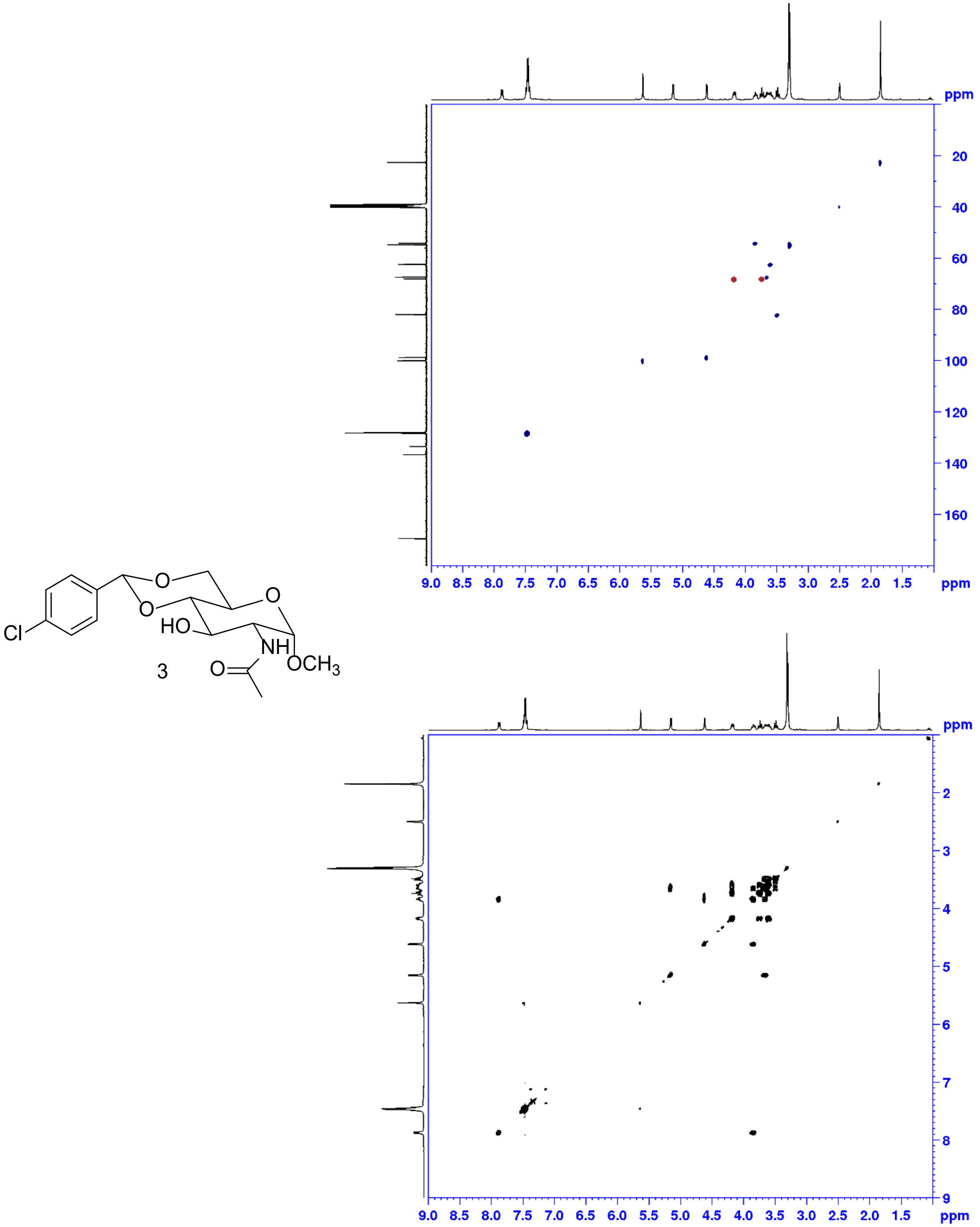

HSQC and COSY spectra of compound **3** in $CDCl_3$

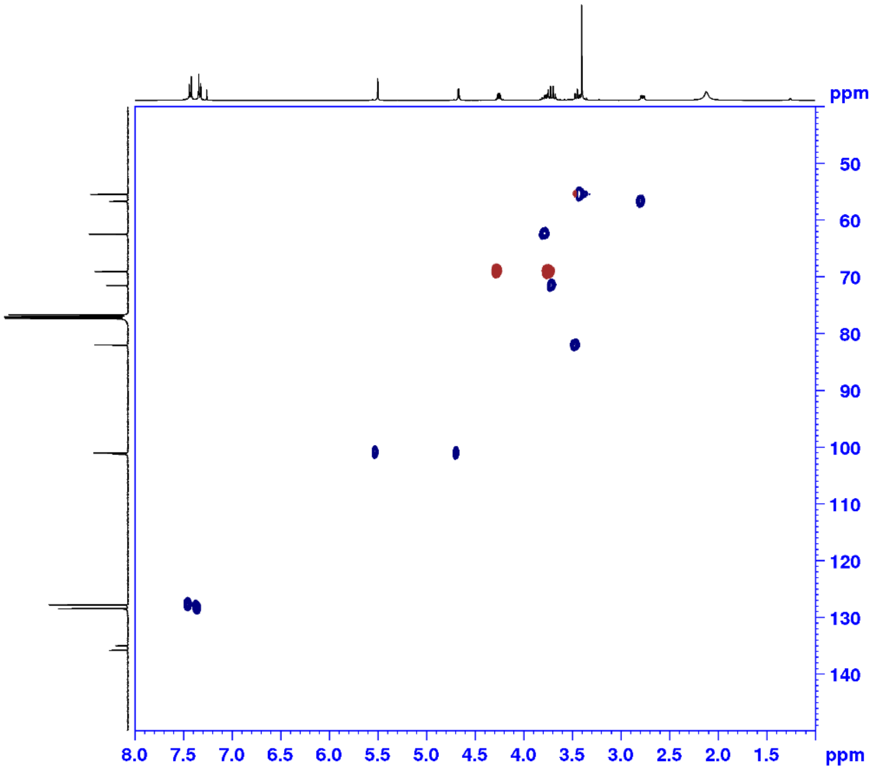

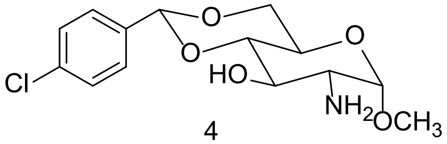

4

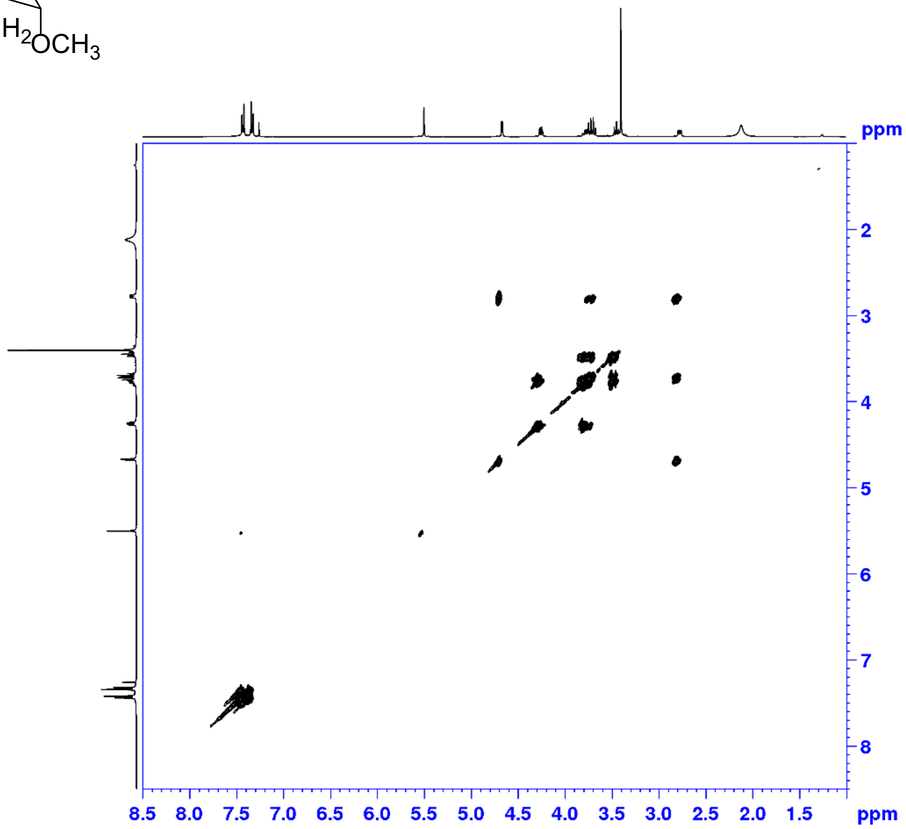

HSQC and COSY spectra of compound **4** in $CDCl_3$

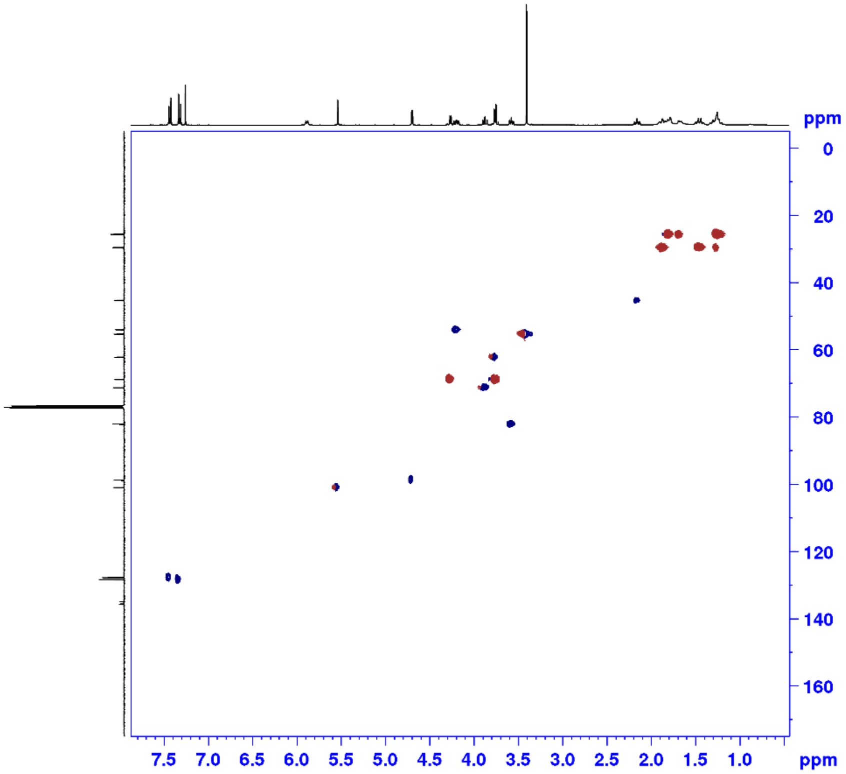

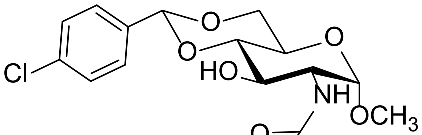

9

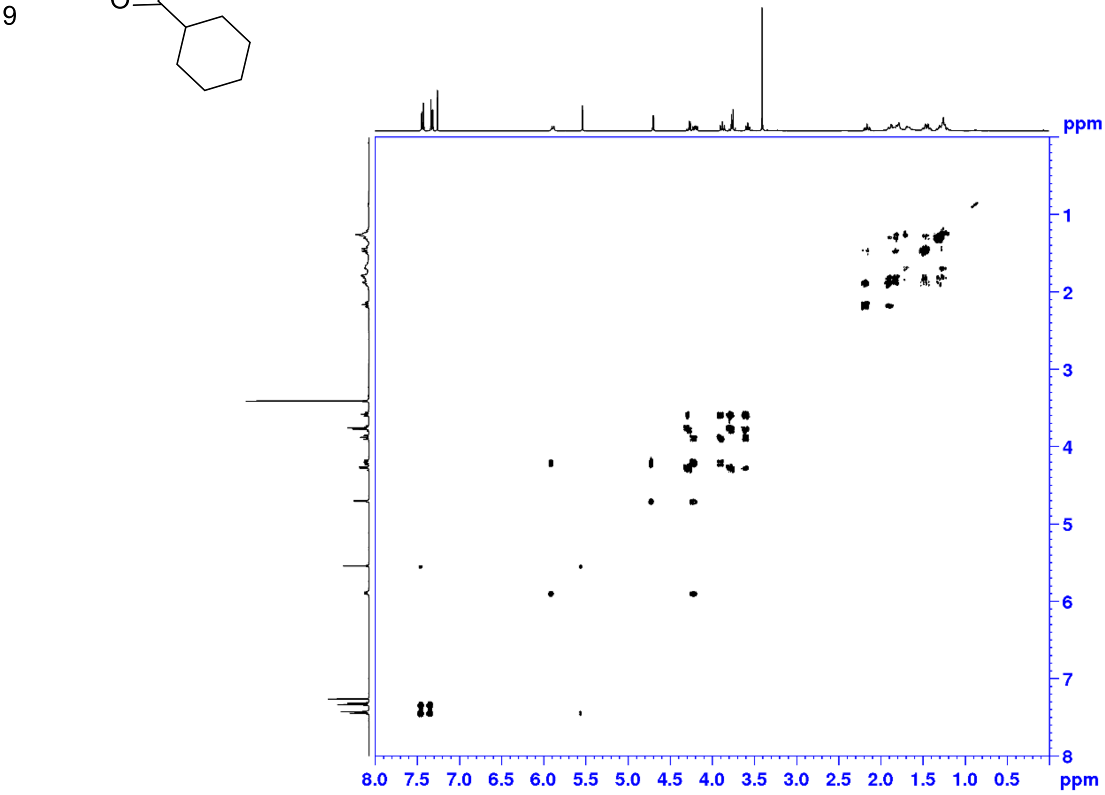

HSQC and COSY spectra for compound **9** in $CDCl_3$

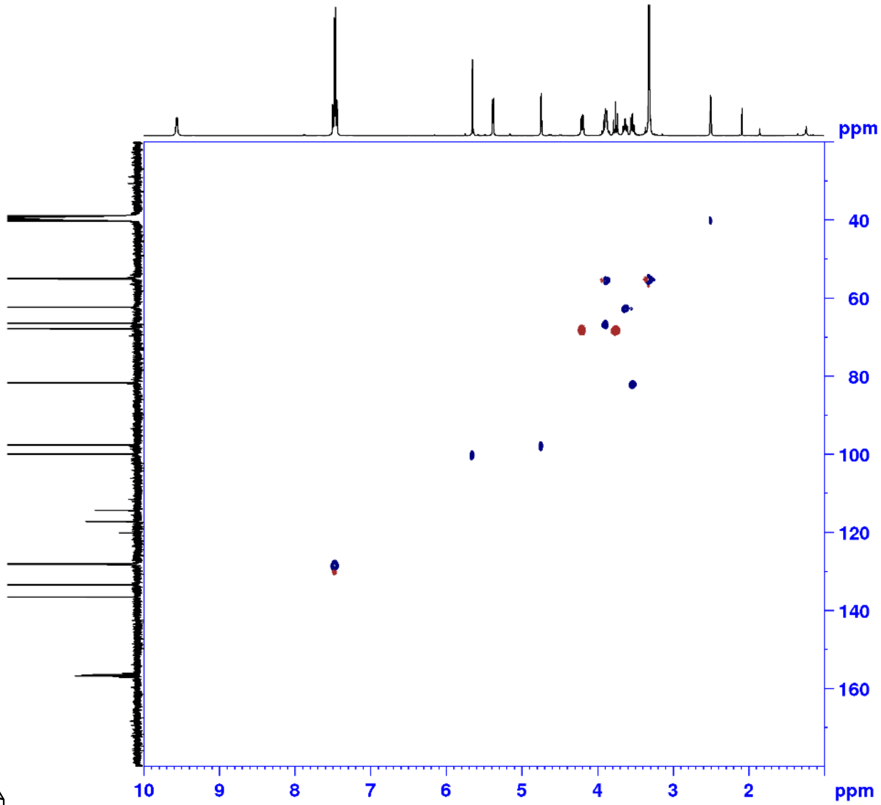

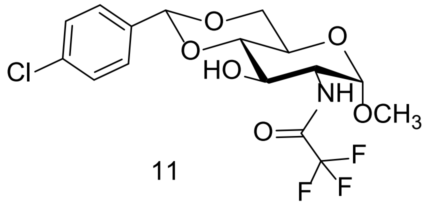

11

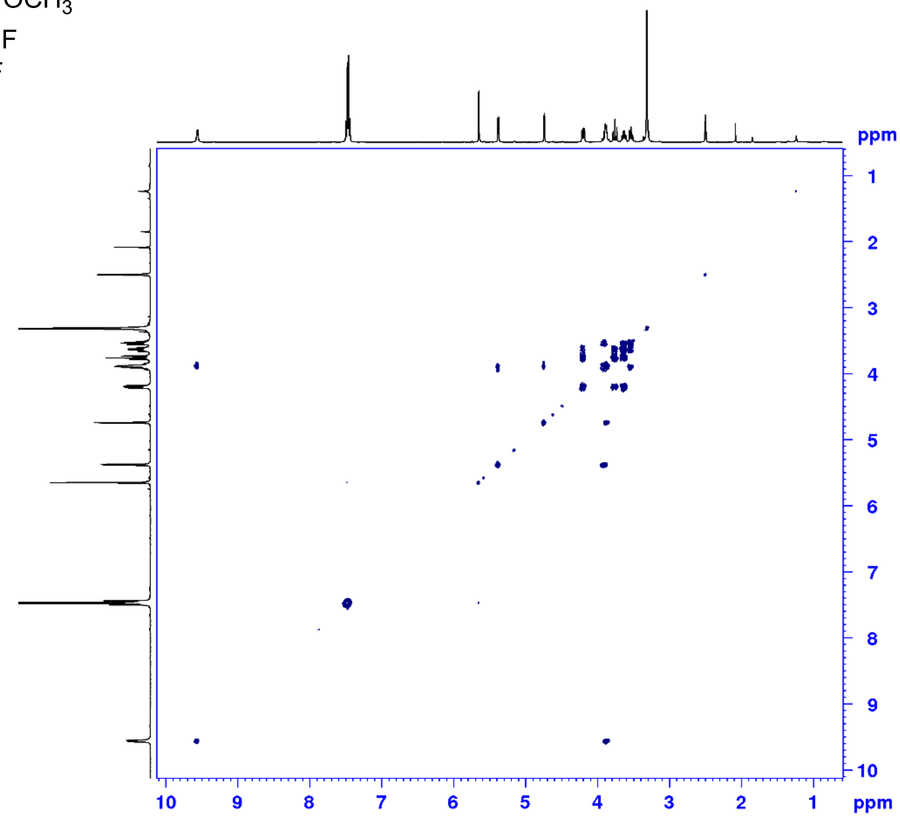

HSQC and COSY spectra for compound **11** in d_6-DMSO

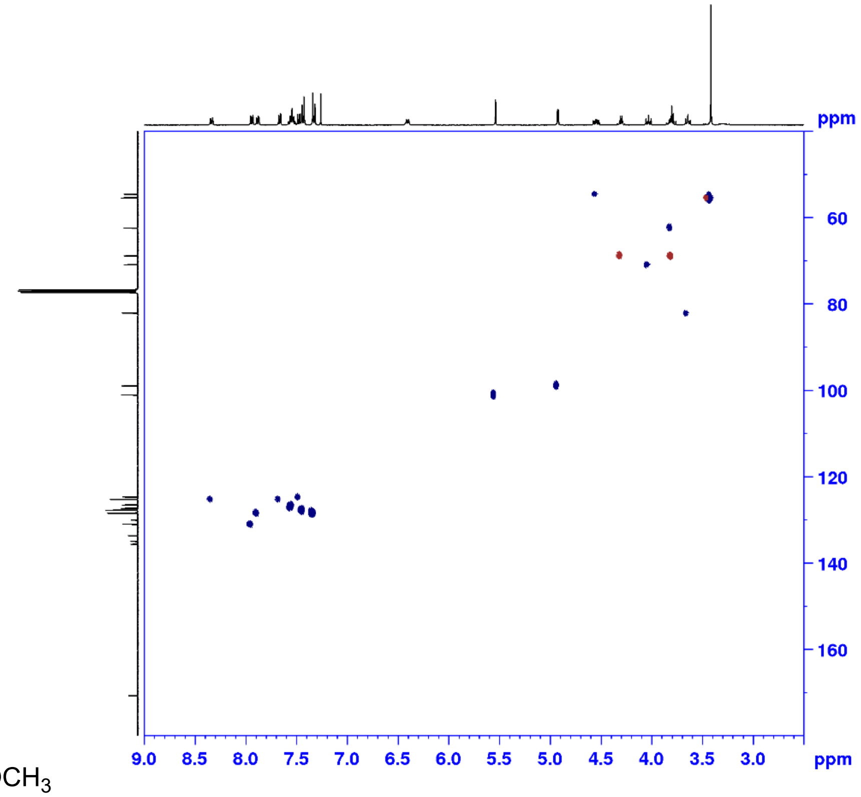

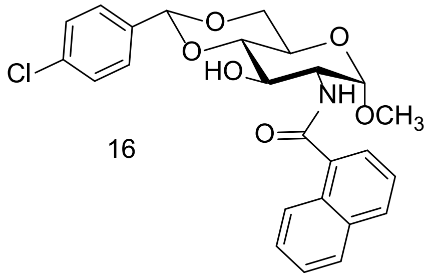

16

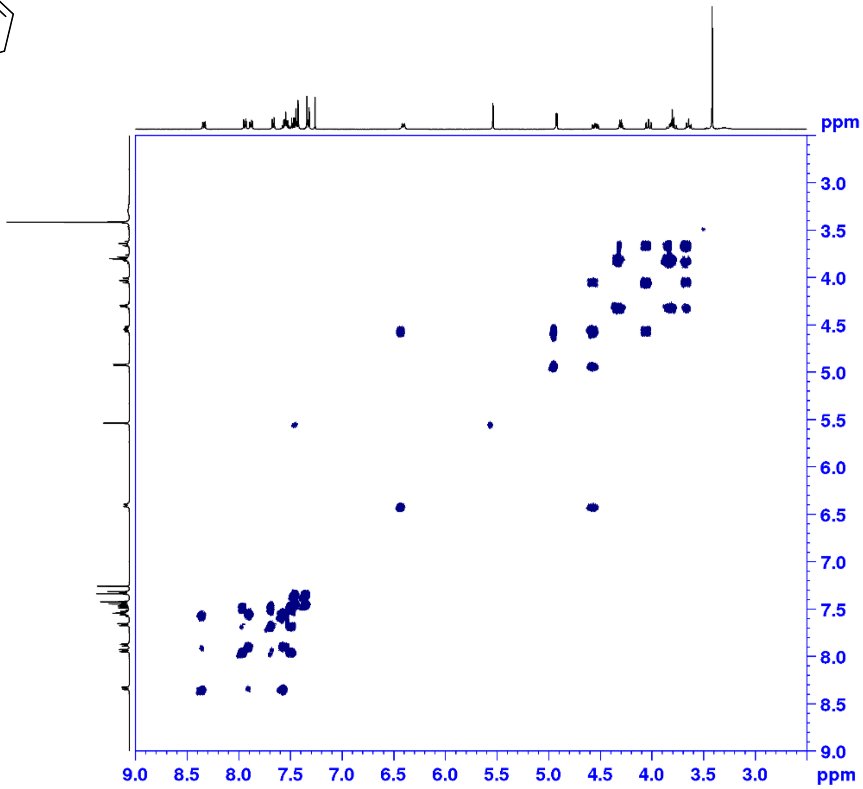

HSQC and COSY spectra for compound **16** in $CDCl_3$

III. The ^{1}H NMR spectra at different temperatures for compounds **3**, **11**, and **13**.

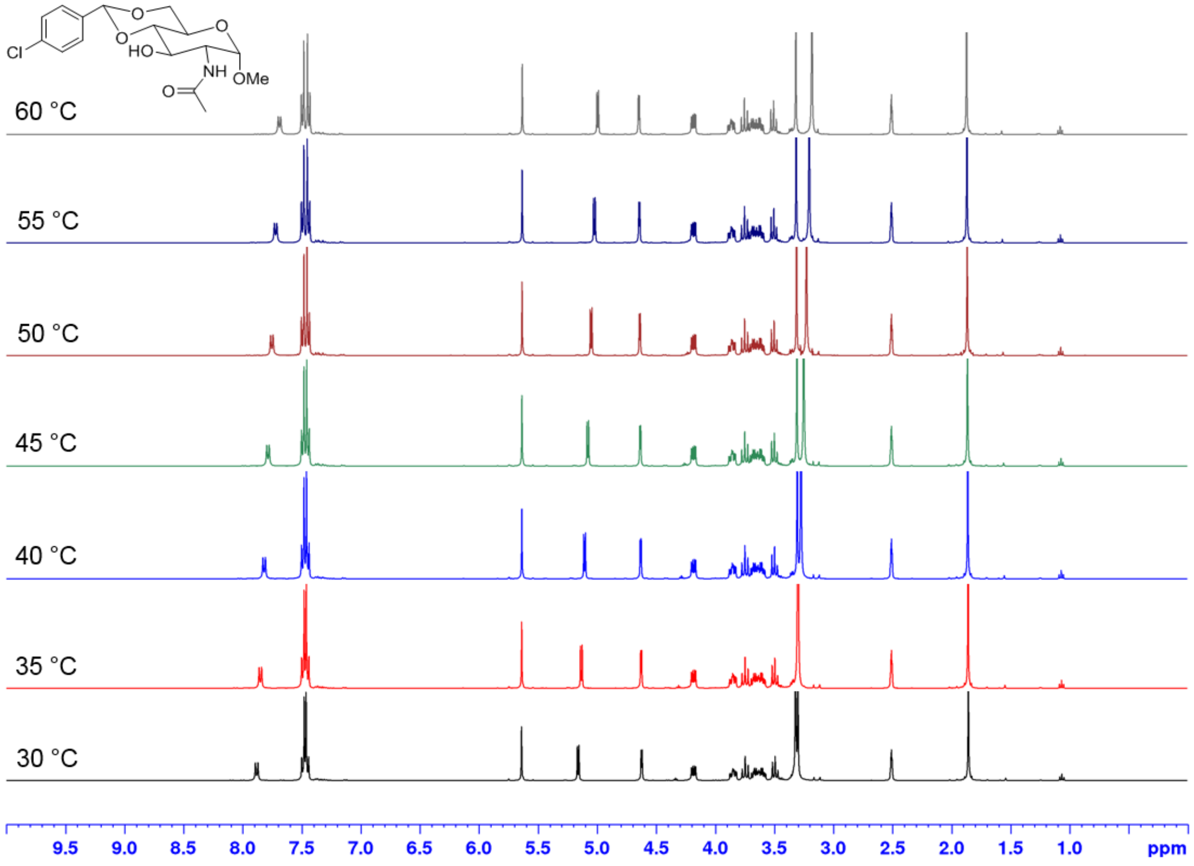

Figure S1a. Stacked ^{1}H NMR spectra of compound **3** at temperature 30-60 °C. Sample was prepared at 12.5 mg/mL in d_6-DMSO.

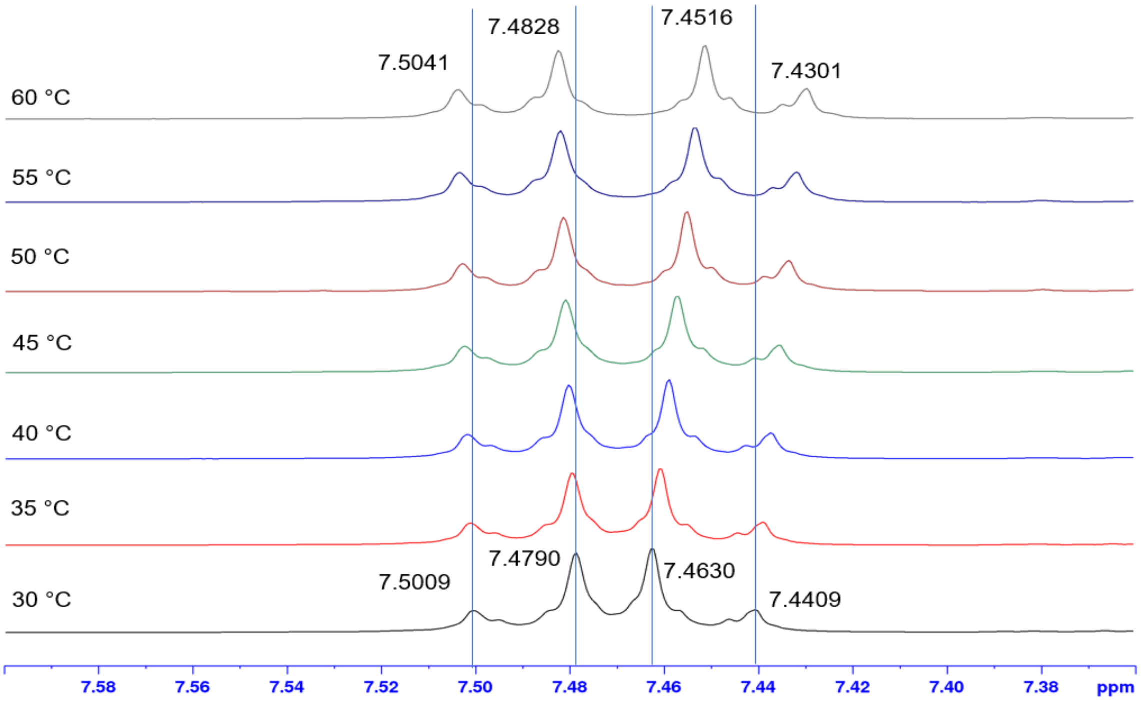

Figure S1b. Stacked ^{1}H NMR spectra of compound **3** at temperature 30-60 °C (expanded aromatic region). Sample was prepared at 12.5 mg/mL in d_6-DMSO.

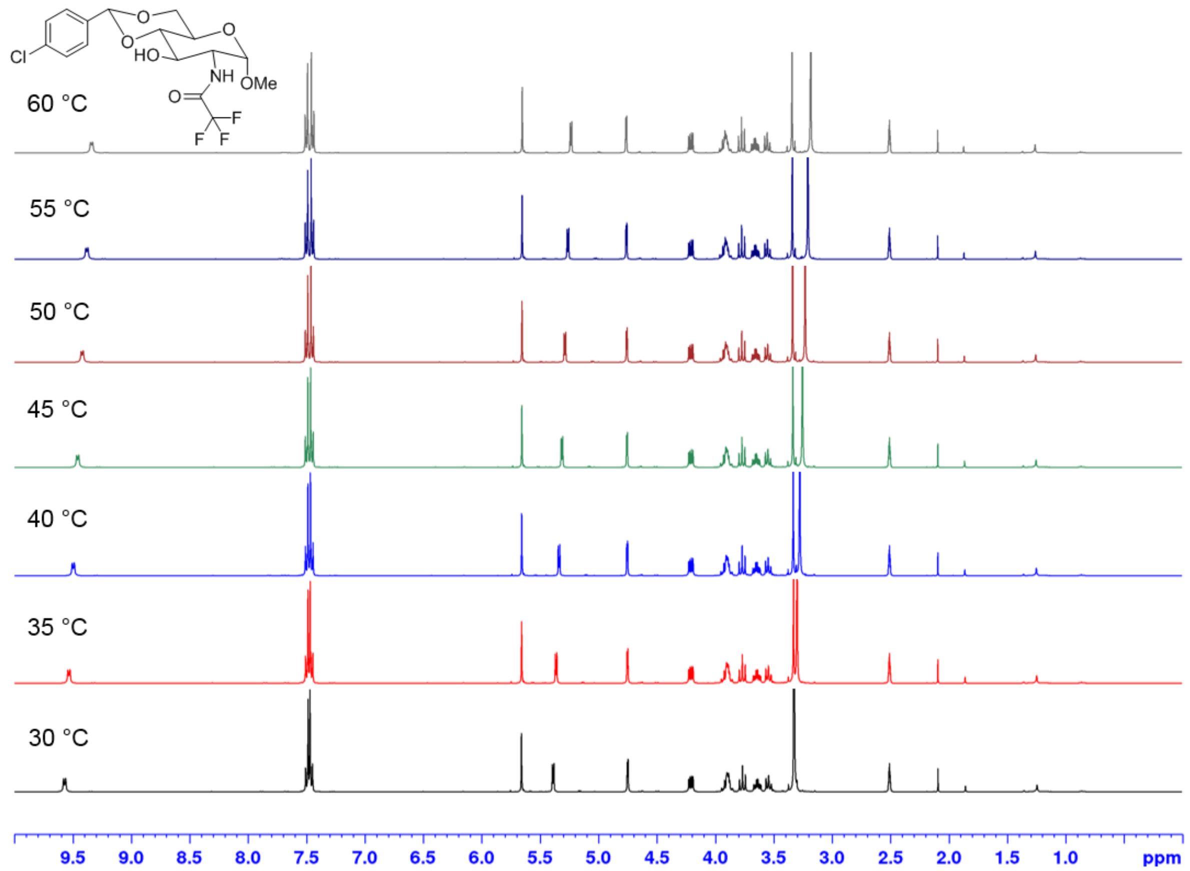

Figure S2a. Full stacked ^{1}HNMR spectra of compound **11** at temperature 30-60 °C. Sample was prepared at 12.5 mg/mL in d_6-DMSO.

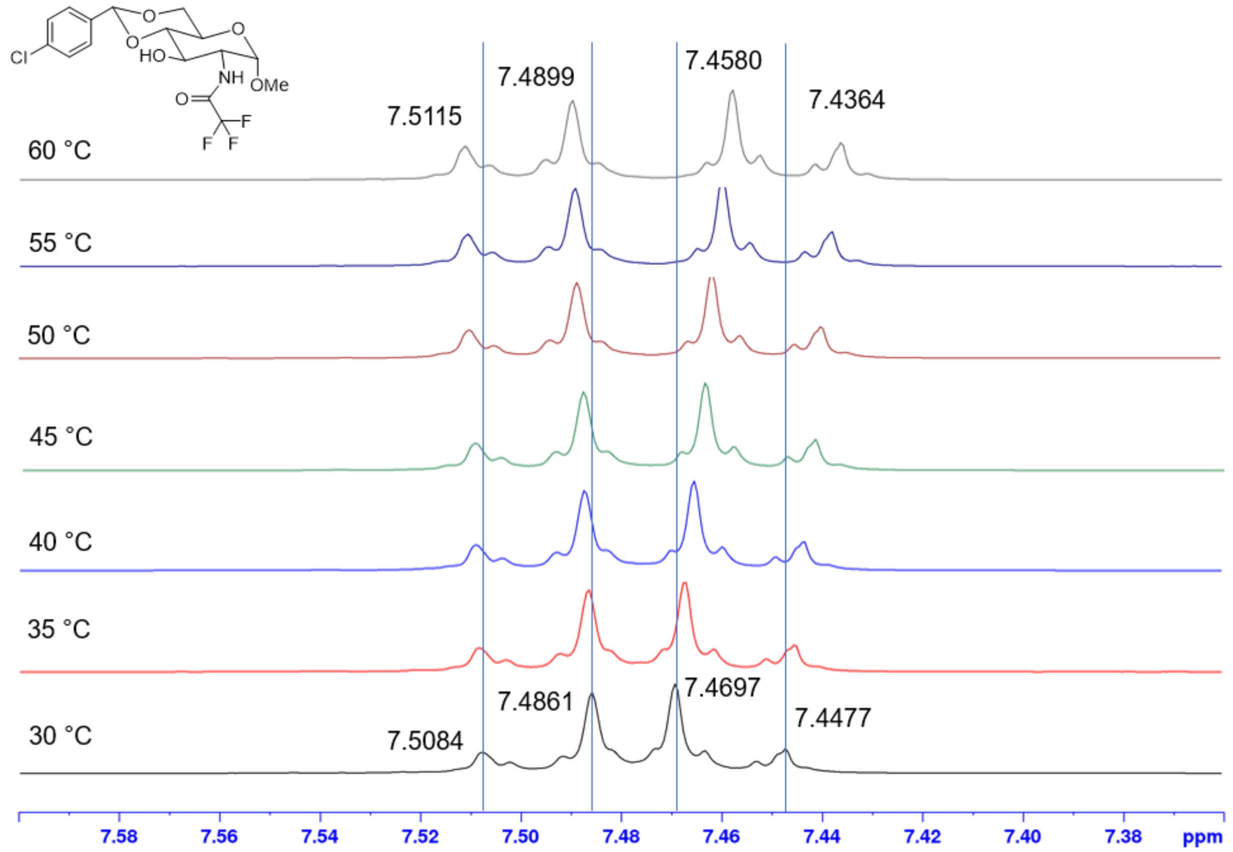

Figure S2b. Stacked ^{1}HNMR spectra of compound **11** at temperature 30-60 °C, expanded to examine the aromatic region. Sample was prepared at 12.5 mg/mL in d_6-DMSO.

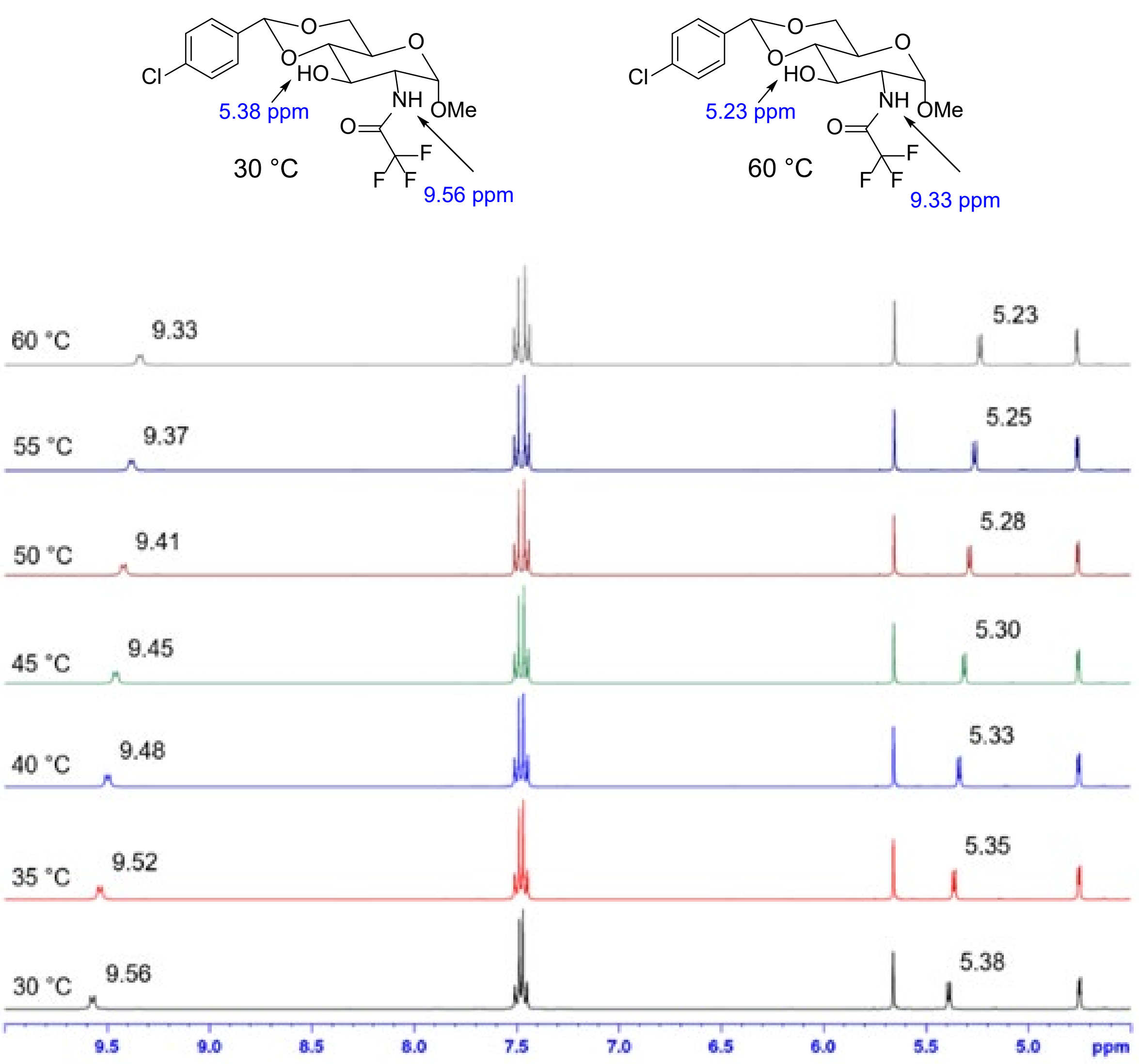

Figure S2c. Stacked ^{1}H NMR spectra of compound **11** at temperature 30-60 °C from 4.5 ppm to 10.0 ppm. Sample was prepared at 12.5 mg/mL in d_6-DMSO.

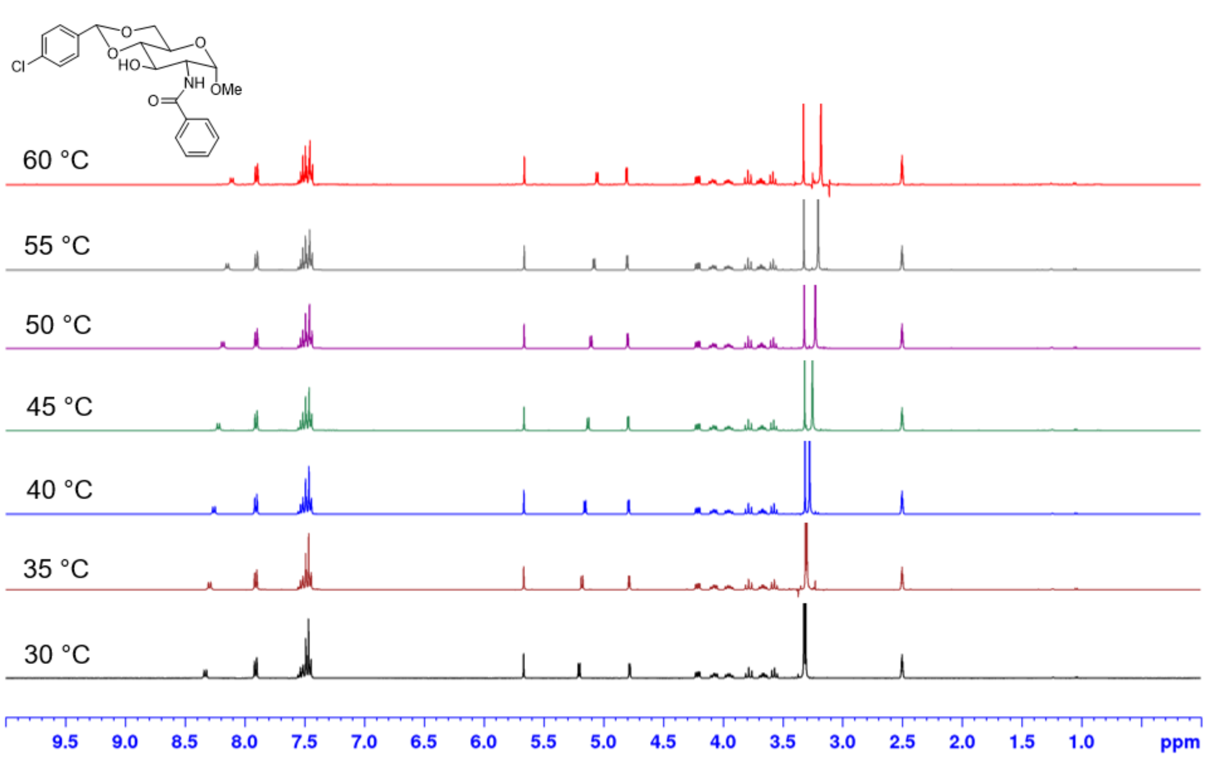

Figure S3a. Full stacked ^{1}H NMR spectra of compound **13** at 30-60 °C. Sample was prepared at 10 mg/mL in d_6-DMSO.

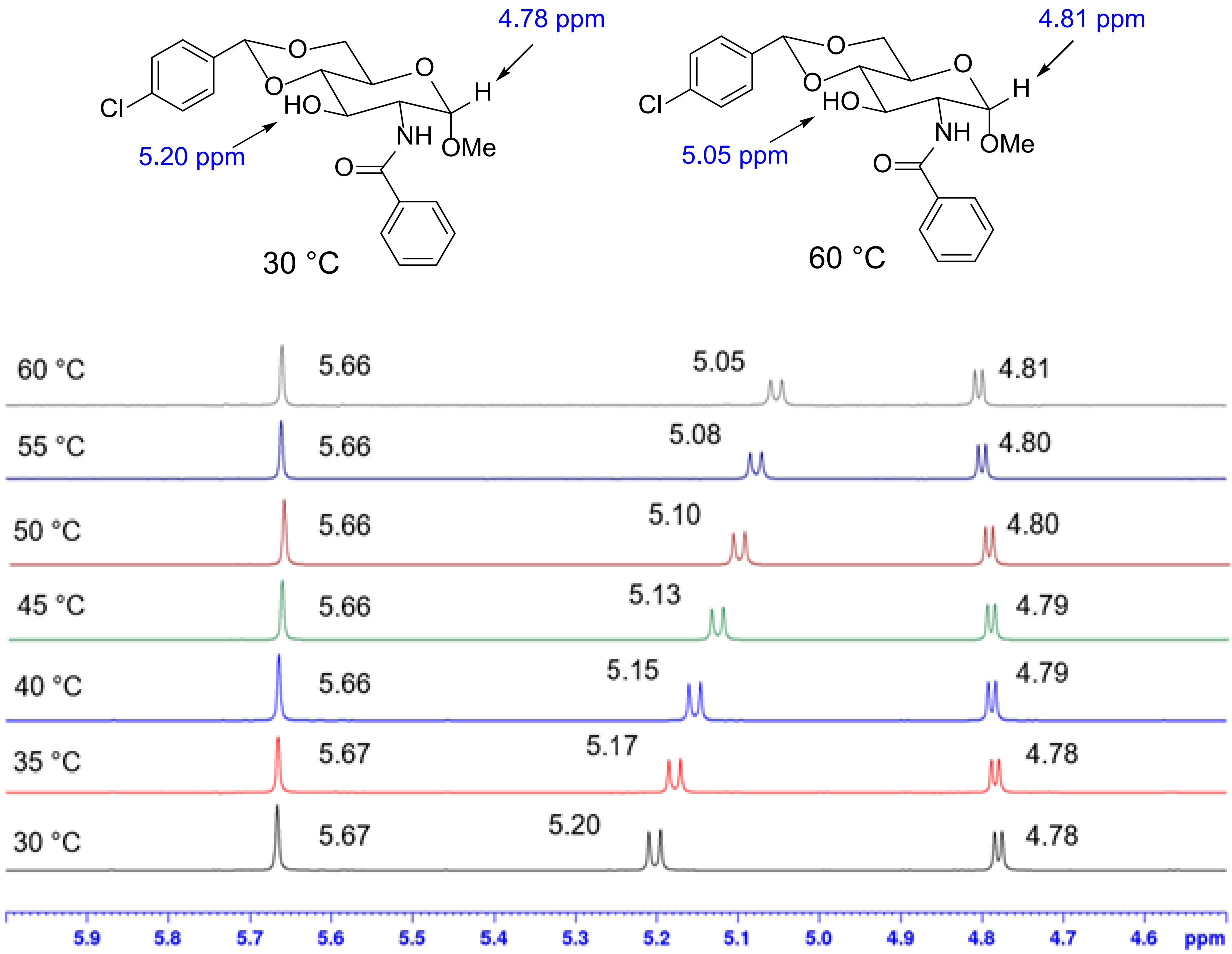

Figure S3b. ^{1}H NMR spectra of compound **13** at 30-60 °C from 4.5 ppm to .0 ppm. Sample was prepared at 10 mg/mL in d$_6$-DMSO.

IV. Rheology properties of selected gels with MGCs under 1 mg/mL

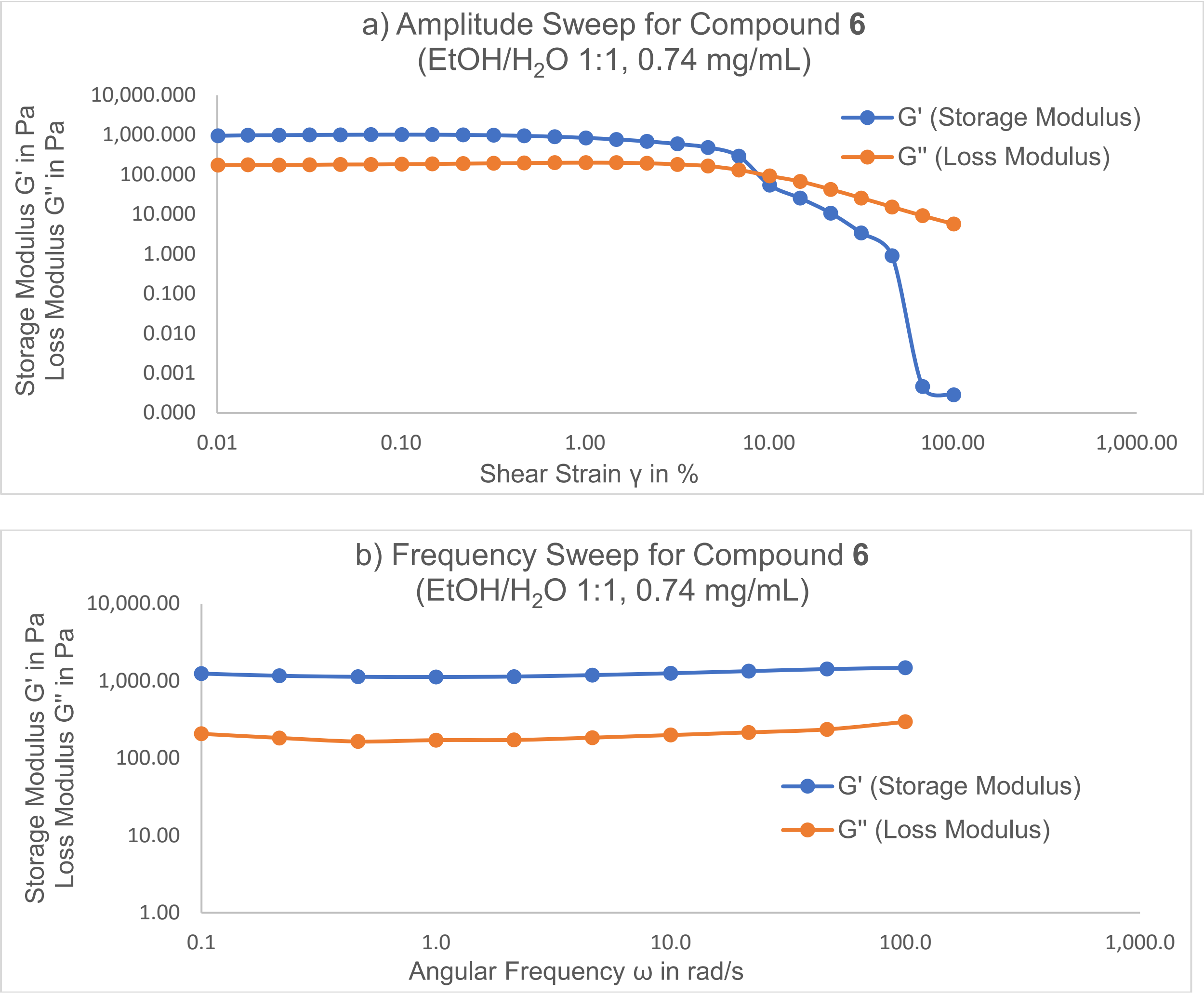

Figure S4-1. Amplitude sweep (a) and frequency sweep (b) for the EtOH/H_2O 1:1 gel formed by compound **6** at 0.74 mg/mL.

ω [rad/s]	G' (Storage Modulus)	G" (Loss Modulus)	G'/G"
100.0	1481.6	295.8	5.0
46.4	1425.3	236.4	6.0
21.5	1340.9	216.0	6.2
10.0	1259.2	199.7	6.3
4.6	1191.6	184.6	6.5
2.2	1141.1	172.4	6.6
1.0	1126.7	171.6	6.6
0.5	1132.7	164.7	6.9
0.2	1167.4	183.3	6.4
0.1	1246.2	206.9	6.0

Table S1a. Frequency sweep data for the gel in Figure S4-1

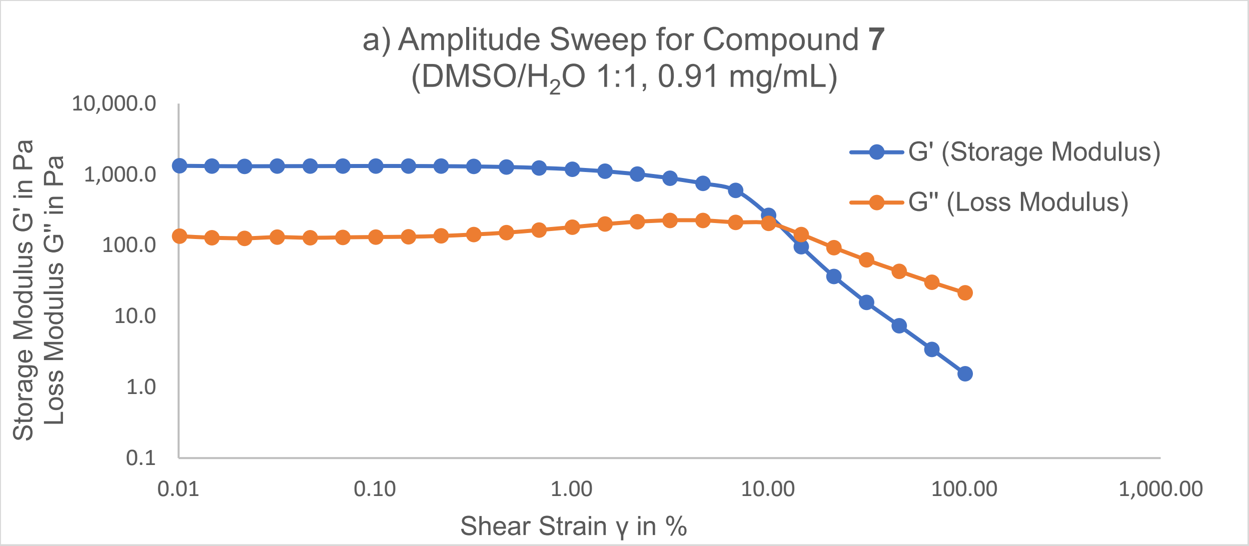

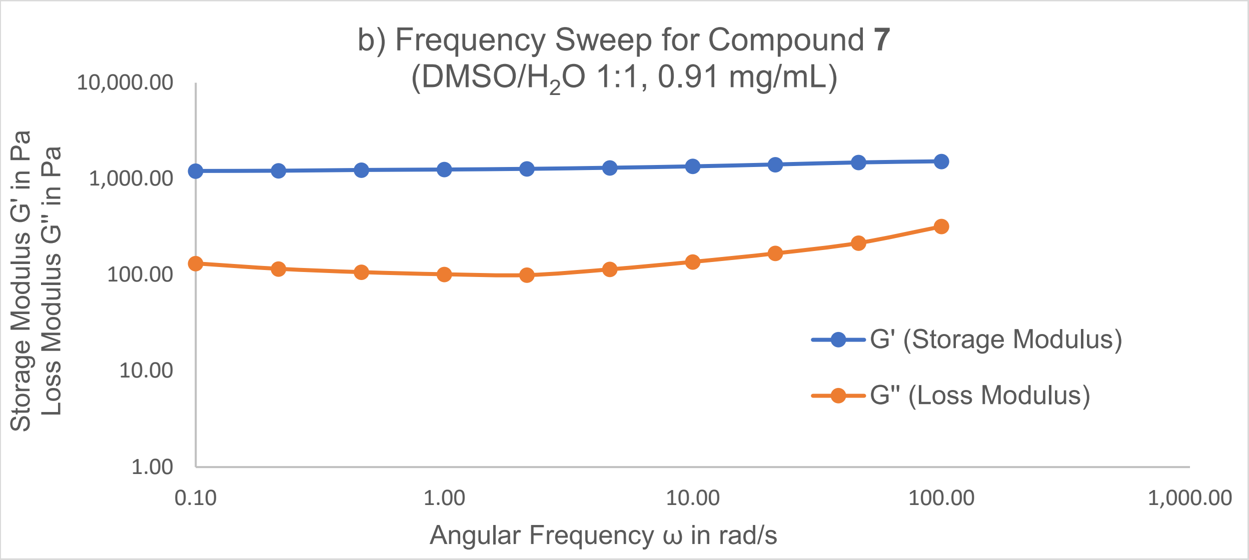

Figure S4-2. Amplitude sweep and frequency sweep for the DMSO/H_2O 1:1 gel formed by compound **7** at 0.91 mg/mL.

ω [rad/s]	G' (Storage Modulus)	G" (Loss Modulus)	G'/G"
100.0	1521.9	318.7	4.8
46.4	1481.4	214.8	6.9
21.5	1410.1	167.5	8.4
10.0	1348.4	136.7	9.9
4.6	1304.0	114.2	11.4
2.2	1269.8	99.6	12.8
1.0	1249.4	101.6	12.3
0.5	1236.3	106.9	11.6
0.2	1214.2	115.8	10.5
0.1	1207.5	131.7	9.2

Table S1b. Frequency sweep data for the gel in Figure S4-2.

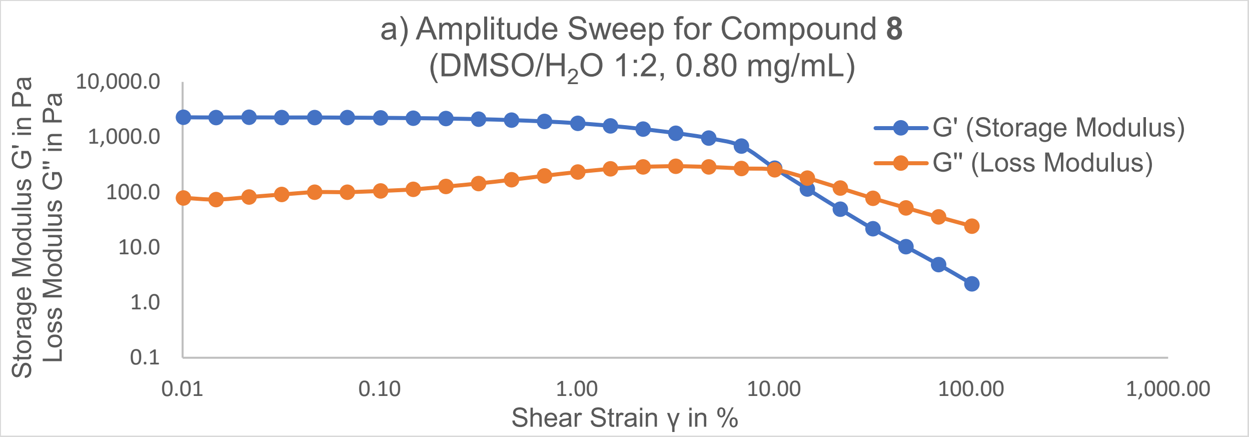

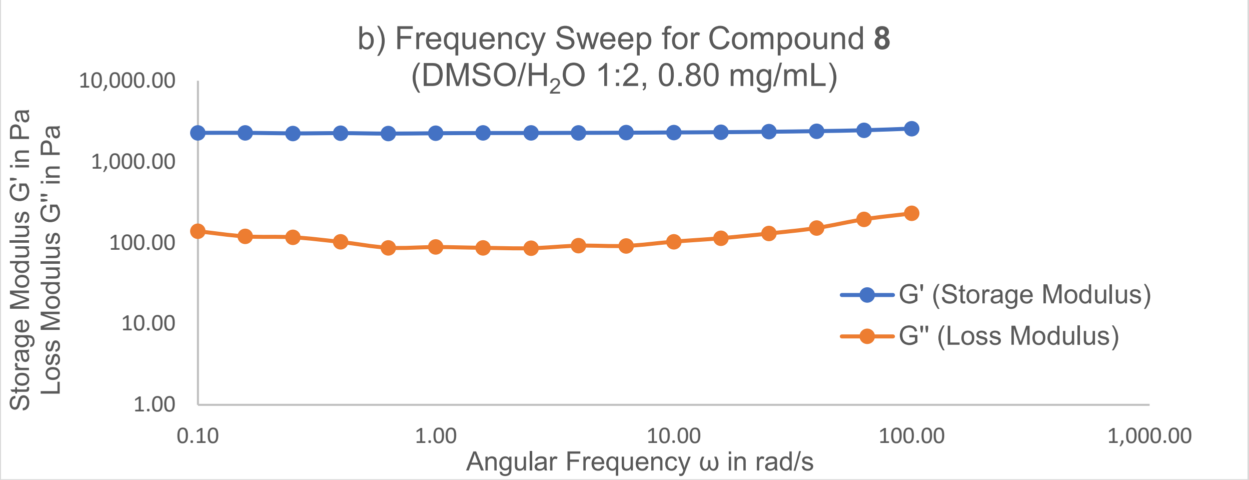

Figure S4-3. Amplitude sweep and frequency sweep for the DMSO/H_2O 1:2 gel formed by compound **8** at 0.80 mg/mL.

ω [rad/s]	G' (Storage Modulus)	G" (Loss Modulus)	G'/G"
100.0	2566.1	230.6	11.1
63.1	2449.0	195.2	12.5
39.8	2387.5	152.3	15.7
25.1	2345.9	130.2	18.0
15.8	2318.3	114.0	20.3
10.0	2300.1	103.3	22.3
6.3	2283.8	91.6	24.9
4.0	2274.1	92.2	24.7
2.5	2265.6	85.7	26.4
1.6	2262.1	86.7	26.1
1.0	2249.5	88.6	25.4
0.6	2226.8	86.9	25.6
0.4	2256.5	102.4	22.0
0.3	2240.2	117.1	19.1
0.2	2277.2	119.9	19.0
0.1	2273.5	138.9	16.4

Table S1c. Frequency sweep data for the gel in Figure S4-3.

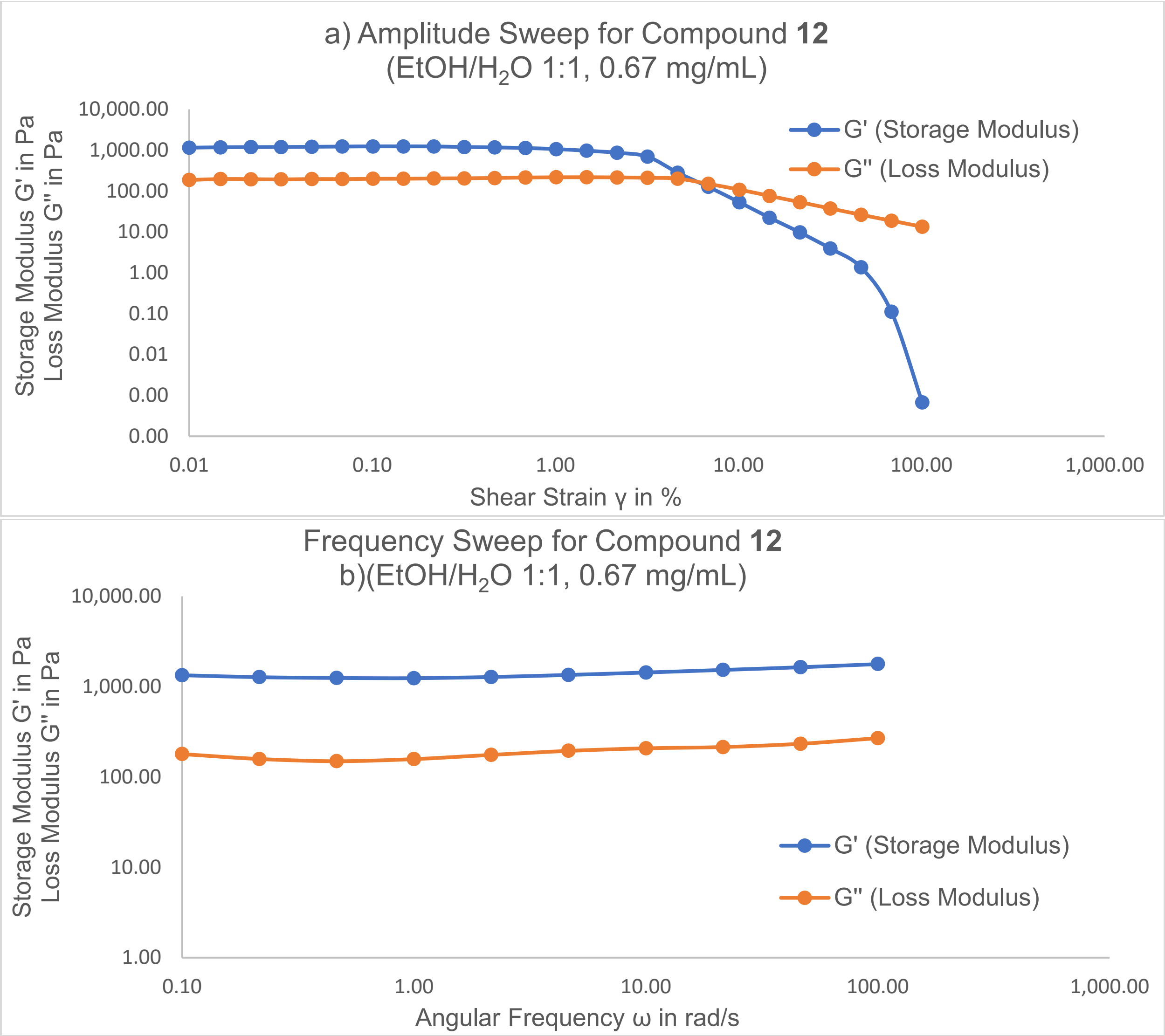

Figure S4-4. Amplitude sweep and frequency sweep for the EtOH/H_2O 1:1 gel formed by compound **12** at 0.67 mg/mL.

ω [rad/s]	G' (Storage Modulus)	G" (Loss Modulus)	G'/G"
100.0	1776.8	269.1	6.6
46.4	1639.9	233.0	7.0
21.5	1531.4	214.6	7.1
10.0	1434.2	207.7	6.9
4.6	1348.1	194.5	6.9
2.2	1278.9	175.6	7.3
1.0	1241.6	157.7	7.9
0.5	1247.8	149.6	8.3
0.2	1272.8	158.2	8.0
0.1	1337.9	179.2	7.5

Table S1d. Frequency sweep data for the gel in Figure S4-4.

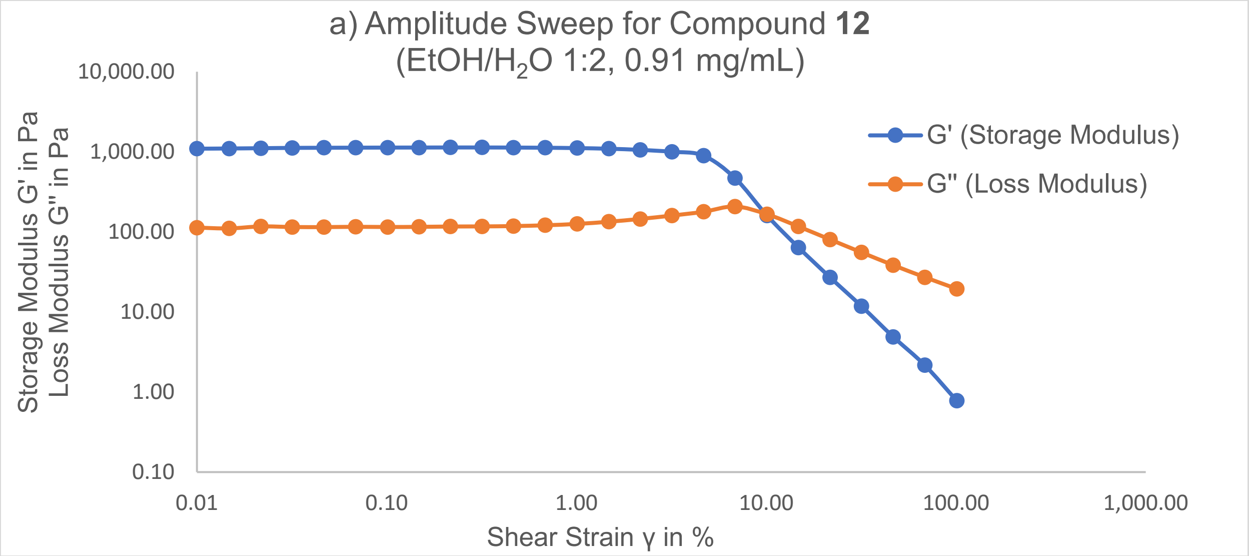

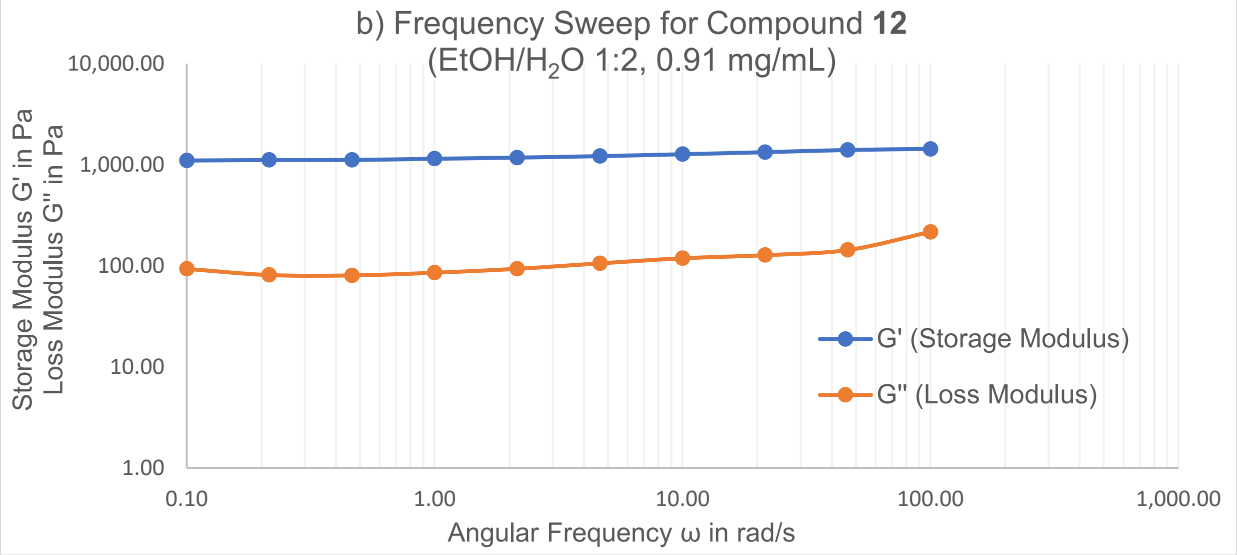

Figure S4-5. Amplitude sweep and frequency sweep for the EtOH/H_2O 1:2 gel formed by compound **12** at 0.91 mg/mL.

ω [rad/s]	G' (Storage Modulus)	G" (Loss Modulus)	G'/G"
100.0	1434.0	216.7	6.6
46.4	1399.3	143.2	9.8
21.5	1330.6	127.4	10.4
10.0	1270.0	118.7	10.7
4.6	1216.2	105.7	11.5
2.2	1176.2	93.4	12.6
1.0	1145.8	85.4	13.4
0.5	1115.7	80.3	13.9
0.2	1112.7	81.0	13.7
0.1	1100.5	93.2	11.8

Table S1e. Frequency sweep data for the gel in Figure S4-5

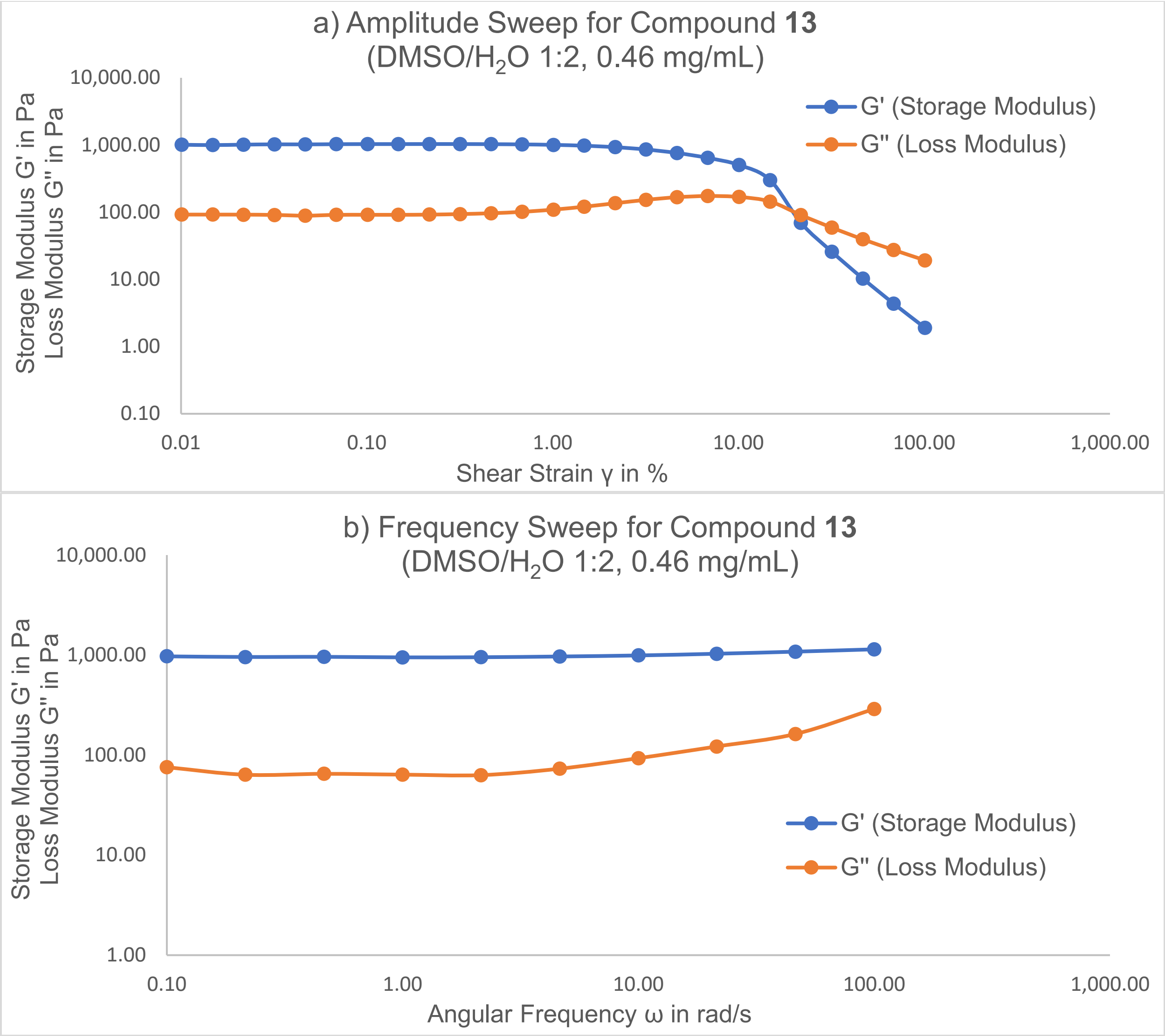

Figure S4-6. Amplitude sweep and frequency sweep for the DMSO/H_2O 1:2 gel formed by compound **13** at 0.46 mg/mL.

ω [rad/s]	G' (Storage Modulus)	G'' (Loss Modulus)	G'/G''
100.0	1141.8	289.4	3.9
46.4	1082.3	162.8	6.7
21.5	1031.7	122.1	8.5
10.0	991.9	93.2	10.6
4.6	968.4	73.2	13.2
2.2	952.0	63.1	15.1
1.0	949.0	63.8	14.9
0.5	960.5	65.0	14.8
0.2	957.3	63.6	15.0
0.1	971.4	75.7	12.8

Table S1f. Frequency sweep data for the gel in Figure S4-6

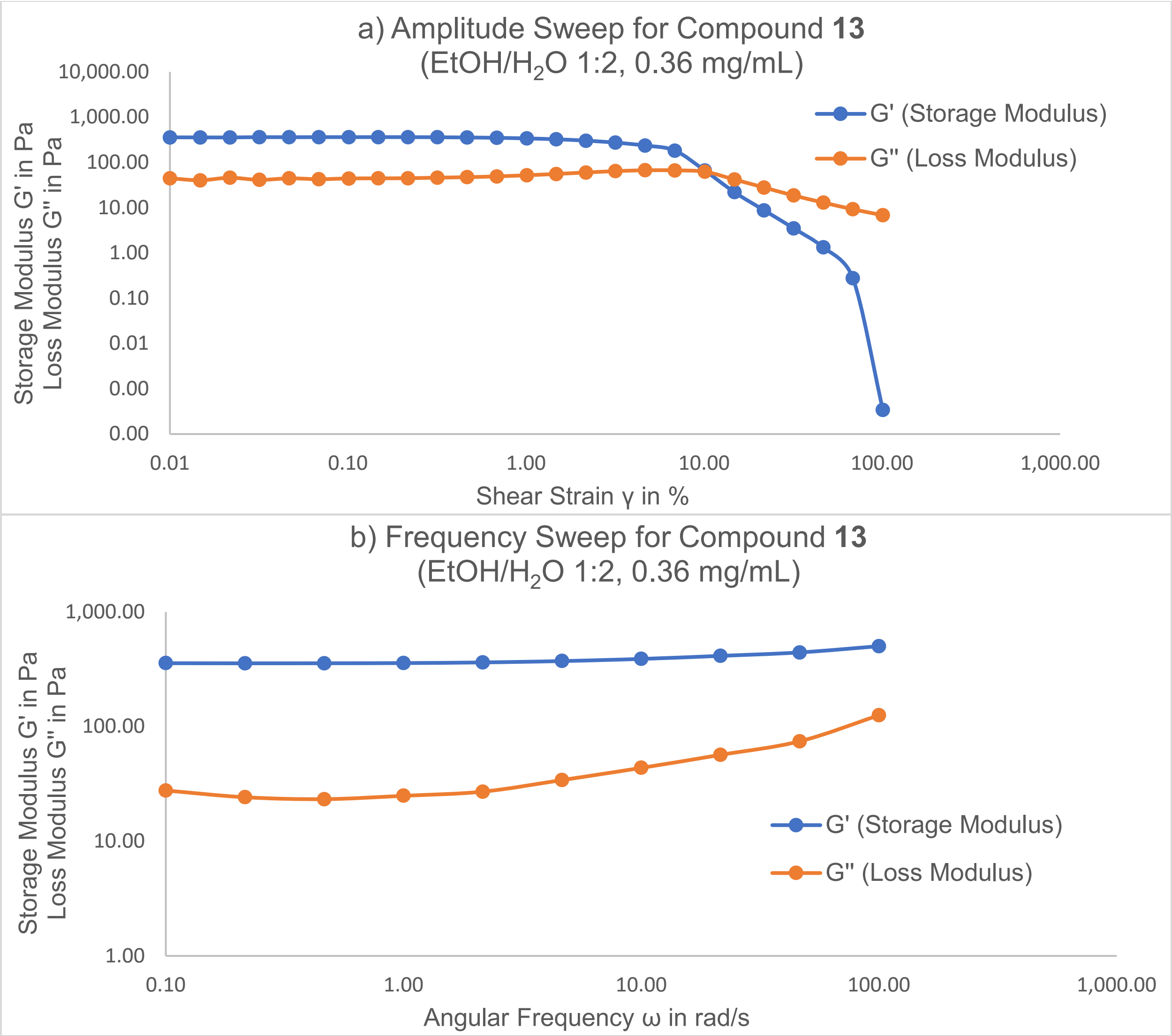

Figure S4-7. Amplitude sweep and frequency sweep for the EtOH/H_2O 1:2 gel formed by compound **13** at 0.36 mg/mL.

ω [rad/s]	G' (Storage Modulus)	G" (Loss Modulus)	G'/G"
100.0	502.0	125.4	4.0
46.4	443.0	74.4	6.0
21.5	413.4	56.6	7.3
10.0	388.8	43.6	8.9
4.6	372.4	34.1	10.9
2.2	362.5	27.1	13.4
1.0	357.7	24.9	14.4
0.5	356.4	23.3	15.3
0.2	355.9	24.2	14.7
0.1	356.8	27.7	12.9

Table S1g. Frequency sweep data for the gel in Figure S4-7

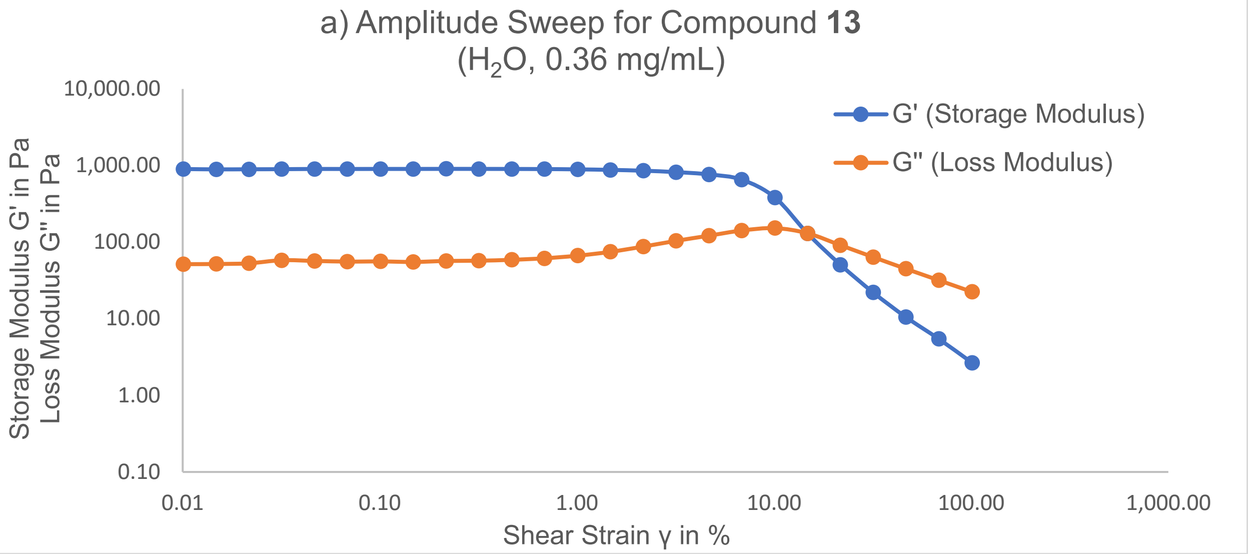

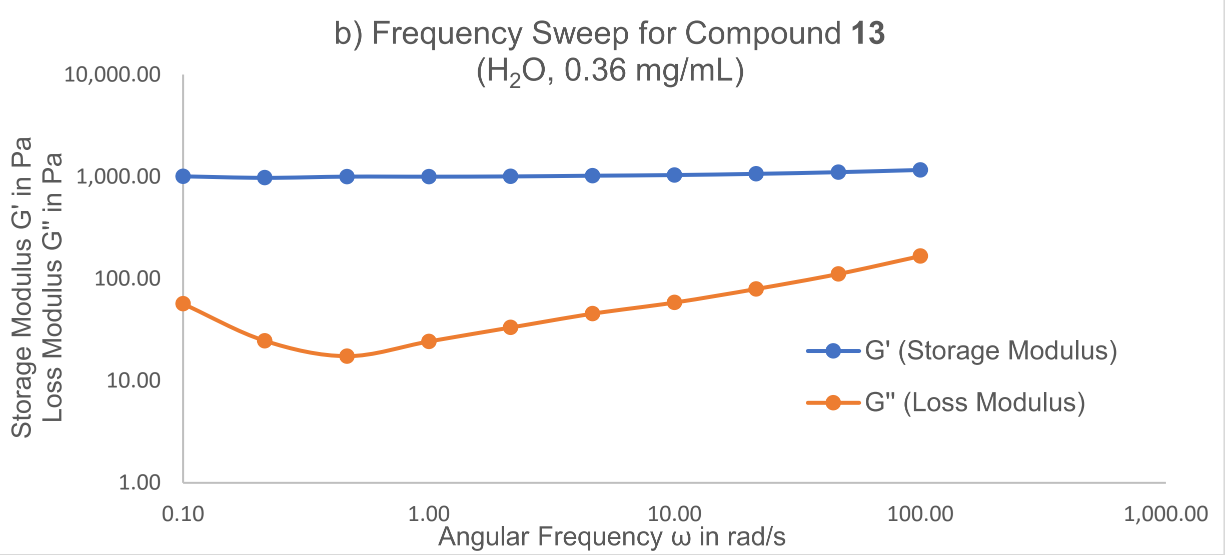

Figure S4-8. Amplitude sweep, frequency sweep, and rheological data table for the hydrogel formed by compound **13** at 0.36 mg/mL.

ω [rad/s]	G' (Storage Modulus)	G" (Loss Modulus)	G'/G"
100.0	1162.0	166.2	7.0
46.4	1104.9	111.1	9.9
21.5	1064.2	79.2	13.4
10.0	1035.7	58.4	17.7
4.6	1020.4	45.4	22.5
2.2	1004.2	33.2	30.2
1.0	998.4	24.3	41.2
0.5	999.6	17.4	57.4
0.2	972.4	24.6	39.5
0.1	1005.9	56.7	17.8

Table S1h. Frequency sweep data for the gel in Figure S4-8

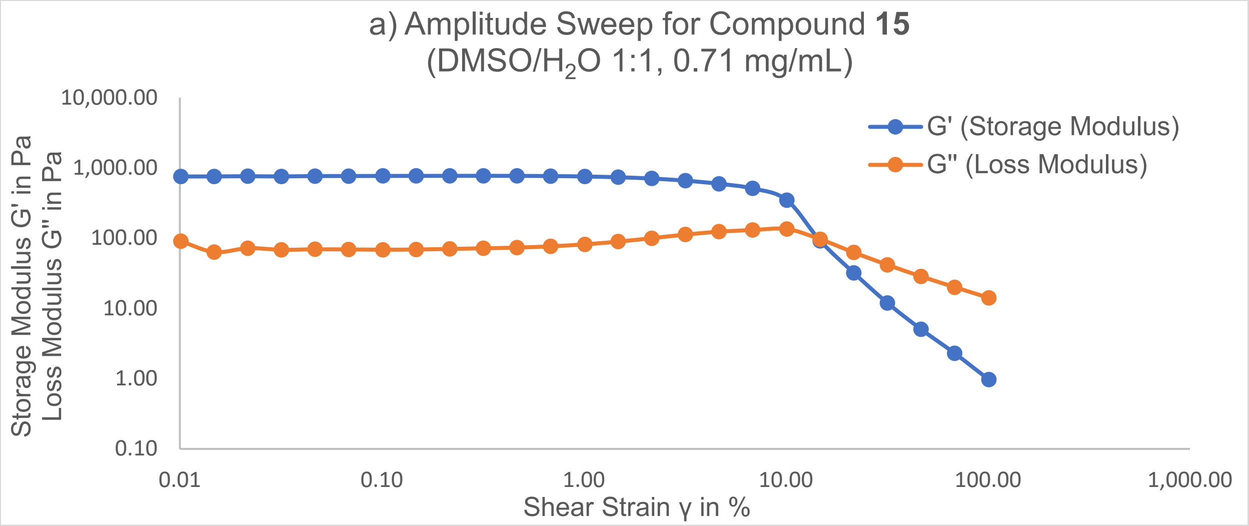

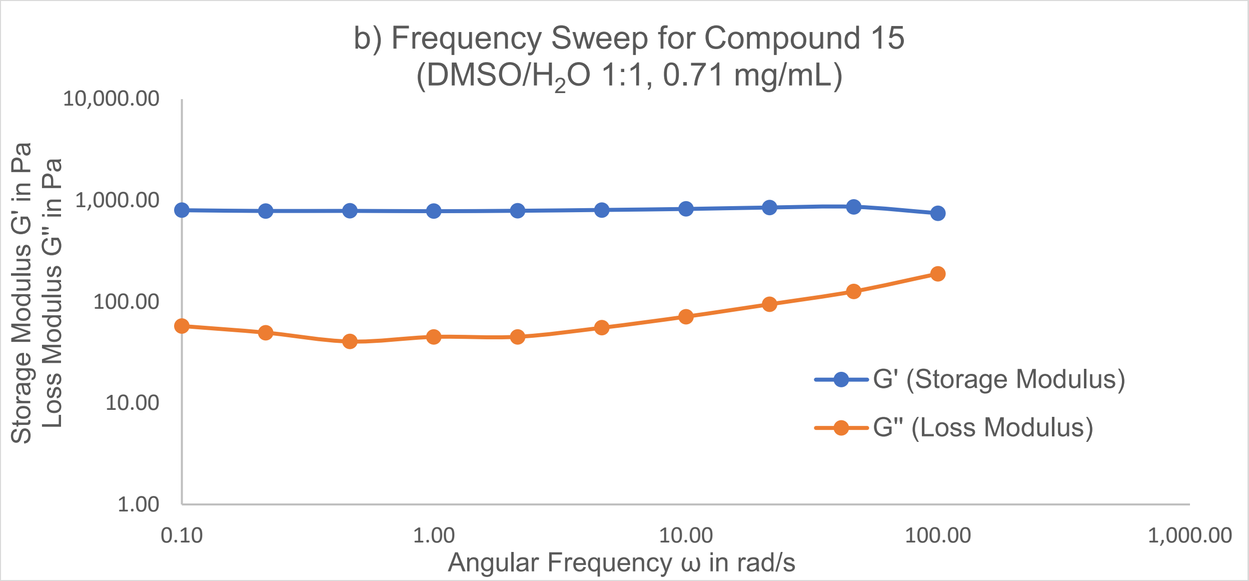

Figure S4-9. Amplitude sweep and frequency sweep for the DMSO/H_2O 1:1 gel formed by compound **15** at 0.71 mg/mL.

ω [rad/s]	G' (Storage Modulus)	G" (Loss Modulus)	G'/G"
100.0	748.5	189.1	4.0
46.4	865.8	126.6	6.8
21.5	851.9	94.5	9.0
10.0	824.0	71.3	11.6
4.6	805.9	55.4	14.5
2.2	790.8	45.1	17.5
1.0	783.3	45.0	17.4
0.5	788.8	40.5	19.5
0.2	787.0	49.6	15.9
0.1	800.8	57.6	13.9

Table S1i. Rheological data for the DMSO/H_2O 1:1 gel formed by compound **15** at 0.71 mg/mL.

V. Gel extrusion experiments and rheology properties

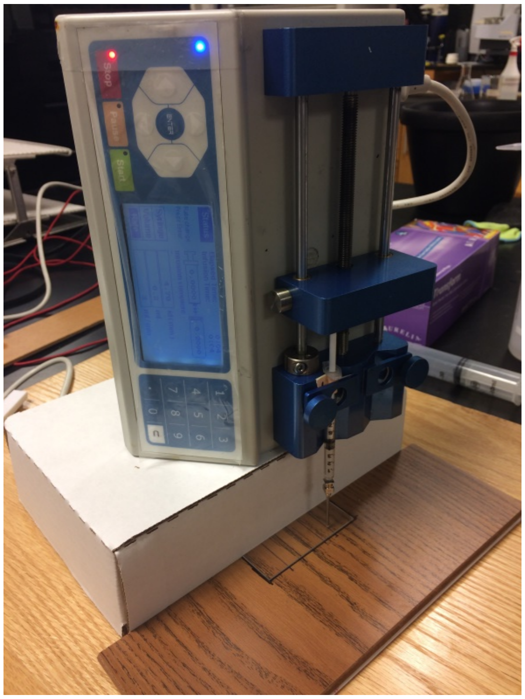

Figure S5-1

Figure S5-1 shows the set up used for the extrusion studies. The syringe pump is inverted and set up with a 1 mL syringe equipped with a 14-gauge blunt tipped needle. Directly underneath this set-up was a microscope slide on top of a laminate board, which was manually pushed about 4 cm during each experiment. Graduation markings drawn on the laminate board were used to estimate the distance for each trial and help keep length of the extruded gel lines somewhat consistent. The clearance between the tip of the blunt tipped needle and the microscope slide is ≈2.2 mm, measured via a micrometer. For each trial, 200 µL of gel was extruded over 4 cm.

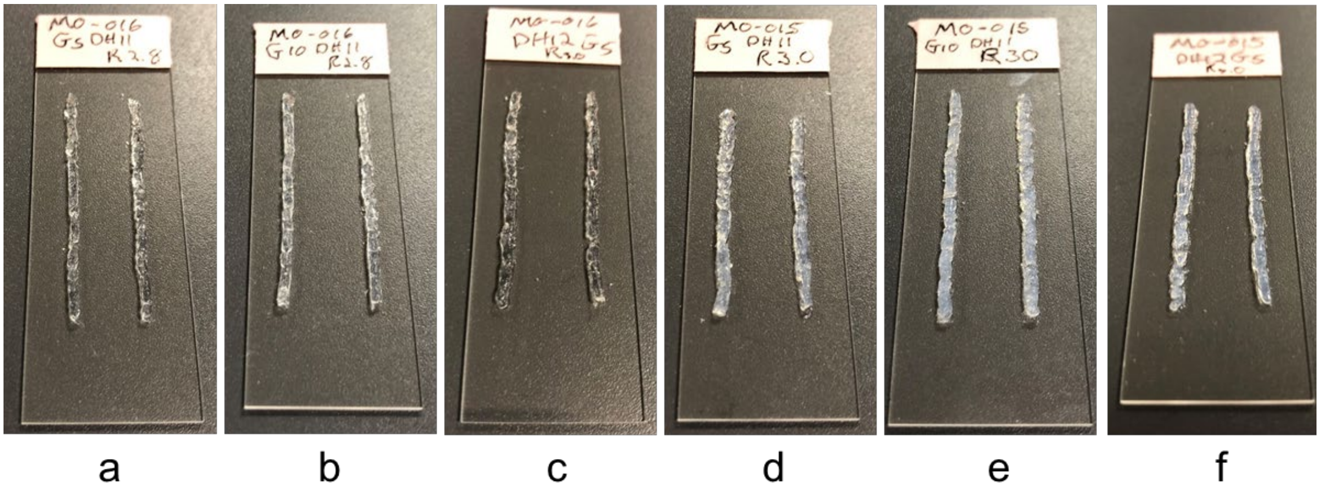

Figure S5-2. Extruded gels of compound **11** (a-c) and compound **13** (d-f). The solvents for the gels and concentrations of the gelators are: (a, d) DMSO/H_2O 1:1 at 5.0 mg/mL; (b, e) DMSO/H_2O 1:1 at 10.0 mg/mL; (c, f) DMSO/H_2O 1:2 at 5.0 mg/mL

Figure S5-3 Optical micrographs of selected gels before and after extrusion

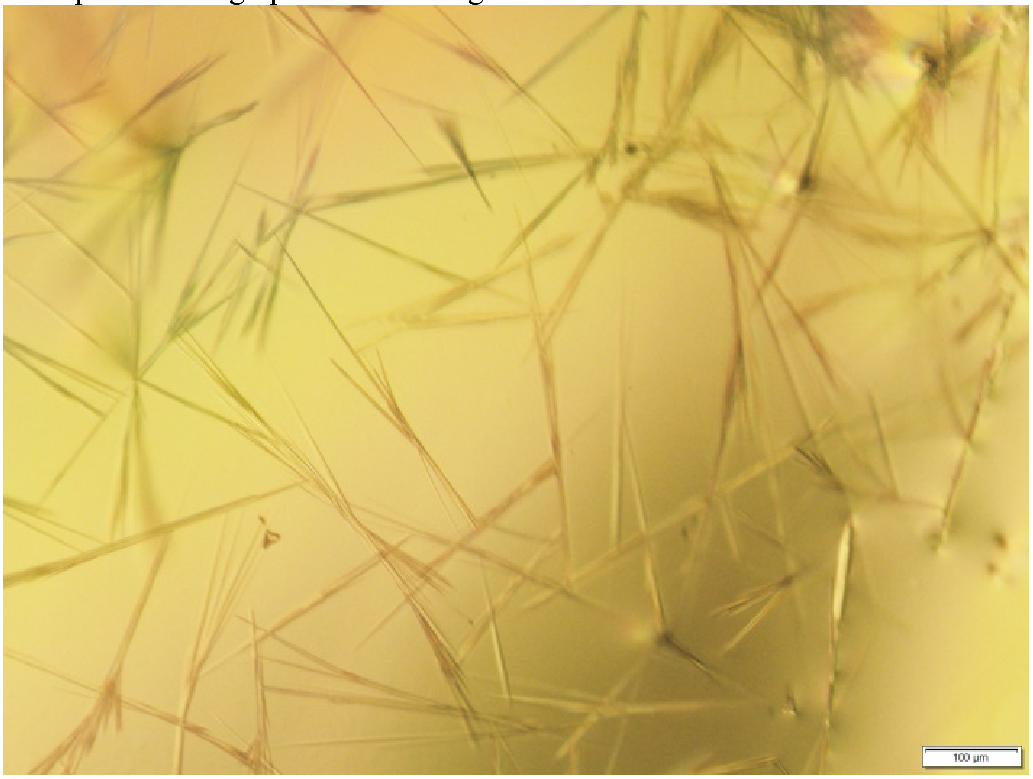

a) The OM image for the wet gel of compound **11** in DMSO/H_2O (1:1) at 5.0 mg/mL

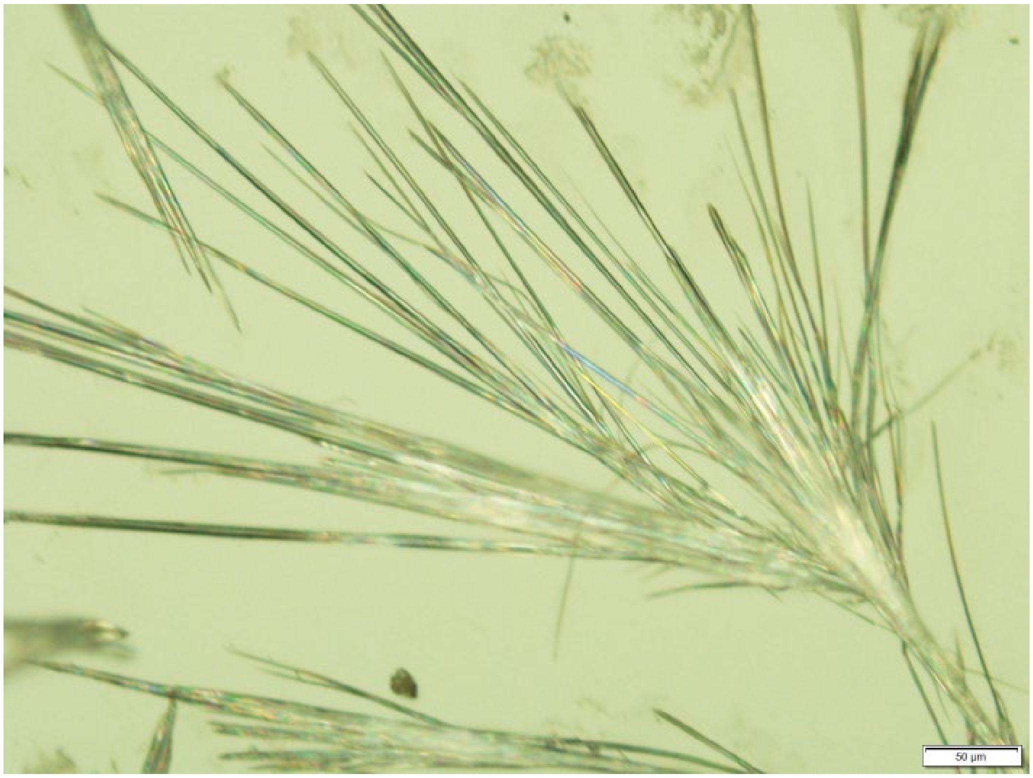

b) The OM image for the dried gel of compound **11** in DMSO/H_2O (1:1) at 5.0 mg/mL

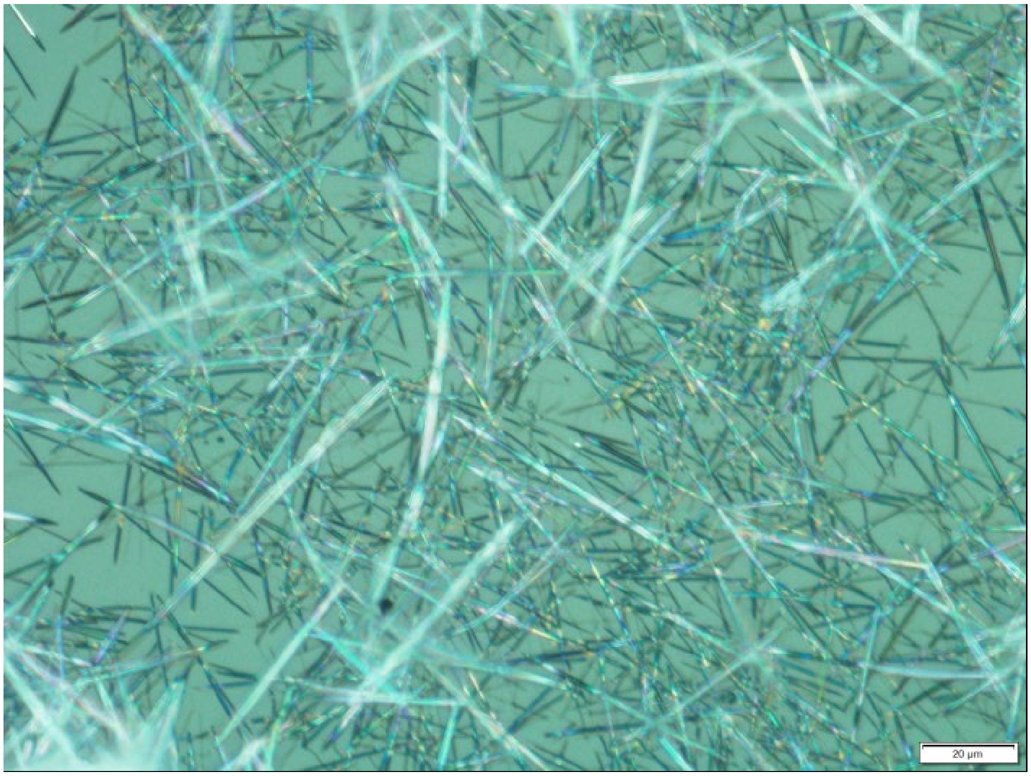

c) The OM image for gel of compound **11** in DMSO/H_2O (1:1) at 5.0 mg/mL, after extrusion

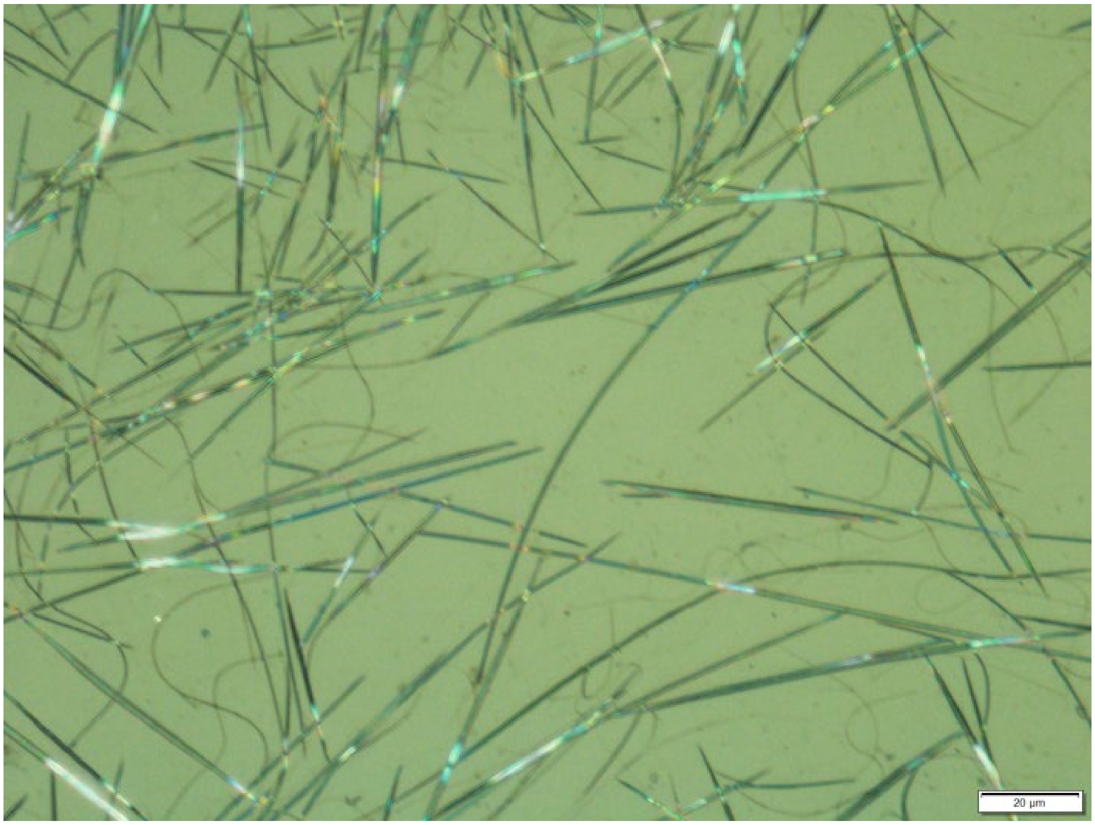

d) The OM image for gel of compound **11** in DMSO/H_2O (1:1) at 5.0 mg/mL, after extrusion

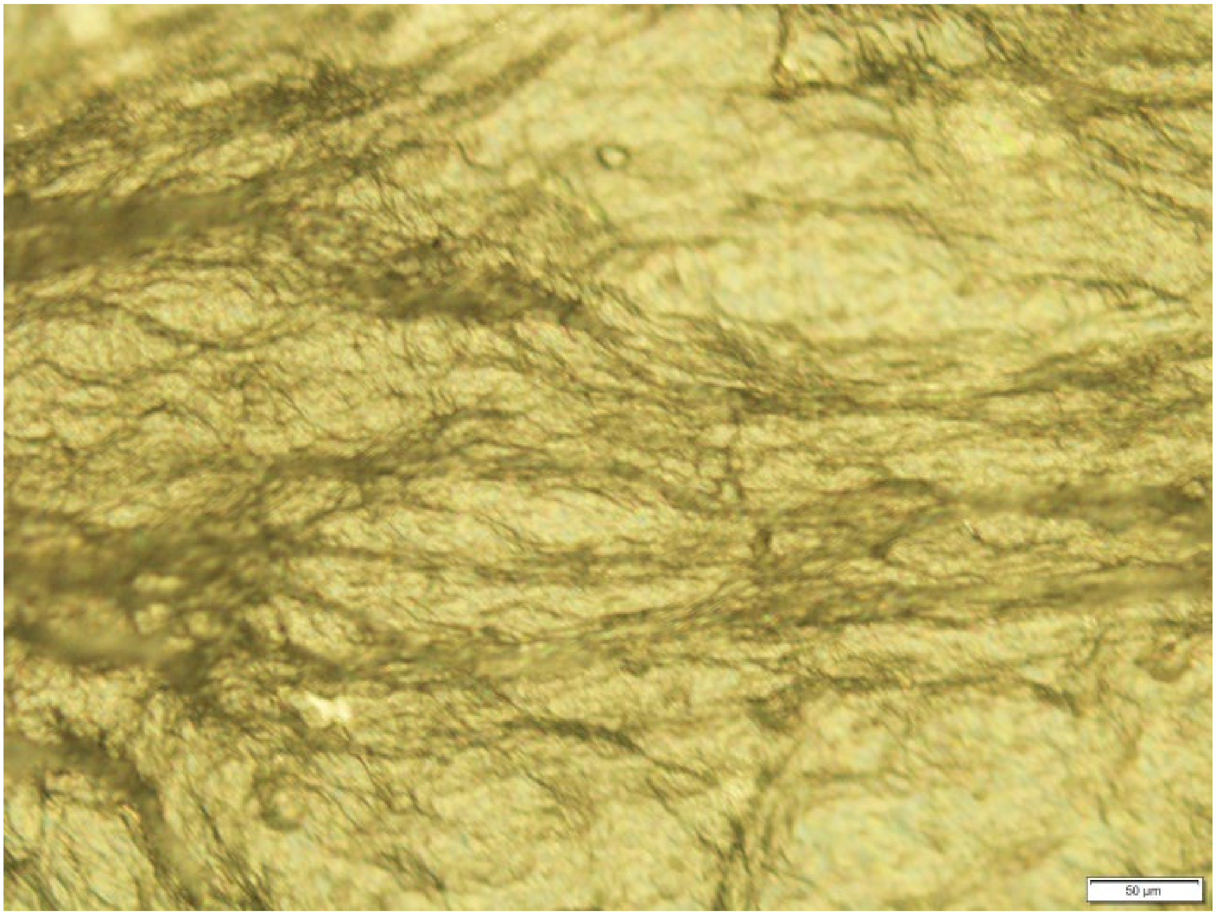

e) The OM image of the gel of compound **13** in DMSO/H_2O (1:1) at 5.0 mg/mL, after extrusion

Figure S5-3 Optical micrographs of selected gels before and after extrusion.

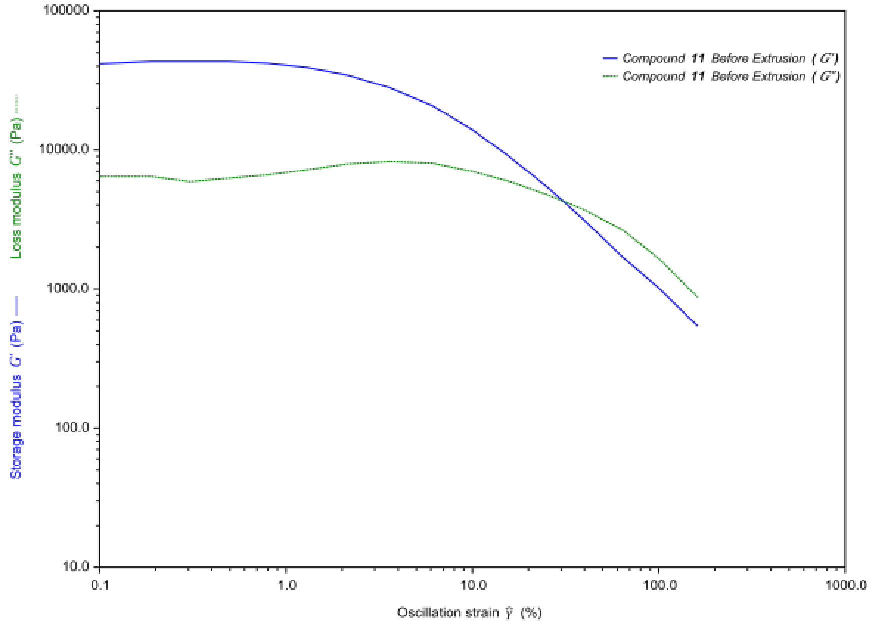

Figure 6a. Amplitude sweeps for the gel of compound **11** in DMSO/H_2O (1:1) at 5.0 mg/mL before extrusion. The frequency sweep rheological graphs are shown in Figure 6.

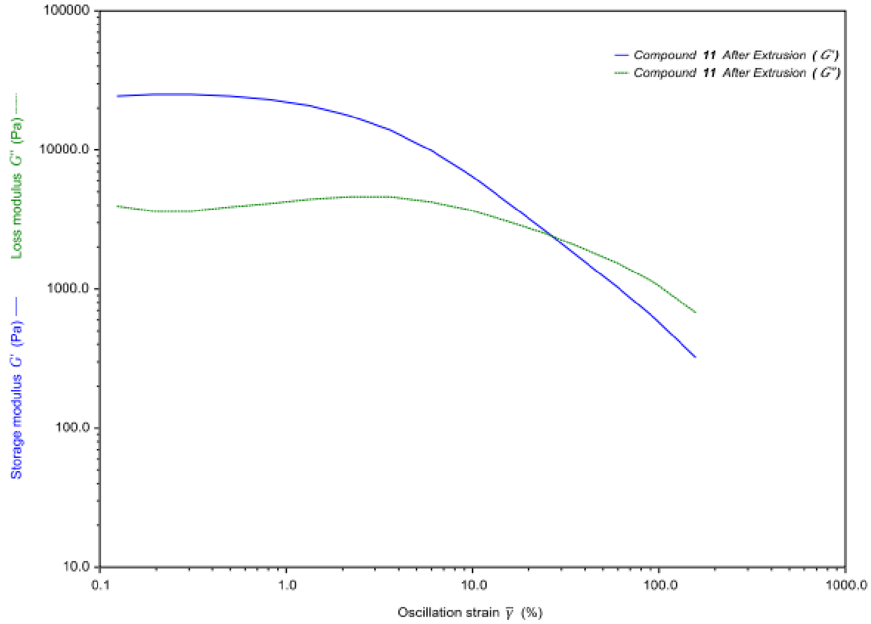

Figure 6b. Amplitude sweeps for the gel of compound **11** in DMSO/H_2O (1:1) at 5.0 mg/mL after extrusion. The frequency sweep rheological graphs are shown in Figure 6.

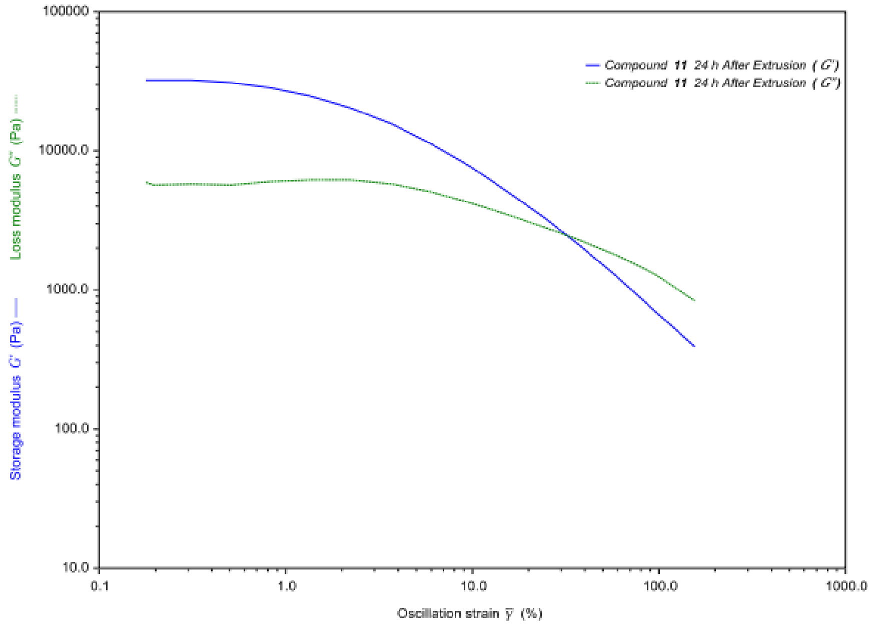

Figure 6c. Amplitude sweeps for the gel of compound **11** in DMSO/H_2O (1:1) at 5.0 mg/mL 24 hours after extrusion. The frequency sweep rheological graphs are shown in Figure 6.

Compound 11 DMSO/H_2 1:1	Before Extrusion			After Extrusion			24 h After Extrusion		
Angular Frequency	Storage Modulus (G')	Loss Modulus (G'')	G'/G''	Storage Modulus (G')	Loss Modulus (G'')	G'/G''	Storage Modulus (G')	Loss Modulus (G'')	G'/G''
0.10	19536.7	3227.8	6.1	5925.5	1026.6	5.8	9231.5	2069.2	4.5
0.16	20138.8	2954.0	6.8	6414.5	960.0	6.7	10209.4	1898.5	5.4
0.25	21126.0	2555.9	8.3	6656.7	910.2	7.3	10838.1	1847.3	5.9
0.40	21855.8	2368.5	9.2	6866.3	897.3	7.7	11419.2	1705.3	6.7
0.63	22401.1	2484.5	9.0	7128.2	818.3	8.7	11842.4	1645.7	7.2
1.00	23017.7	2102.3	11.0	7323.4	767.8	9.5	12200.0	1613.7	7.6
1.58	23491.6	1986.4	11.8	7557.5	748.9	10.1	12668.9	1564.2	8.1
2.51	24064.3	1955.7	12.3	7719.8	744.2	10.4	12988.6	1466.9	8.9
3.98	24495.9	1806.9	13.6	7913.1	728.9	10.9	13378.1	1463.4	9.1
6.31	24996.4	1890.1	13.2	8107.0	723.2	11.2	13759.2	1460.9	9.4
10.00	25434.6	1841.0	13.8	8285.3	720.4	11.5	14093.3	1483.3	9.5
15.85	25909.2	1746.9	14.8	8476.5	736.8	11.5	14457.6	1510.6	9.6
25.12	26345.2	1807.4	14.6	8649.3	775.2	11.2	14887.6	1601.5	9.3
39.81	26826.2	1902.9	14.1	8832.7	825.5	10.7	15289.6	1697.4	9.0
63.10	27295.9	2021.6	13.5	9095.4	925.5	9.8	15798.9	1841.8	8.6
100.00	27969.5	2334.6	12.0	9374.6	1022.0	9.2	16309.7	1965.3	8.3

Table S2. Table of rheometric data for the extrusion study carried out on the gel of compound **11** in DMSO/H_2O 1:1 at 5.0 mg/mL

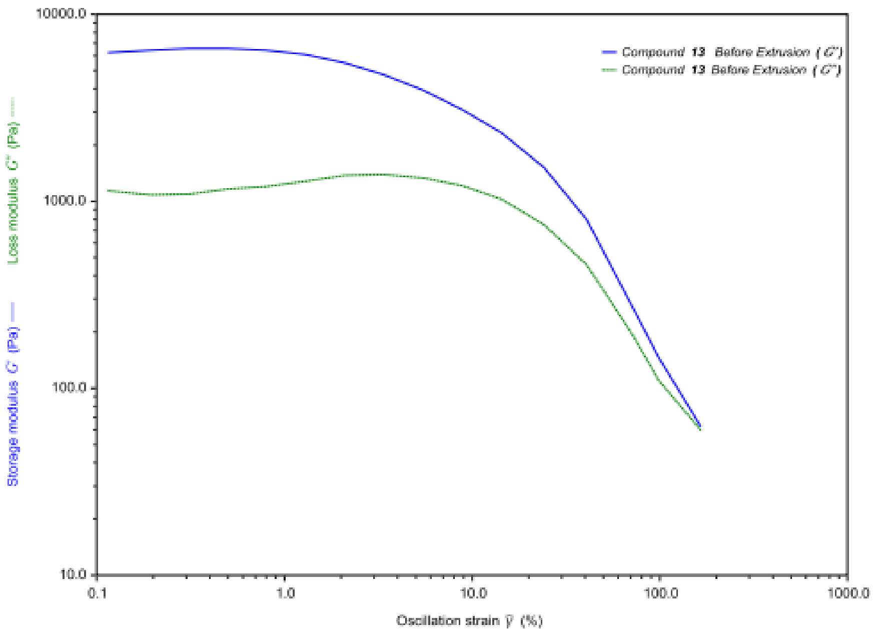

Figure S7a. Amplitude sweeps for the gel of compound **13** at 5 mg/mL in DMSO/H_2O (1:1) before extrusion. The frequency sweep rheological graphs are shown in Figure 5.

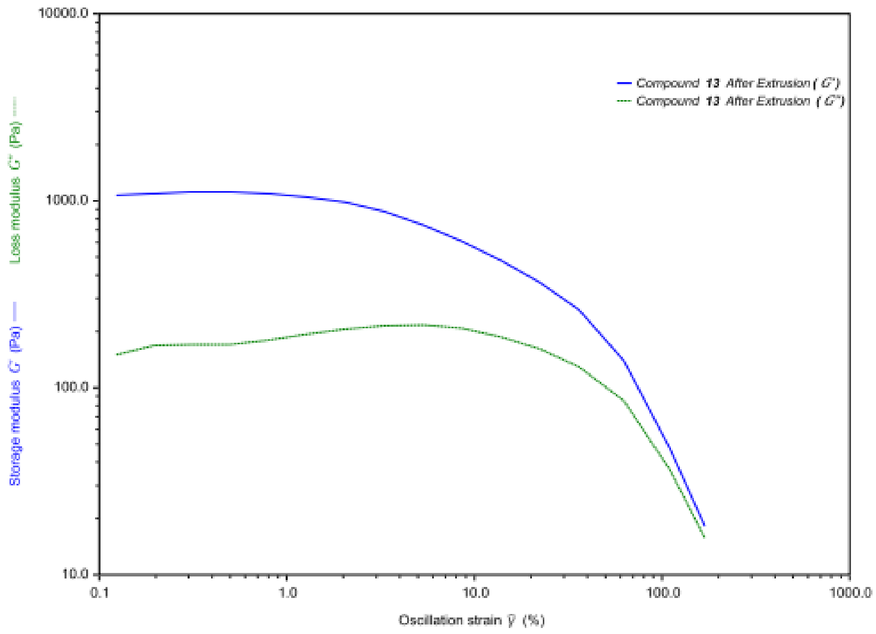

Figure S7b. Amplitude sweeps for the gel of compound **13** at 5 mg/mL in DMSO/H_2O (1:1) after extrusion. The frequency sweep rheological graphs are shown in Figure 5.

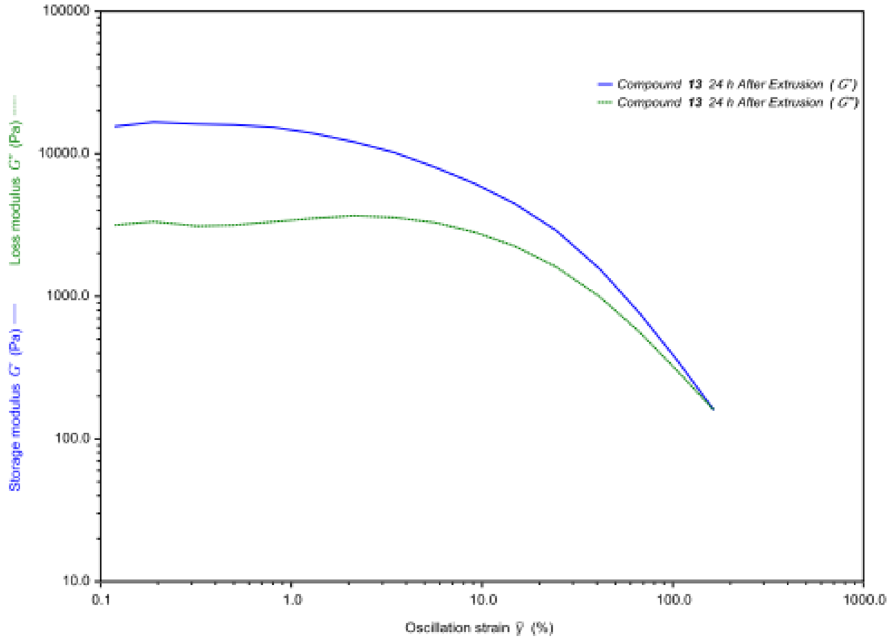

Figure S7c. Amplitude sweeps for the gel of compound **13** at 5.0 mg/mL in DMSO/H_2O (1:1) 24 hours after extrusion. The frequency sweep rheological graphs are shown in Figure 5.

Compound 13 DMSO/H_2 1:1	Before Extrusion			After Extrusion			24 h After Extrusion		
Angular Frequency	Storage Modulus (G')	Loss Modulus (G'')	G'/G''	Storage Modulus (G')	Loss Modulus (G'')	G'/G''	Storage Modulus (G')	Loss Modulus (G'')	G'/G''
0.10	15783.1	4314.9	3.7	7956.6	1820.3	4.4	16511.9	4702.9	3.5
0.16	18128.2	4130.8	4.4	8771.8	1753.3	5.0	20164.9	5057.6	4.0
0.25	19581.4	4025.2	4.9	9273.9	1679.2	5.5	21604.1	4700.7	4.6
0.40	20692.8	3783.2	5.5	9771.1	1608.7	6.1	22993.2	4562.3	5.0
0.63	21589.8	3770.7	5.7	10236.1	1546.7	6.6	23873.2	4407.6	5.4
1.00	22513.5	3727.8	6.0	10615.4	1589.8	6.7	24877.3	4168.2	6.0
1.58	23458.6	3752.9	6.3	11014.7	1560.6	7.1	25907.9	4125.7	6.3
2.51	24377.9	3744.2	6.5	11362.9	1539.2	7.4	26696.2	4051.1	6.6
3.98	25216.9	3620.6	7.0	11758.1	1569.9	7.5	27757.4	4027.7	6.9
6.31	26110.5	3637.2	7.2	12160.7	1518.5	8.0	28651.3	4002.0	7.2
10.00	26880.5	3732.9	7.2	12478.5	1526.7	8.2	29594.1	4003.8	7.4
15.85	27819.9	3825.7	7.3	12851.4	1552.3	8.3	30548.8	4073.8	7.5
25.12	28803.7	3870.4	7.4	13256.0	1670.5	7.9	31538.0	4186.8	7.5
39.81	29792.7	3917.0	7.6	13699.7	1671.6	8.2	32682.9	4319.9	7.6
63.10	30895.0	4272.1	7.2	14185.8	1755.2	8.1	33983.7	4441.1	7.7
100.00	31772.2	4029.0	7.9	14704.8	1783.2	8.2	34992.7	4607.4	7.6

Table S3. Table of rheometric data for the extrusion study carried out on the gel of compound **13** at 5.0 mg/mL in DMSO/H_2O 1:1.

VI. 1. Chloramphenicol trapping and release from the gel by compound **13**

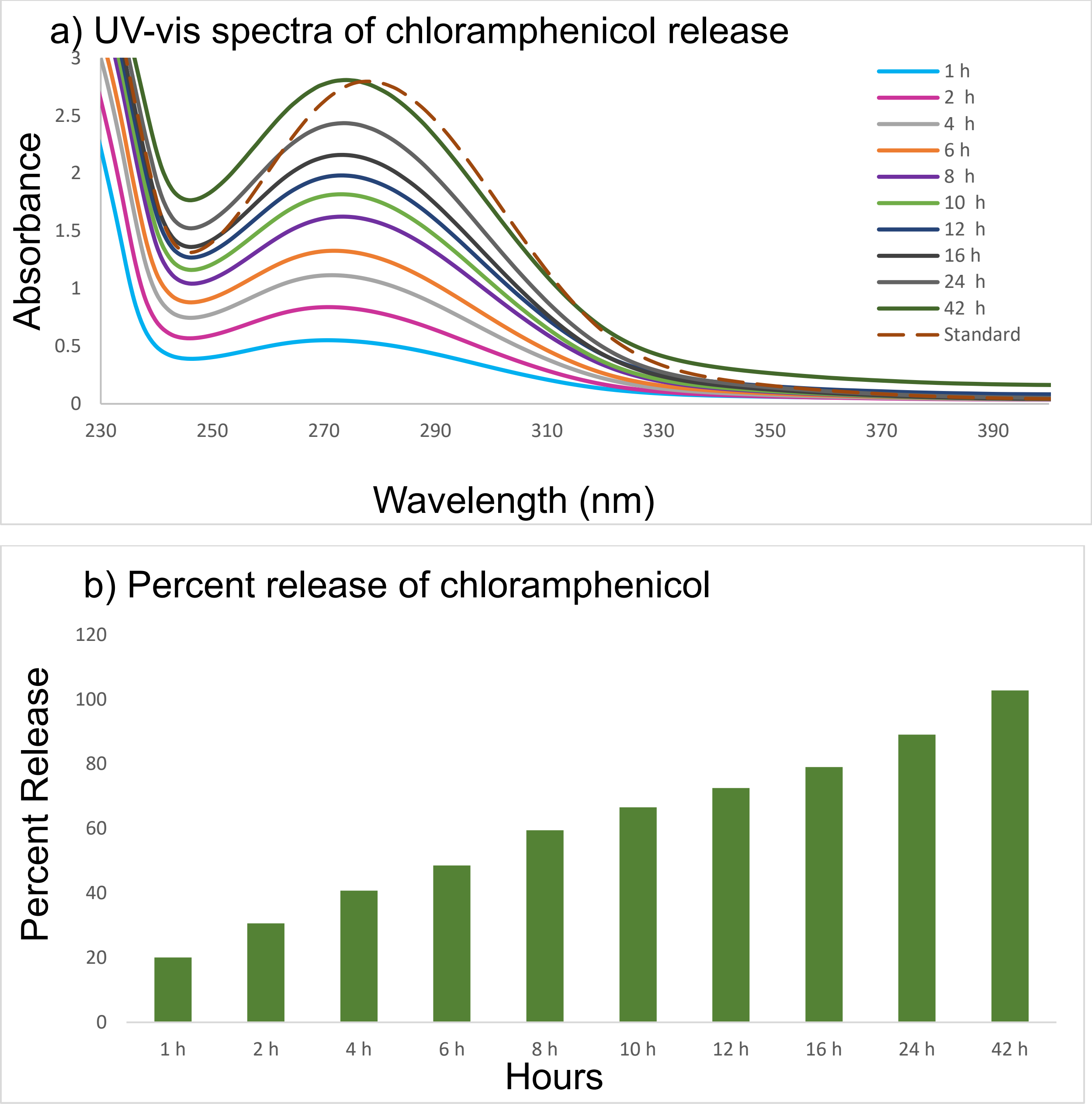

Figure S8A. The UV-Vis spectra (a) and percent release (b) of chloramphenicol over time from a co-gel formed by chloramphenicol and compound **13** (1 mg/mL) in DMSO:H_2O (v/v 5:95). The gel was prepared using 2 mg of compound **13** and 0.4 mg of chloramphenicol in 2.0 mL of DMSO:H_2O (v/v 5:95) solution. Then 2.0 mL of pure water (pH 7) was placed on top of the gel. Percent release was calculated using the absorption values at 273 nm for each time point.

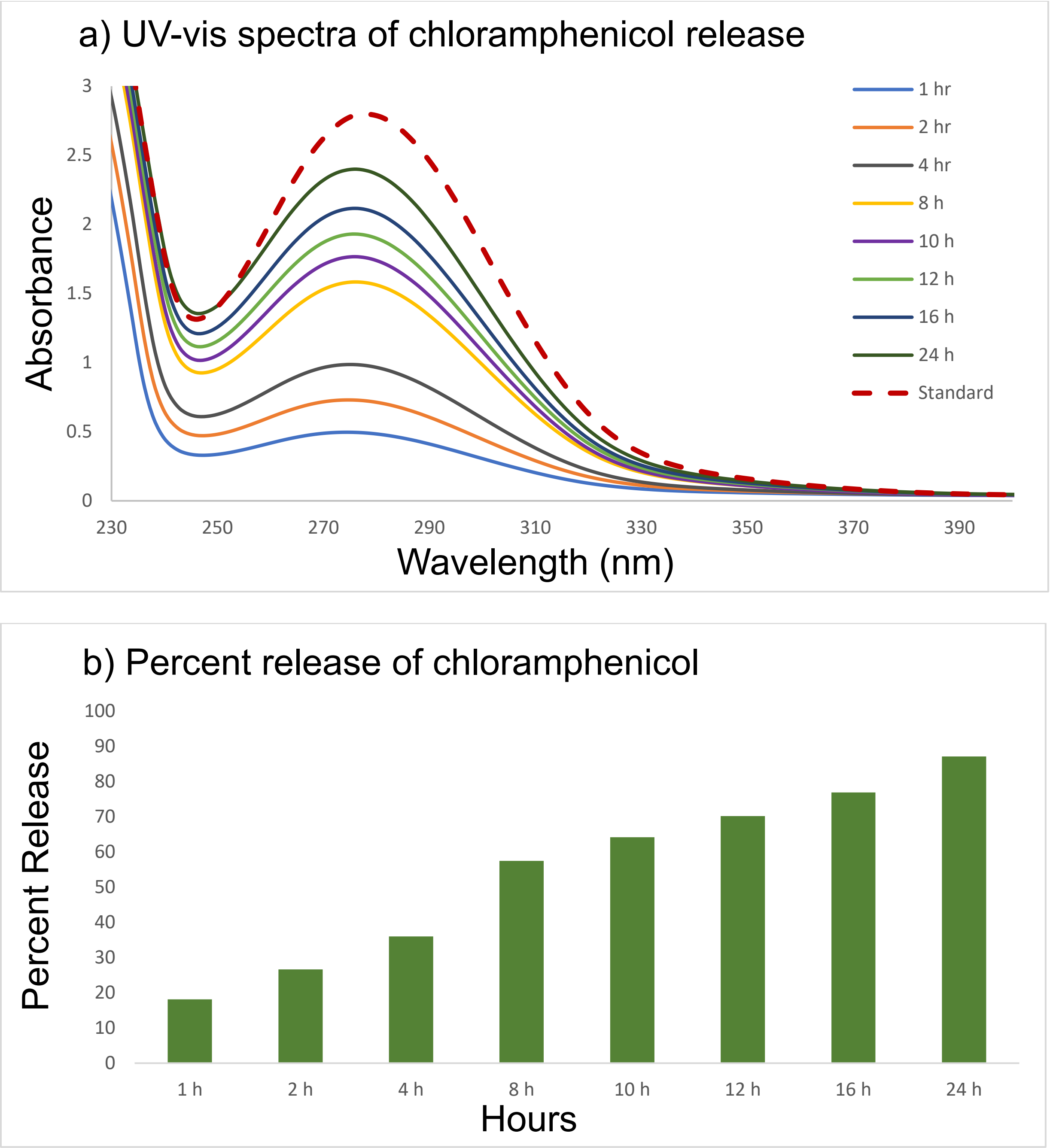

Figure S8B. The UV-Vis spectra and release profile of chloramphenicol at different time course for the gel formed by compound **13** in DMSO:H_2O (v/v 5:95). The gel was prepared using 1 mg of compound **13** and 0.4 mg of chloramphenicol in 2.0 mL of DMSO:H_2O (v/v 5:95) solution. Then 2.0 mL of pure water (pH 7) was placed on top of the gel. Percent release was calculated using the absorption values at 273 nm for each time point.

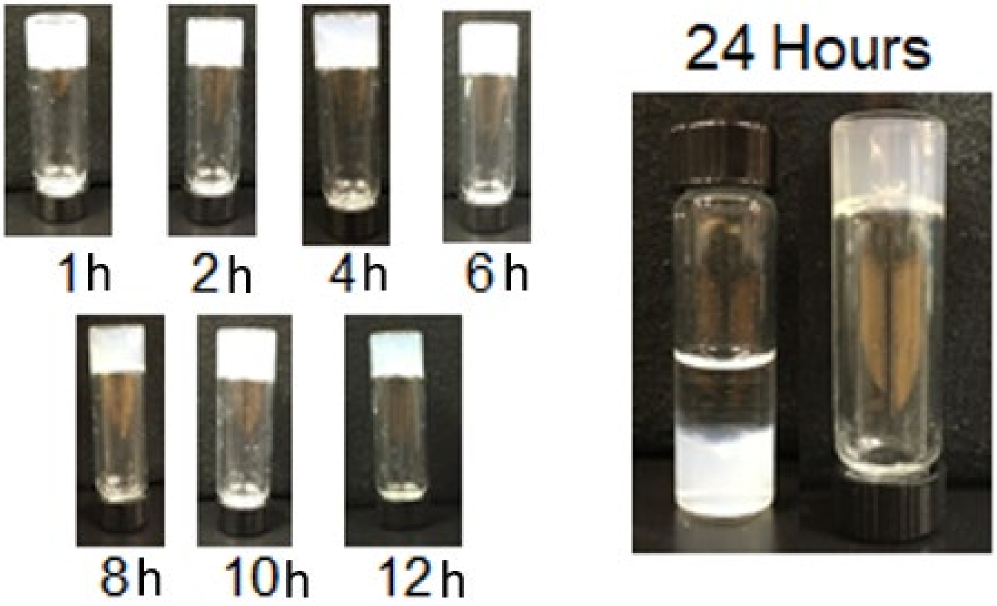

Figure S9a. Gel photos at different times from the chloramphenicol release study

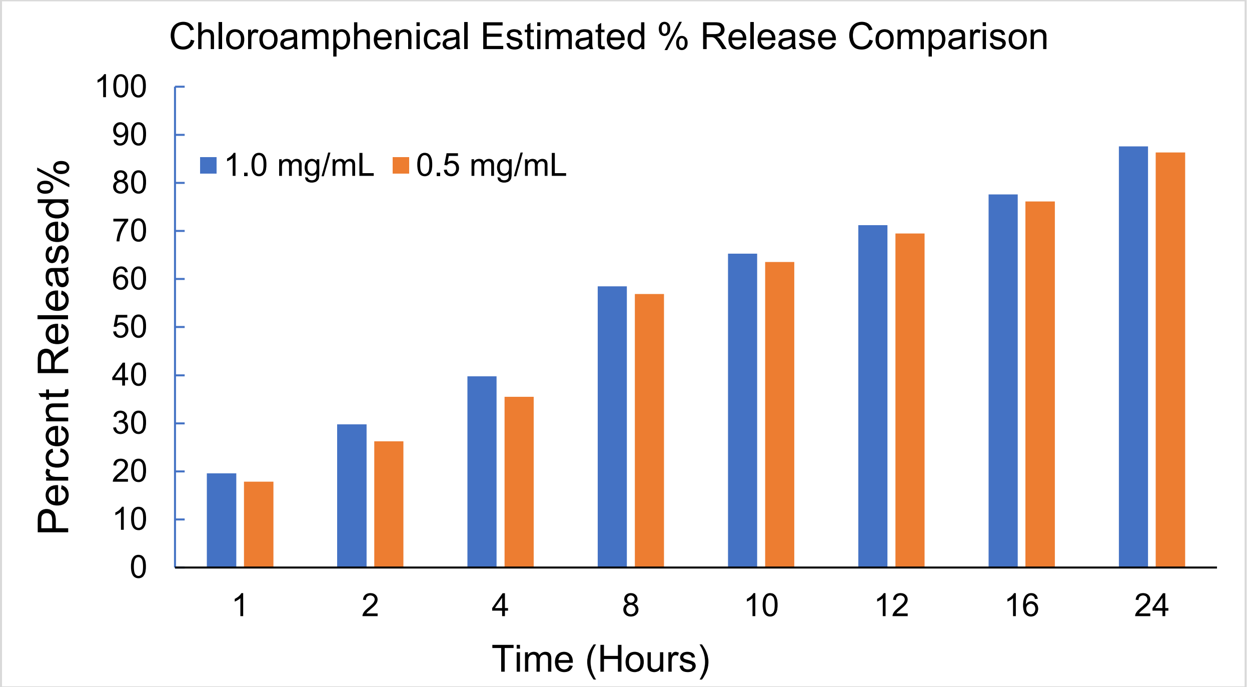

Figure S9b. Comparison of the estimated % release of chloramphenicol from the two diffusion studies carried out using a 0.5 mg/mL gel and a 1.0 mg/mL gel of compound 13 in DMSO:H_2O (v/v 5:95).

2. Additional naproxen trapping and release study using a gel formed by compound **13**

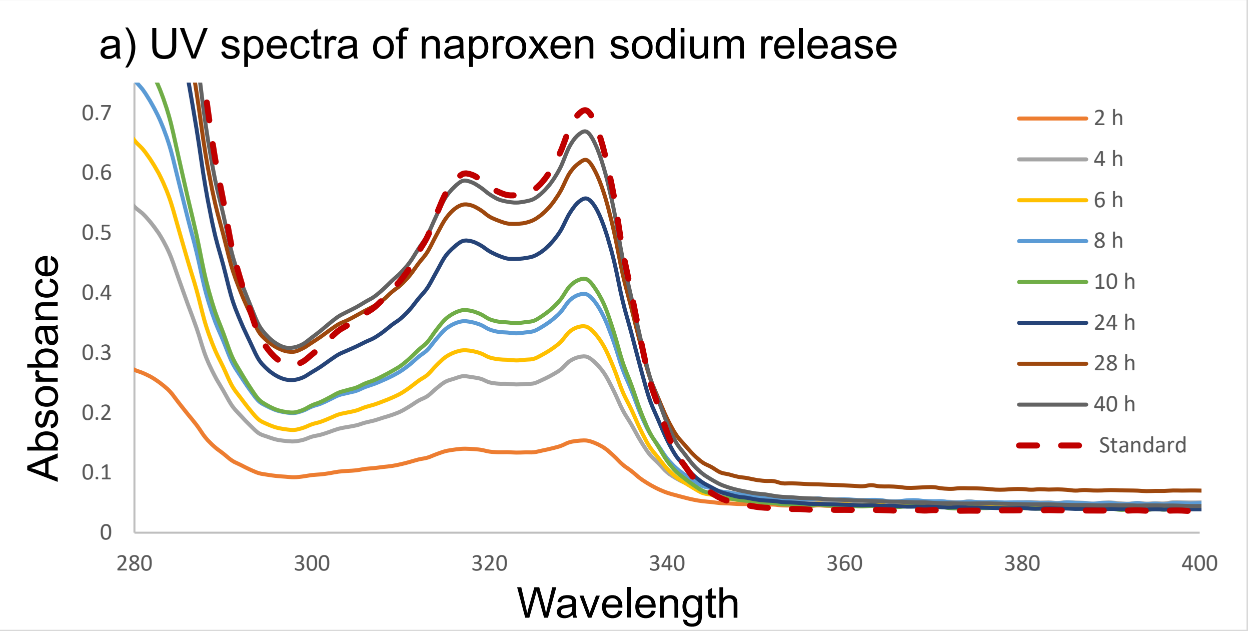

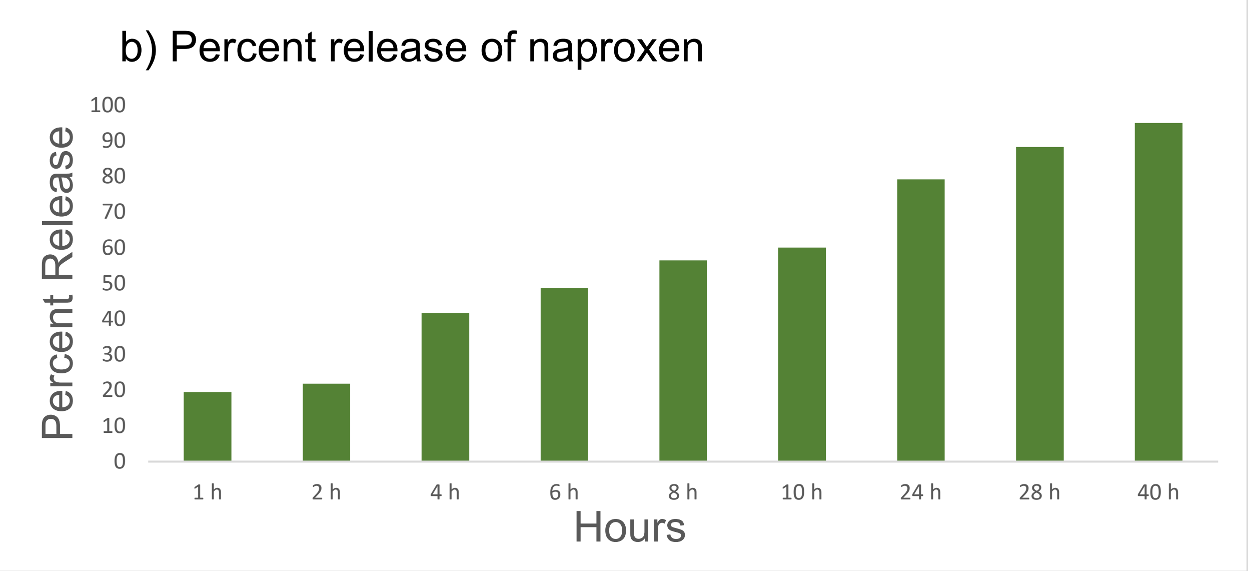

Figure S10. The UV-Vis spectra and release profile of naproxen sodium at different time course for the gel formed by compound **13** in DMSO:H_2O (v/v 5:95). The gel was prepared using 1 mg of compound **13** and 0.5 mg of naproxen sodium in 2.0 mL of DMSO:H_2O (v/v 5:95) solution. Then 2.0 mL of pure water (pH 7) was placed on top of the gel.

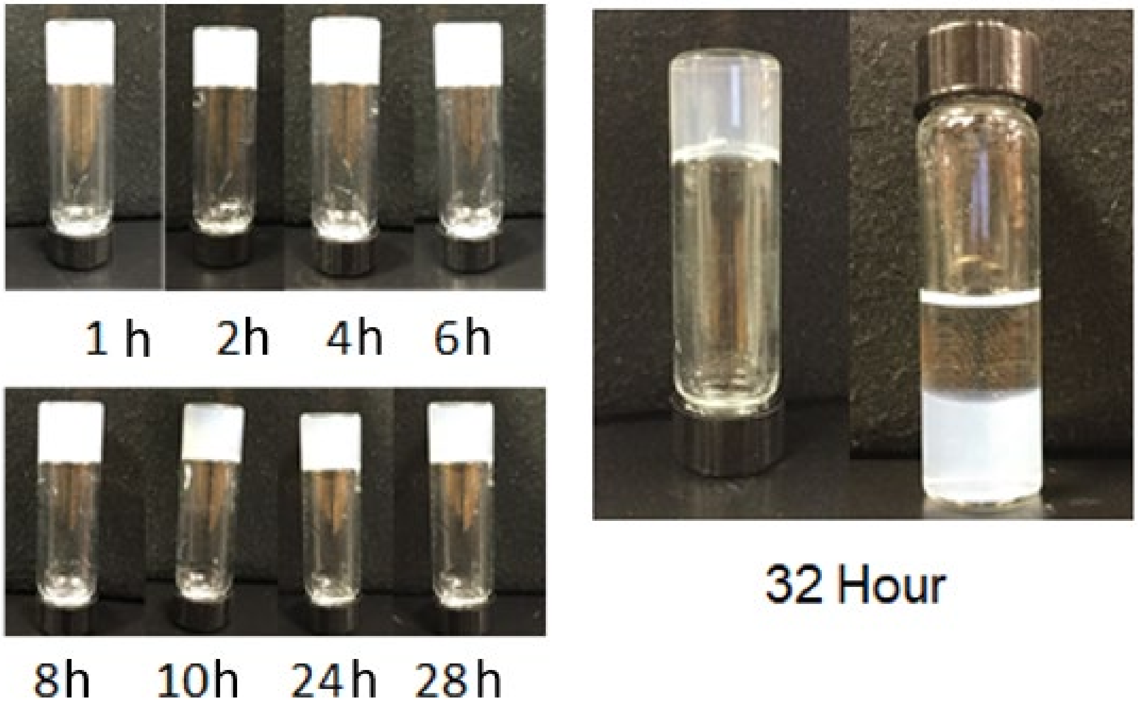

Figure S11a. Gel photos of the naproxen encapsulation and release at different time intervals.

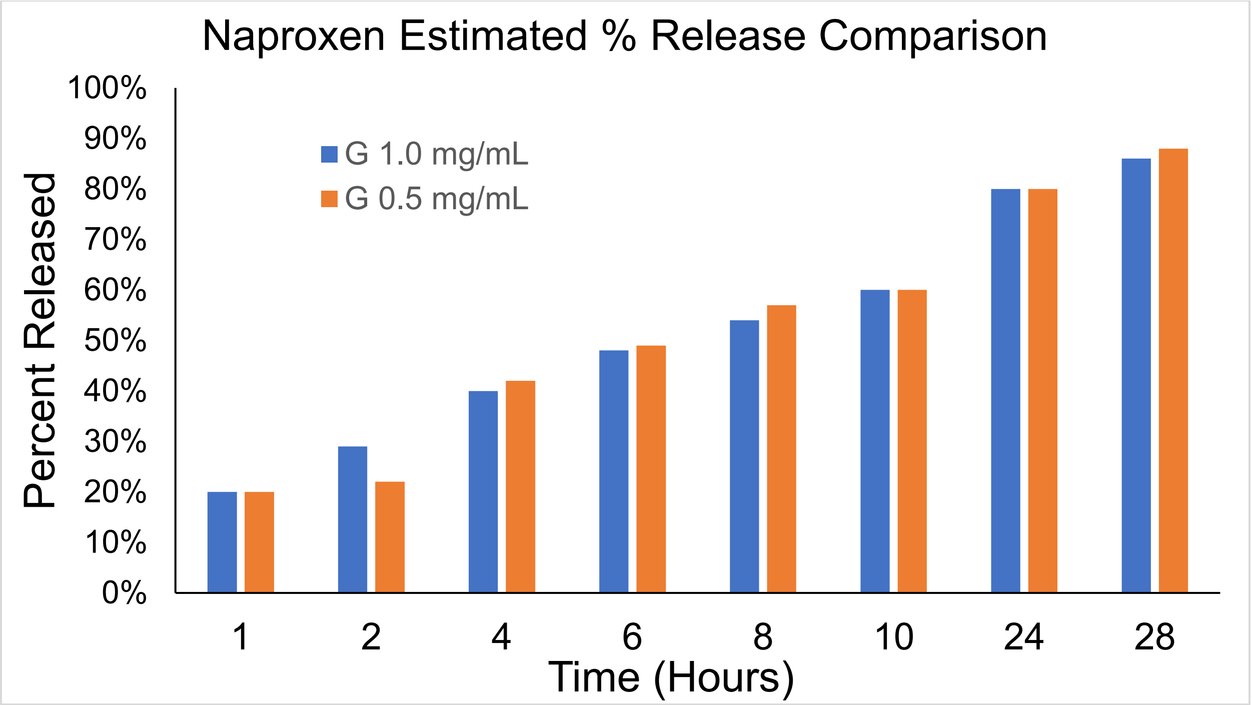

Figure S11b. Comparison of the estimated % release of naproxen from the two diffusion studies carried out using a 0.5 mg/mL gel and a 1.0 mg/mL gel of compound 13 in DMSO:H_2O (v/v 5:95).

The UV-Vis spectra of compound **13** in DMSO and 5% DMSO/water are shown in Figure S12. These spectra show absorption bands from 250-300 nm, which do not overlap with the absorption bands of naproxen; however, they do overlap with the absorption bands of chloramphenicol. With this, it is still unlikely that the gelator is responsible for the adding to the UV-vis absorption in the release study due to its very low solubility in ambient temperature water. The UV-vis samples at 0.1 mg/mL and 0.2 mg/mL required significant heating and sonication in order to dissolve the gelator in the 5% DMSO/H_2O solvent system. Without heating and sonication, the gelator remained undissolved and the UV-vis spectra was indistinguishable from the blank. Additionally, after 24 hours, the gel matrix did not appear to be disturbed or degraded, shown in Figure S9A. Because of this, the absorption observed in the UV-Vis spectra for the chloramphenicol release studies should be mostly from the drug itself.

3. UV-vis spectra of compound **13**

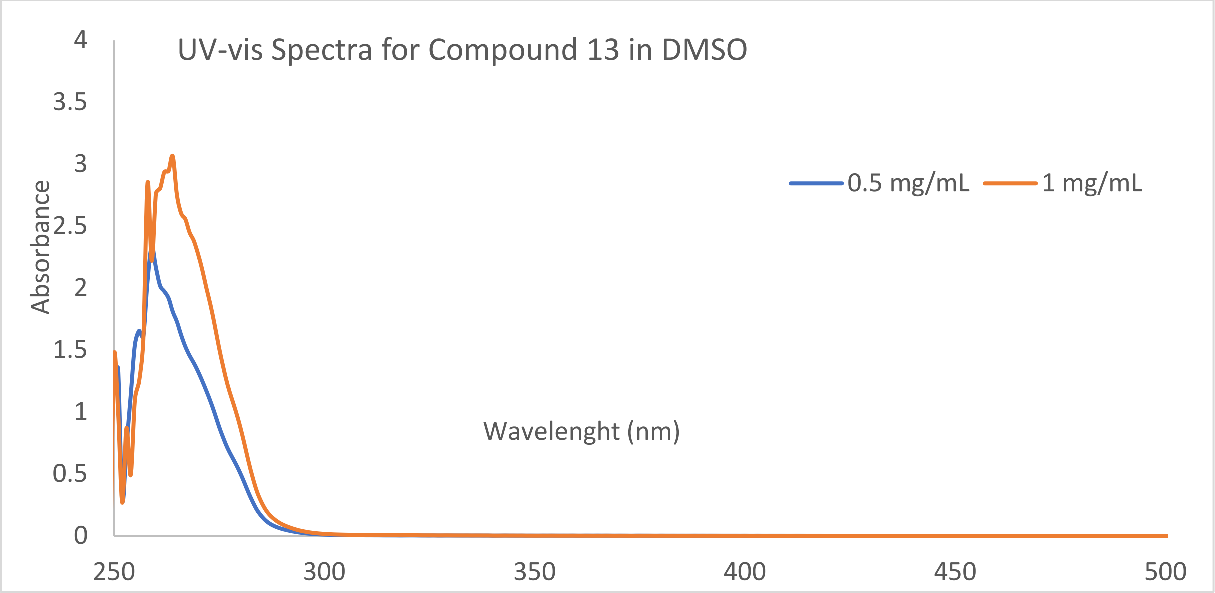

Figure S12a. Overlayed UV-vis spectra of compound **13** in DMSO at 0.5 mg/mL and 1.0 mg/mL.

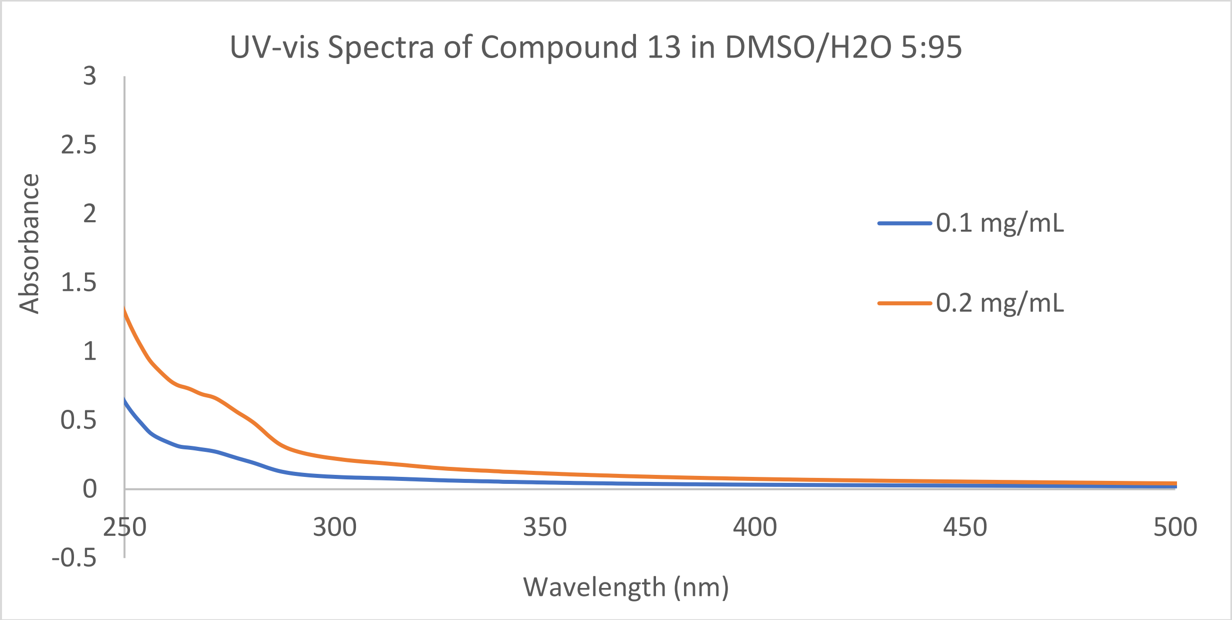

Figure S12b. UV-vis spectra of compound **13** in DMSO/H_2O 5:95 at 0.1 mg/mL and 0.2 mg/mL.

VII. 1. Calibration curve for TBO

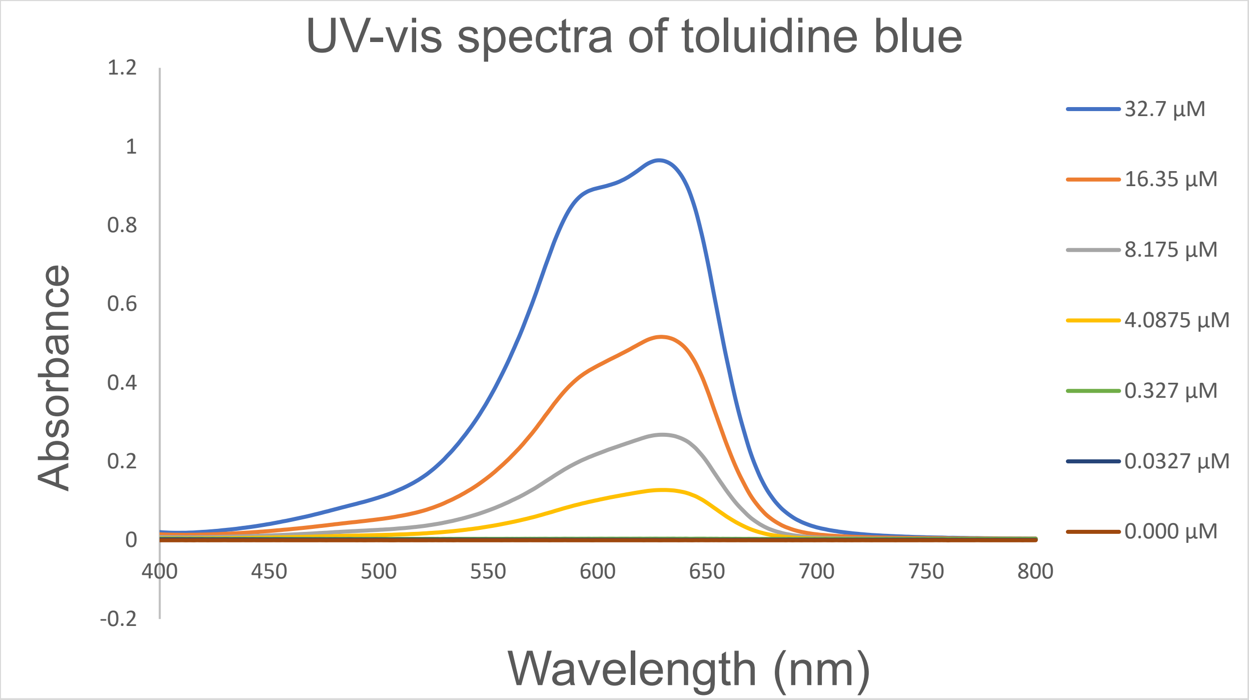

Figure S13. Various UV-vis spectra obtained from a seriel dilution of Toluidine Blue

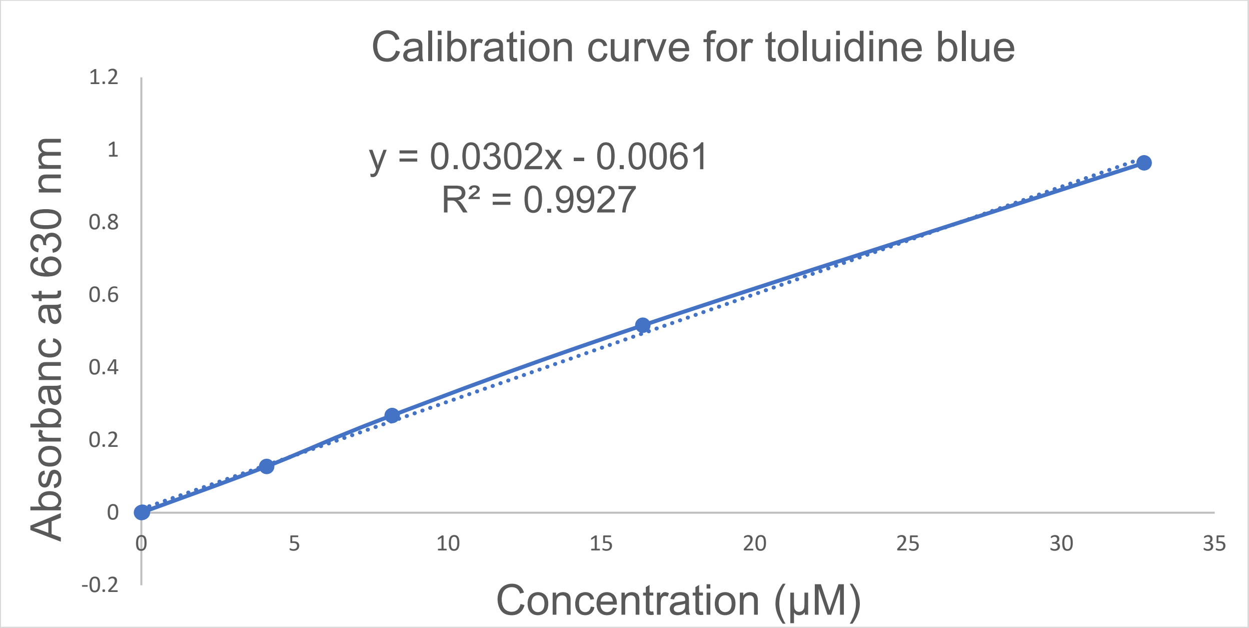

Figure S14. Calibration curve obtained from absorbance reading at 630 nm (λmax)

2. Dye absorption studies using the gels formed by compound **13**

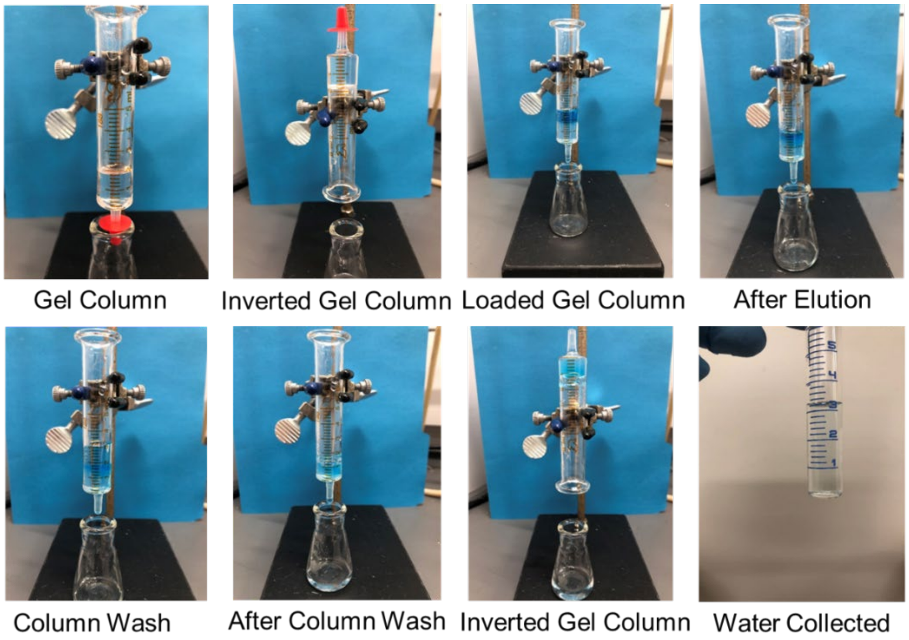

Figure S15. Images taken over the course of the dye absorption study carried out using a 2 mL gel formed by compound **13** in DMSO/water 1:1 at a concentration of 0.5 mg/mL.

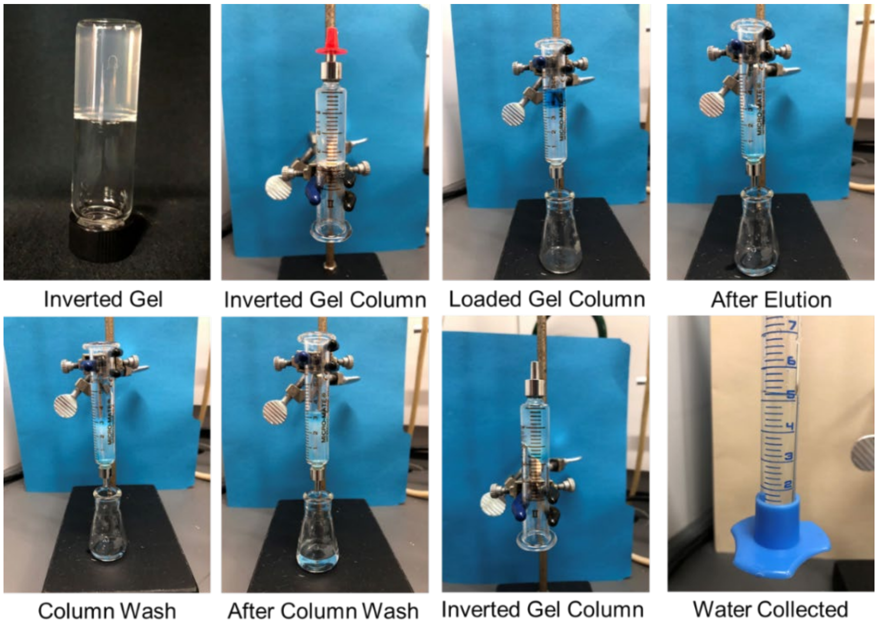

Figure S16. Images taken over the course of the dye absorption study carried out using a 4 mL gel formed by compound **13** in EtOH/water 1:2 at a concnetration of 0.5 mg/mL.

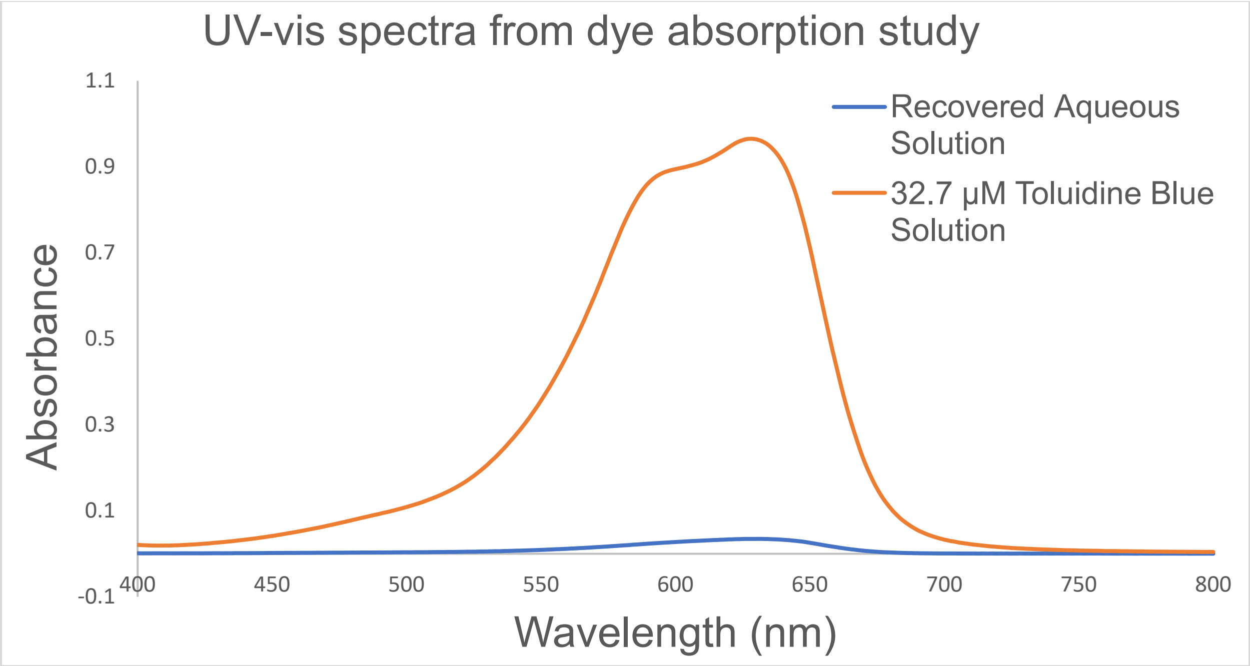

Figure S17. Overlay of UV-vis spectra of recovered aqueous layer from the study carried out using a 4 mL gel formed by compound **13** in EtOH/water 1:2 at a concentration of 0.5 mg/mL and the initial toluidine blue stock solution.

VIII. LCMS spectra for compounds **3-18**

LCMS spectra for compound **3**, m/z calculated for $C_{16}H_{20}ClNO_6$ $[M+H]^+$ 380.1 found 380.1

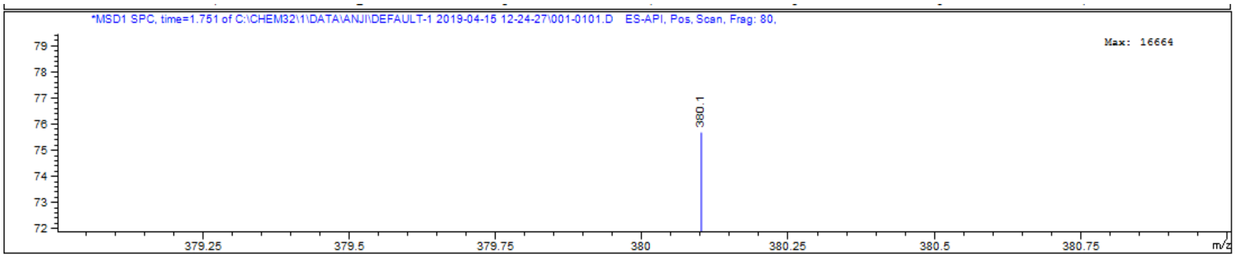

LCMS spectra for compound **4**, m/z calculated for $C_{14}H_{18}ClNO_5$ $[M+H]^+$ 316.1 found 316.1

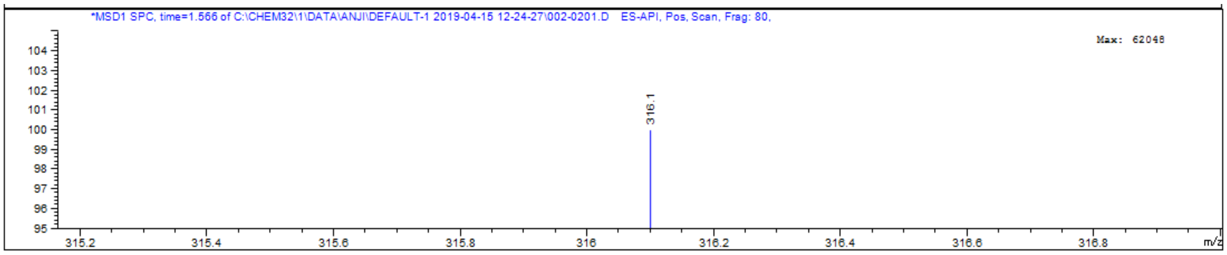

LCMS spectra for compound **5,** m/z calculated for $C_{17}H_{22}ClNO_6$ $[M+H]^+$ 372.1 found 372.2

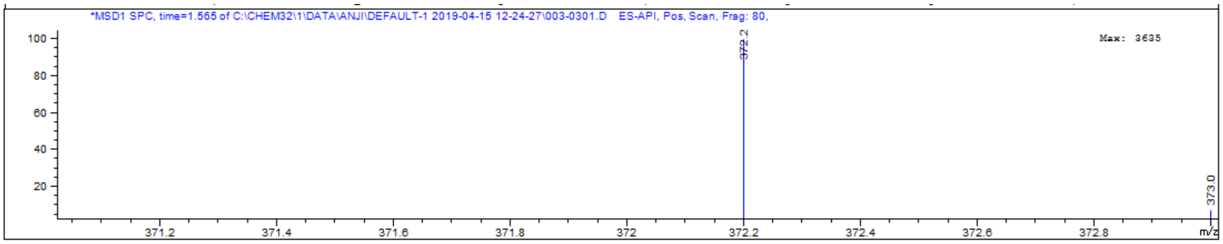

LCMS spectra for compound **6,** m/z calculated for $C_{19}H_{26}ClNO_6$ [M+Na]+ 422.1 found 422.1

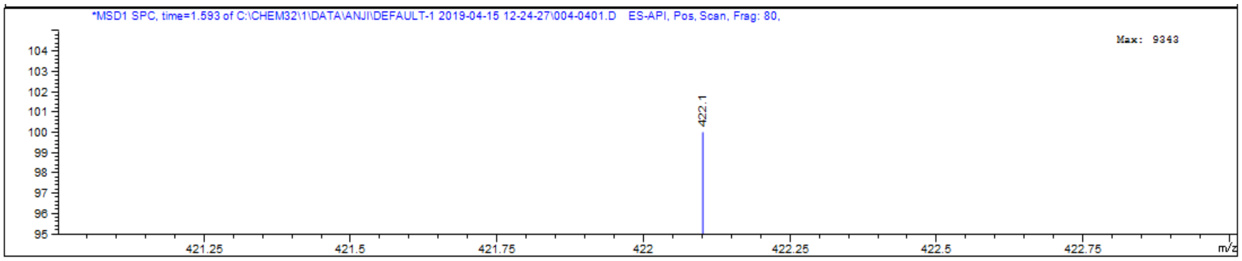

LCMS spectra for compound **7,** m/z calculated for $C_{20}H_{28}ClNO_6$ $[M+H]^+$ 414.2 found 414.3 and $[M+Na]^+$ 436.2 found 436.3

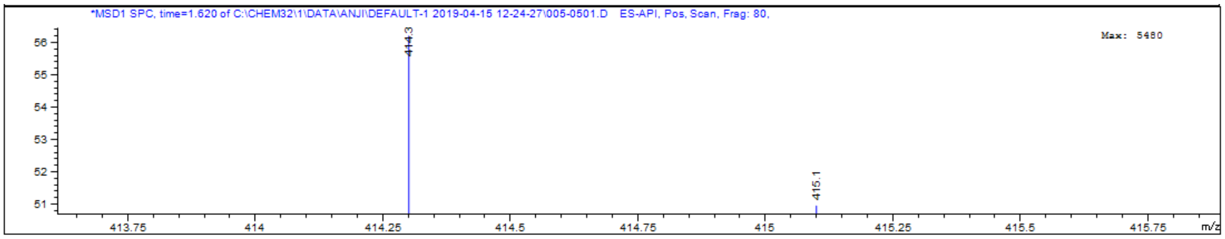

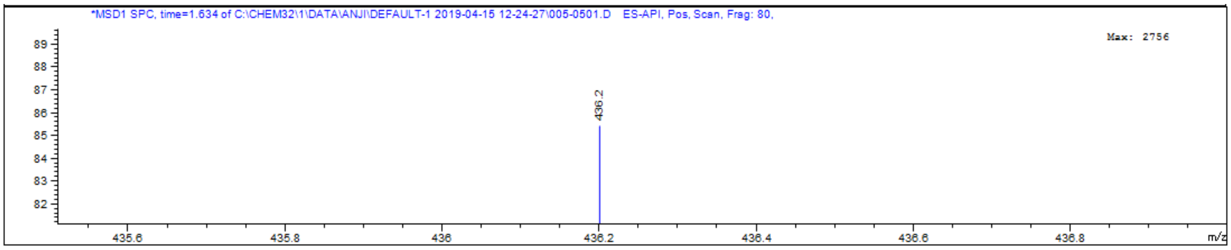

LCMS spectra for compound **8**, m/z calculated for $C_{21}H_{30}ClNO_6$ $[M+H]^+$ 428.2 found 428.2

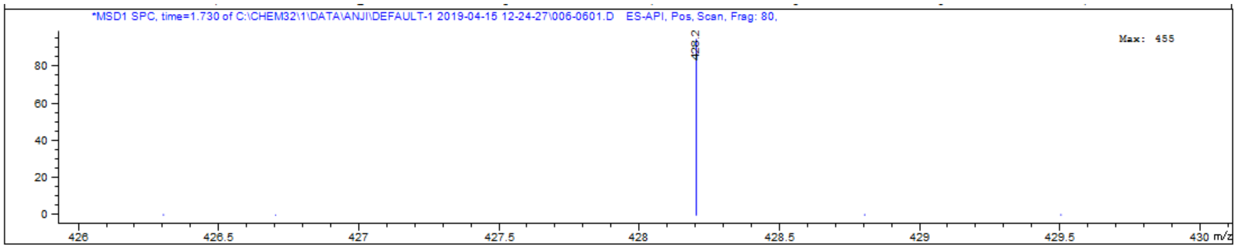

LCMS spectra for compound **9**, m/z calculated for $C_{21}H_{28}ClNO_6$ $[M+H]^+$ 426.1 found 426.1.

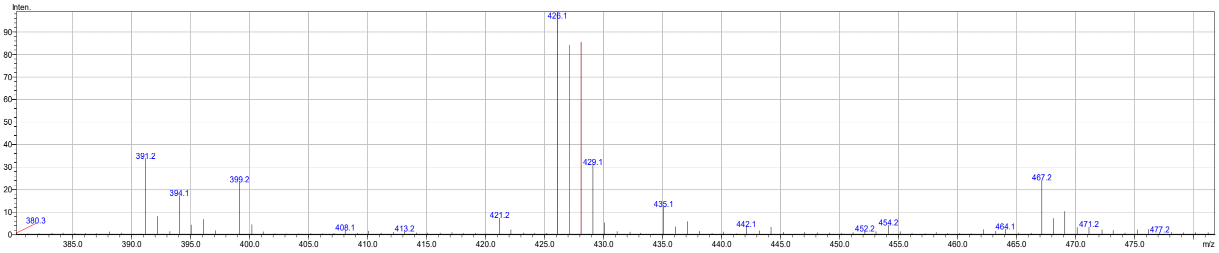

LCMS spectra for compound **10**, m/z calculated for $C_{19}H_{26}ClNO_6$ $[M+H]^+$ 400.1 found 400.1 and $[M+Na]^+$ 422.1 found 422.1

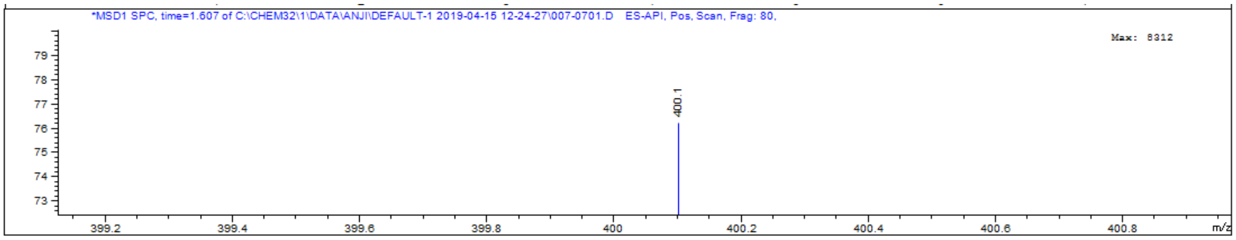

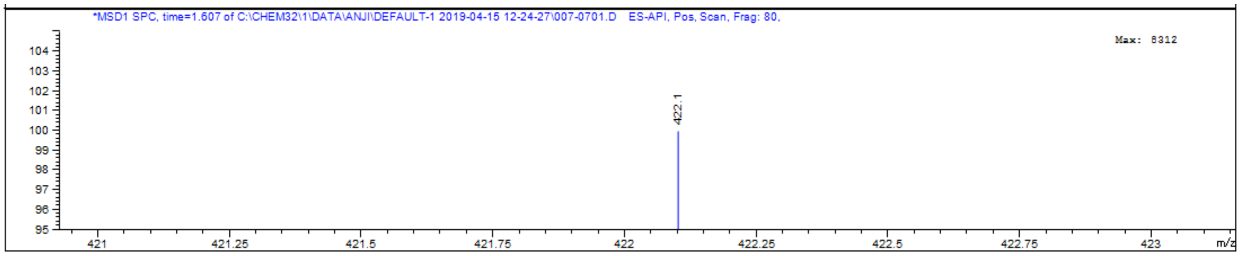

LCMS spectra for compound **11**, m/z calculated for$C_{16}H_{17}ClF_3NO_6$ $[M+Na]^+$ 434.1 found

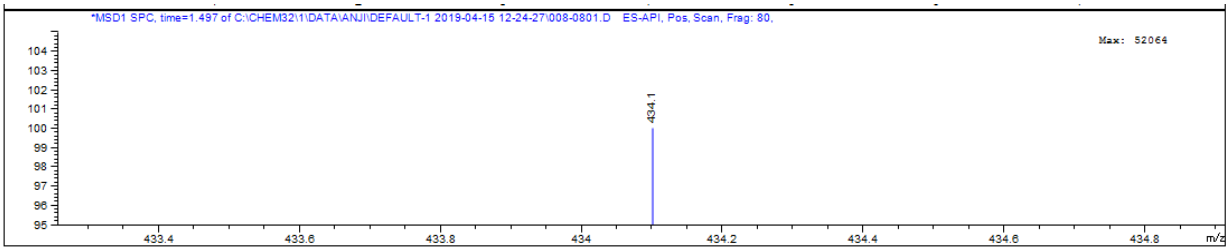

LCMS spectra for compound **12** m/z calculated for $C_{21}H_{21}Cl_3ClNO_6$ $[M+Na]^+$ 482.1 and 482.1

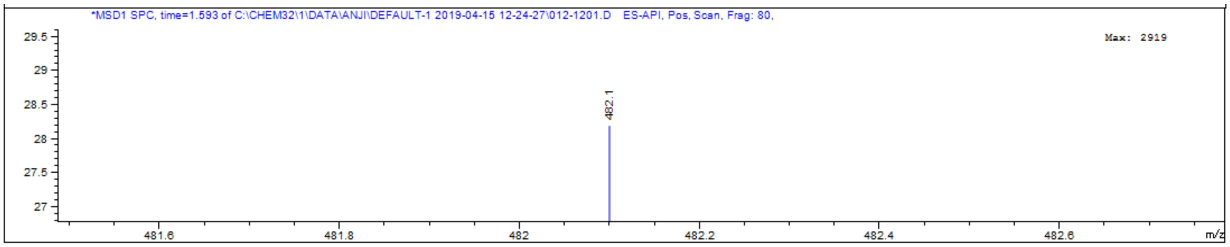

LCMS spectra for compound **13**, m/z calculated for $C_{21}H_{22}ClNO_6$ $[M+H]^+$ 420.1 found 420.2 and $[M+Na]^+$ 442.1 found 442.1

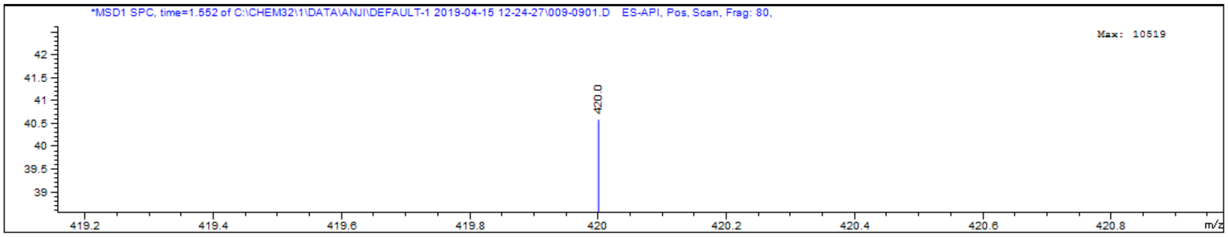

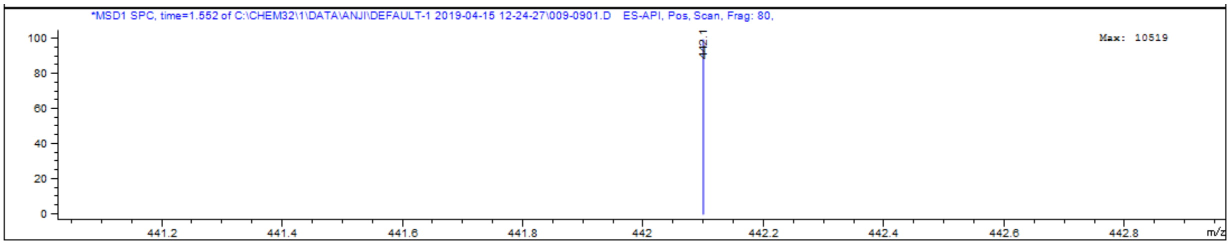

LCMS spectra for compound **14**, m/z calculated for $C_{21}H_{21}BrClNO_6$ $[M+H]^+$ 498.0 and 500.0 found 497.9 and 499.9

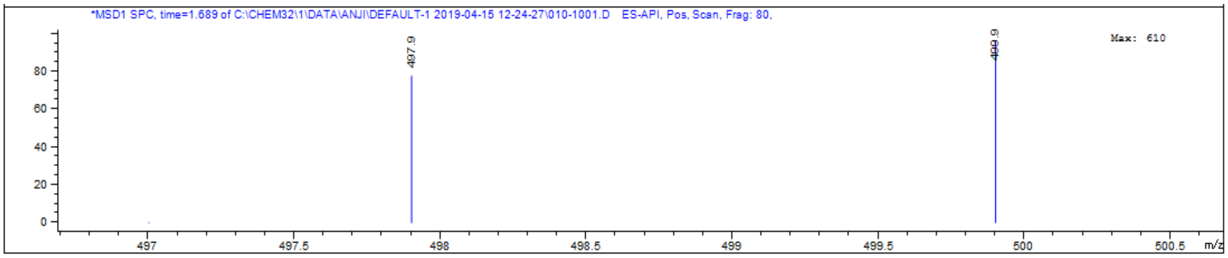

LCMS spectra for compound **15**, m/z calculated for LC-MS m/z calculated for $C_{21}H_{21}Cl_2NO_6$ $[M+H]^+$ 454.1 found 454.0

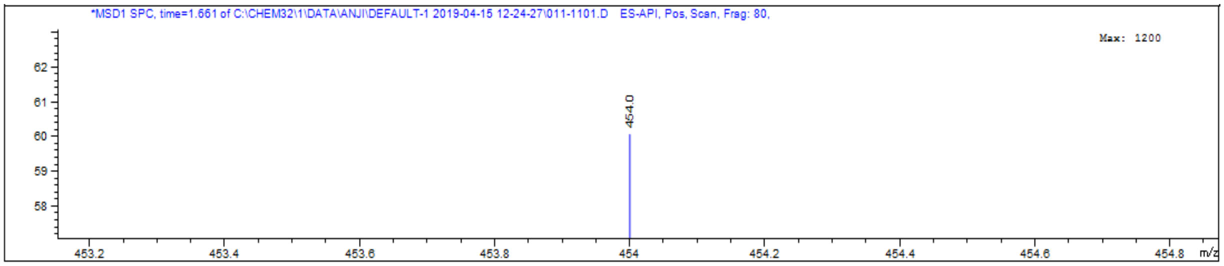

LC-MS spectra for compound **16**, m/z calculated for $C_{25}H_{24}ClNO_6$ 470.1 found 470.

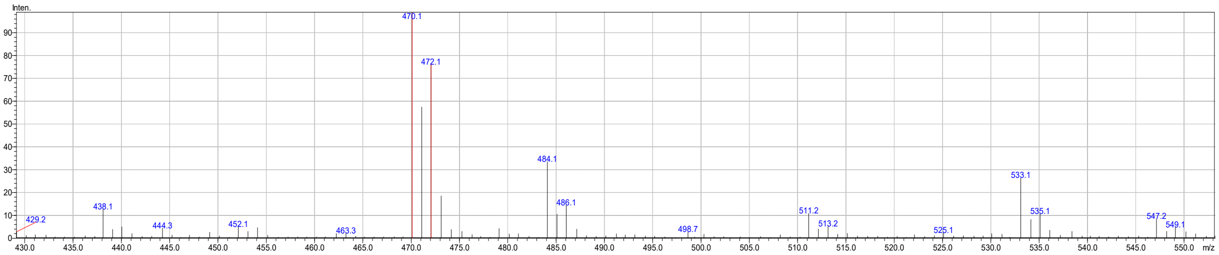

LC-MS spectra for compound **17**, m/z calculated for $C_{26}H_{26}ClNO_6$ $[M+H]^+$ 484.1 found 484.1

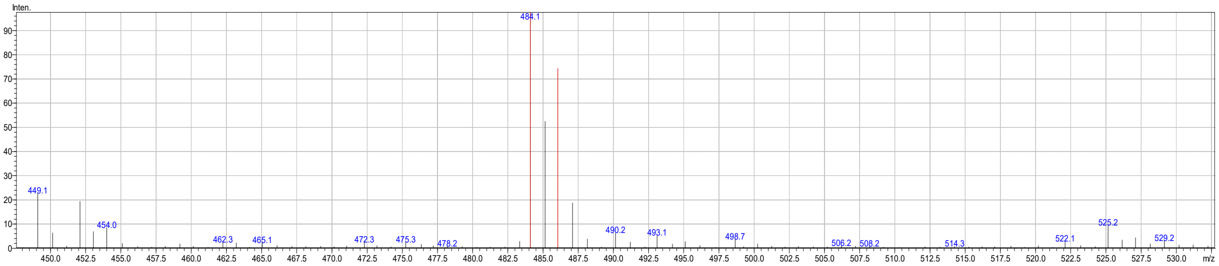

LC-MS spectra for compound **18**, m/z calculated for $C_{26}H_{26}ClNO_6$ $[M+H]^+$ 484.1 found 484.1

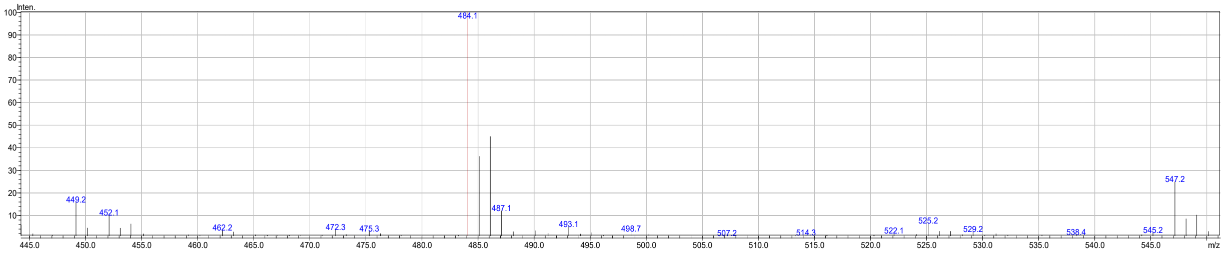

Supplement: Supplementary file 1 [file gels-07-00134-s001.zip › gels-1332731-supplementary.pdf]
